# Development and use of a switchgrass (*Panicum virgatum* L.) transformation pipeline by the BioEnergy Science Center to evaluate plants for reduced cell wall recalcitrance

**DOI:** 10.1186/s13068-017-0991-x

**Published:** 2017-12-22

**Authors:** Richard S. Nelson, C. Neal Stewart, Jiqing Gou, Susan Holladay, Lina Gallego-Giraldo, Amy Flanagan, David G. J. Mann, Hiroshi Hisano, Wegi A. Wuddineh, Charleson R. Poovaiah, Avinash Srivastava, Ajaya K. Biswal, Hui Shen, Luis L. Escamilla-Treviño, Jiading Yang, C. Frank Hardin, Rangaraj Nandakumar, Chunxiang Fu, Jiyi Zhang, Xirong Xiao, Ryan Percifield, Fang Chen, Jeffrey L. Bennetzen, Michael Udvardi, Mitra Mazarei, Richard A. Dixon, Zeng-Yu Wang, Yuhong Tang, Debra Mohnen, Brian H. Davison

**Affiliations:** 1 Noble Research Institute, LLC, Ardmore, OK 73401 USA; 2 Department of Plant Sciences, University of Tennessee, Knoxville, TN 37996 USA; 3 BioEnergy Science Center (BESC), Oak Ridge National Laboratory, Oak Ridge, TN 37831 USA; 4 BioDiscovery Institute and Department of Biological Sciences, University of North Texas, Denton, TX 76203 USA; 5 Complex Carbohydrate Research Center, University of Georgia, Athens, GA 30602 USA; 6 Department of Biochemistry and Molecular Biology, University of Georgia, Athens, GA 30602 USA; 7 Department of Genetics, University of Georgia, Athens, GA 30602 USA

**Keywords:** Reverse genetics, Lignocellulosic feedstocks, Transformation pipeline, Cell wall, Recalcitrance, Lignin, Ethanol, Bioenergy, HCT, XTH

## Abstract

**Background:**

The mission of the BioEnergy Science Center (BESC) was to enable efficient lignocellulosic-based biofuel production. One BESC goal was to decrease poplar and switchgrass biomass recalcitrance to biofuel conversion while not affecting plant growth. A transformation pipeline (TP), to express transgenes or transgene fragments (constructs) in these feedstocks with the goal of understanding and decreasing recalcitrance, was considered essential for this goal. Centralized data storage for access by BESC members and later the public also was essential.

**Results:**

A BESC committee was established to codify procedures to evaluate and accept genes into the TP. A laboratory information management system (LIMS) was organized to catalog constructs, plant lines and results from their analyses. One hundred twenty-eight constructs were accepted into the TP for expression in switchgrass in the first 5 years of BESC. Here we provide information on 53 of these constructs and the BESC TP process. Eleven of the constructs could not be cloned into an expression vector for transformation. Of the remaining constructs, 22 modified expression of the gene target. Transgenic lines representing some constructs displayed decreased recalcitrance in the field and publications describing these results are tabulated here. Transcript levels of target genes and detailed wall analyses from transgenic lines expressing six additional tabulated constructs aimed toward modifying expression of genes associated with wall structure (xyloglucan and lignin components) are provided. Altered expression of *xyloglucan endotransglucosylase/hydrolases* did not modify lignin content in transgenic plants. Simultaneous silencing of two *hydroxycinnamoyl CoA:shikimate hydroxycinnamoyl transferases* was necessary to decrease G and S lignin monomer and total lignin contents, but this reduced plant growth.

**Conclusions:**

A TP to produce plants with decreased recalcitrance and a LIMS for data compilation from these plants were created. While many genes accepted into the TP resulted in transgenic switchgrass without modified lignin or biomass content, a group of genes with potential to improve lignocellulosic biofuel yields was identified. Results from transgenic lines targeting xyloglucan and lignin structure provide examples of the types of information available on switchgrass lines produced within BESC. This report supplies useful information when developing coordinated, large-scale, multi-institutional reverse genetic pipelines to improve crop traits.

**Electronic supplementary material:**

The online version of this article (10.1186/s13068-017-0991-x) contains supplementary material, which is available to authorized users.

## Background

The BioEnergy Science Center (BESC) was one of the three Bioenergy Research Centers (BRCs) funded by the United States Department of Energy from October 2007 through September 2017. Each BRC contained a large contingent of researchers focused on a particular bioenergy research theme http://genomicscience.energy.gov/centers/ [1]. BESC focused on basic and translational research directed toward decreasing cellulosic biofuel production costs. A central strategy to accomplish this is through modification of plant cell walls for easier and cheaper access to sugar substrates (i.e. reduced recalcitrance [2]). Biomass recalcitrance is rooted in the difficulty of degrading plant cell walls for conversion into biofuel [3, 4]. Since its inception, BESC sought to decrease recalcitrance specifically in switchgrass (*Panicum virgatum* L.) and *Populus* spp., as representatives of perennial grass and woody feedstocks.

For multi-institutional projects with vertically integrated goals it is important to focus efforts by prioritizing and codifying plant production, harvest and analysis procedures and establishing centralized data storage. BESC researchers determined to use genetic transformation as one approach to progress from target gene identification to validation of its effect on cell wall recalcitrance. BESC was conceived to include researchers with the knowledge to (i) identify genes with potential to decrease recalcitrance, (ii) clone and express constructs of interest in plants, (iii) grow plants in greenhouse and field trials and analyze tissue from these studies for multiple cell wall traits (i.e. those affecting lignin, pectin, hemicellulose, xylose and cellulose structure) and (iv) interpret results with the aim of deciphering mechanisms to reduce biomass recalcitrance for biofuel production [e.g. 5–8]. This capacity was focused through the formation of a committee, the BESC Transformation Pipeline (TP) Committee, whose function was to create the framework through which candidate constructs would be identified, accepted, evaluated and cataloged for their influence on recalcitrance. Target gene manipulation was expected to yield basic findings important for understanding cell wall synthesis pathways and structures that could impact recalcitrance in addition to applied findings leading to an improved lignocellulosic biofuel crop.

The goal of this report is to provide information on the organization and outcomes of the BESC TP to those interested in plant cell wall recalcitrance in switchgrass for basic and applied purposes. For a select group of target genes we provide gene names, sequence ID and primer sequences used to amplify the target gene or its fragment that was accepted into the BESC TP for switchgrass transformation. For a subset of the select group of target genes, we tabulate and discuss prior publications showing the effect of altering their expression on wall structure and recalcitrance. Genes or gene fragments accepted into the TP whose effect on cell wall traits and plant growth are to be published elsewhere are not included here. In many instances for the select group of genes there was no change in either target gene expression or in measured cell wall traits, but these results are potentially instructive for future research and are included here. We also provide detailed results from cell wall analyses of several transgenic lines targeting two genes thought to influence wall structure [*xyloglucan endotransglucosylase/hydrolase* (*XTH*) and *hydroxycinnamoyl CoA: shikimate hydroxycinnamoyl transferase* (*HCT*)] which demonstrate influences on plant growth and/or lignin content and illustrate data flow within our laboratory information management system (LIMS). The organization of this process will be informative to those pursuing large-scale multi-institutional reverse genetic screens to improve agronomic traits.

## Methods

### BESC TP target gene submission form

The BESC TP committee identified and codified information to include on a submission form to enable the evaluation of gene constructs submitted to the TP. A completed submission form for a construct accepted into the TP is included to illustrate (i) the requested information, (ii) the type of information supplied, and (iii) information available to all BESC researchers participating in the TP process (Additional file 1).

### Genes and constructs

Gene models chosen for manipulation were based on the best gene sequence information available at the time of application to the TP. For genes slated for overexpression early in BESC, there sometimes were more reliable resources for sequences from species with at least a draft genome assembly [e.g. rice (*Oryza sativa*), Arabidopsis (*Arabidopsis thaliana*) and sorghum (*Sorghum bicolor*)] than from switchgrass where no draft genome sequence was available [genomic sequencing of lowland switchgrass ‘Alamo’ genotype AP13 was begun in 2009 by the Joint Genome Institute (Walnut Creek, CA)]. Because of their reliability, these non-switchgrass sequences were used when a switchgrass sequence was not available for some of the overexpression TP submission forms. However, in some instances these sequences from alternative species were replaced by switchgrass sequences as information became available for each target gene. For most overexpression constructions, cDNA produced from transcript was used to clone genes of interest. For RNAi-mediated knockdown, sequences between 200 and 600 bp were chosen to create RNAi hairpin targets to silence individual target genes or a family of genes. The target location within a gene for an individual gene knockdown was generally a short sequence (200–300 nt) near the 3′ end of the open reading frame or, as sequence was available, into or within the 3′ untranslated region: a region with high sequence heterogeneity allowing identification of target sequences specific for individual gene family members [9]. For gene family knockdowns, target sequences were in the more conserved regions of the open reading frames representing the related family members. Nucleotide length differences between constructs for gene family silencing often reflected an effort to ensure that more than two > 18 nt contiguous stretches of perfect complementarity existed between the knockdown gene fragment and all of the target gene family member sequences. A contiguous stretch of 19 nts of perfect complementarity is associated with the minimum length of perfect complementarity necessary to induce RNA silencing [10]. In most cases, expression of target genes for overexpression or gene fragments for knockdown of the target gene(s) was controlled by a strong constitutive promoter. The majority of the genes were placed under the control of the maize ubiquitin 1 (*ZmUbi1*) promoter [11] using selected pANIC plant vectors [12]. Other genes were placed under control of *PvUbi1*, *PvUbi2* or other promoters within the selected vector [13–18]. The pANIC series of vectors are Gateway compatible for overexpression or knockdown via RNAi and were created within BESC as an enablement for switchgrass transformation [12]. The gene names, sequence IDs, and primers used to clone genes or gene fragments can be found in Additional file 2.

To overexpress or silence *XTH*-*like1b* (AP13CTG28985) or *2a* (AP13ISTG54783), EST sequences at the Switchgrass Functional Genomics Server (https://switchgrassgenomics.noble.org/index.php) were used to obtain the full length mRNA sequences. Overexpression of *PvXTH*-*like1b* was addressed by cloning a sequence of 882 nucleotides (positions 184 to 1065 within the genomic sequence) using primers TPC 871 F2 and TPC 871 R4 (Additional files 2 and 3). Knockdown of *PvXTH*-*like1b* expression was pursued through cloning and expression of a 344 bp fragment specific for this gene (Additional file 3). The fragment was cloned using primers TPC 870 F and TPC 870 R (Additional file 2). Overexpression of *PvXTH*-*like2a* was addressed by cloning a sequence of 885 nucleotides (positions 156 to 1038 within the genomic sequence) using primers 864_oe-F1 and 864_oe-R1 (Additional files 2 and 3). Knockdown of *PvXTH*-*like*2a was pursued through cloning and expression of a 273 bp fragment specific for this gene (Additional file 3) using primers 863+70_kd-F3 and 863+70_kd-R3 (Additional file 2). DNA sequences were cloned separately into entry vector pCR8/GW/TOPO (Invitrogen, Carlsbad, CA).

To silence *HCT1* and *HCT2* either individually or together, the following methods were used to clone the gene fragments. Partial EST sequences at the Switchgrass Functional Genomics Server (https://switchgrassgenomics.noble.org/index.php) were used as tools to obtain full-length mRNA sequences of *HCT1* and *HCT2* from switchgrass genotype NFCX1 through 5′- and 3′-RACE, following the manufacturer’s protocols (Invitrogen). The amplified PCR products were ligated into the pGEM-T Easy Vector following the vector manufacturer’s protocol (Promega, Madison, WI) and confirmed by sequencing. To knockdown expression of *HCT1* and *HCT2* individually or simultaneously, three RNAi binary vectors were constructed using the pANIC8A gateway vector [12]. Gene fragments selected from the non-conserved regions of *HCT1* or *HCT2* or the conserved domain (Additional file 4) were amplified by PCR using primers HCT1RNAi-F, HCT1RNAi-R, HCT2RNAi-F, HCT2RNAi-R, HCT1/2RNAi-F, HCT1/2RNAi-R (Additional file 2) and cloned into pENTR/D-TOPO (Invitrogen). Then they were inserted into the pANIC8A vector through the Gateway system (Invitrogen). Constructs verified for correct sequence were used to transform switchgrass genotype NFCX1.

### Switchgrass transformation and characterization

*Agrobacterium*-mediated transformation was conducted on embryogenic callus from transformable ‘Alamo-’derived clones ST1, ST2, SA1, NFCX1 or seed-derived callus of ‘Alamo’ identified within BESC. The methods generally followed those outlined in published papers [19–21]. Putative transgenic events were determined at the tissue culture stage on observation of individual shoots regenerated from different pieces of callus that survived treatment in selection media containing at least 30 mg/L hygromycin B. For most characterizations of transformed plants, plant phenotypes were determined from T0 generation transformants grown in greenhouse experiments. For studies involving HCT, T0 generation transgenic plants selected for further analysis were crossed to genotype ST2 plants to obtain progeny T1 seeds. Both HCT-RNAi-positive and -negative (null segregant) plants were identified from the progeny of each cross by PCR verification of the insertion of the RNAi fragments, and the negative plants were used as controls for analyses of the corresponding T1 HCT-RNAi transgenic plants. All transgenic plants produced in this project were treated as regulated materials and their interstate transport and release into the environment was conducted under the appropriate USDA APHIS BRS permits.

### Gene expression analysis

Reverse transcription-quantitative PCR (RT-qPCR) was performed to determine expression profiles of targeted genes in transgenic switchgrass through standard techniques. For *XTH* and *HCT* genes, switchgrass tissues (as indicated for each reported experiment) were harvested from silenced and control plants at the same developmental stages, immediately frozen in liquid nitrogen and stored at − 80 °C. For *XTH* transcript expression analysis, harvested tissue was internodes at the elongation three (E3) stage [22]. For *HCT* transcript expression analysis, harvested tissue was the top two internodes from young stems at the E3 stage. Total RNA was extracted from tissue stored at – 80 °C with RNAzol RT (Molecular Research Center, Inc., Cincinnati, OH) as instructed by the manufacturer and extracted RNA was subjected to reverse transcription with Superscript III Kit (Invitrogen). SYBR Green (Applied Biosystems, Foster City, CA) was used as the reporter dye. Primer pairs used for RT-qPCR-based transcript analyses were, for example, the following: HCT1 (HCT1qF: 5′-TCAGCTTCGATTTCGGATTT-3′, HCT1qR: 5′-TGATCTCCATCTGCATTCCA-3′), HCT2 (HCT2qF: 5′-ACTTCTGATCGCCTCGACAC-3′, HCT1qR: 5′-CTGTCCTGTGCTGTGCATCT-3′), XTH1b (PvXTH1b_qPCR_F: 5′-CTGCCTGCAACGATCAGCT-3′ and PvXTH1b_qPCR_R: 5′-CTTCTACCAGGACAGGAGATGA-3′) and XTH2a (PvXTH2a_qPCR_F: 5′-ACCCGTGCCTGTCACCACA-3′ and PvXTH2a_qPCR_R: 5′-CACCATTGTTCTTGTAGATCATG-3′). *Ubq1* (GeneBank Accession Number: FL899020) transcript level was used as an internal control for *XTH* and *HCT* expression analysis. Ubiquitin primers were UBQ6-F: 5′-AGAAGCGCAAGAAGAAGACG-3′ and UBQ6-R: 5′-CCACCTTGTAGAACTGGAGCA-3′.

### Root phenotyping for transgenic plants silenced for HCT

The seeds produced from the verified T1 transgenic plants were germinated in ½ MS medium. Individual seedlings were verified to have an integrated RNA silencing fragment through PCR. Ten positive transgenic and control seedlings each were selected and grown in ½ MS medium in clear plastic containers with sufficient area for viewing root architecture. After 1 month of growth, digital images of the roots of each plant were obtained in the presence of a scale bar and quantified using ImageJ 1.63 software (http://rsbweb.nih.gov). The number of emerging crown roots and number and length of lateral roots were measured in a 2-cm section starting at the root tip for each root.

### Determination of lignin content and composition

Lignin content and composition were determined using a high throughput technique [23, 24]. Specifically for *XTH* and *HCT* analyses, whole stem and/or roots were harvested at the R1 stage. The collected samples were ground in liquid nitrogen and lyophilized. The total lignin content and lignin composition of each sample were then determined by the acetyl bromide (AcBr) and thioacidolysis methods, respectively [25, 26].

### Saccharification analyses

Saccharification assays were on greenhouse-grown whole tillers at the R1 developmental stage [24, 27].

### Statistics

For lignin monomer and root trait analyses of transgenic switchgrass silenced for *HCT* expression, ANOVA and Fisher’s least significant difference (LSD) analyses were conducted utilizing R version 3.4.1 [28] and LSD.test function from the R/agricolae package [29]. Means among transgenic lines and controls were compared and differences were considered significant if *p* values were less than or equal to 0.05. Standard deviations are provided in tables and figures.

### Data compilation

Information to be collected for the TP was determined by an internal BESC committee. A commercial software package, Nautilus LIMS™ (Thermo Scientific, Philadelphia, PA), was used for sample information management in BESC and populated with data in selected categories when the information was available. Nautilus is designed for research laboratories and based on configurable workflows using an Oracle™ database with a Windows Explorer-like interface. The Center’s data are secured through a combination of Oracle™ database security and Nautilus LIMS™ application controls and daily backups. The LIMS environment includes Nautilus LIMS™, SAP^®^ InfoMaker for producing barcode labels, Thermo Scientific™ WebAccess Suite™ for remote access, an Apache Tomcat^®^ webserver environment, and Oracle™ relational database.

BioEnergy Science Center sample metadata are managed within the BESC LIMS to ensure identified data are available for future analysis. Minimal metadata guidelines have been developed in conjunction with BESC PIs to determine the minimum set of plant sample metadata necessary for all plant samples. These metadata guidelines are important for data sharing, data archiving and data integration. The BESC LIMS provides all BESC researchers with guidelines and templates for metadata input such as gene sequences and transformation details, to make the capture of metadata part of all data submissions to the LIMS. Access to the results for the 53 constructs reported here will be available at http://bioenergycenter.org/besc/.

## Results and discussion

### Switchgrass transformation pipeline

From the start of BESC in October 2007 through 2012 [submission rounds 1–12 within the TP], 128 constructs representing 88 candidate genes were accepted into the reverse genetics program for transgenesis in switchgrass. The identity of some genes accepted into the TP and the data obtained from the subsequently produced transgenic switchgrass lines are not disclosed in this report because either (a) experiments are still being performed to characterize recalcitrance traits in these lines for commercial benefit or (b) results modifying specific transgene(s) are being incorporated into single- or multi-gene-centric reports with more detailed descriptions of outcomes than can be given here. Thus, results presented here represent a subset of the total number of target genes evaluated with a bias towards genes that were not ultimately pursued for their commercial use. The findings therefore will benefit the research community not only in the identification of genes or gene fragments with potential to decrease cell wall recalcitrance, but also in indicating genes believed to be good candidates for modifying recalcitrance but which subsequently were shown to be ineffective, and in illustrating genes and gene fragments that posed cloning, expression, or plant growth hurdles. This report also will serve as a reference for the TP procedure used to produce and analyze those TP products.

Membership on the TP committee, tasked with creating the TP submission form and accepting TP submissions, required experience in identifying target genes with the potential to modify recalcitrance, producing transformation constructs and transgenic plants, and/or analyzing the effects of construct expression on cell wall synthesis and biomass recalcitrance. Members of this committee [nine BESC principal investigators (PIs)] identified a set of core requirements for submissions to the pipeline. Among the requirements were: (a) name of the candidate gene; (b) cDNA or genomic sequence with accession number, if available; (c) type of expression requested (i.e. stable knockdown, stable overexpression, stable ectopic expression, or transient expression through virus-induced gene silencing (genes submitted through the latter category will be presented in a separate report); (d) evidence that the gene was expressed (e.g. through microarray, RNA-seq, northern blot, RT-PCR analyses); (e) phylogeny showing related genes within applicable gene families and highlighting potential homologs (switchgrass is a polyploid) [30, 31]; (f) shared motifs or domains among members of the gene family; and (g) rationale for submission (i.e. the proposed mechanism for an influence of gene or gene fragment expression on cell wall constitution and how this could directly or indirectly lead to enhanced saccharification and biofuel production from switchgrass). A copy of a completed TP submission form whose construct was accepted into the TP is included (Additional file 1). Through TP submission round 12, a total of 615 submissions for gene overexpression or gene or gene family knockdown in switchgrass, poplar and foxtail millet (*Setaria italica*) were received from BESC member scientists. Information in submissions that were accepted into the TP made a compelling case for overexpressing or knocking down a candidate gene or gene family to decrease recalcitrance without decreasing plant growth and biomass or in providing new information about wall synthesis pathways. A small fraction of genes with unknown function, but expressed at high levels in relevant target tissue such as stems, were also accepted. The submissions that received support from the majority of the TP committee members were accepted for analysis within the TP and the completed submission form was made available to BESC team members tasked with cloning the target gene or gene fragment and transforming plants.

For the 53 constructs included that were approved by the TP committee, 31 were chosen for overexpression of target genes and 22 constructs were chosen for knockdown of target genes via RNAi (Table 1). Multiple genes were targeted for both overexpression and knockdown and nine of these are listed. Many of the target genes were related to lignin biosynthesis (Table 1). Given that both altered S:G (syringyl:guaiacyl) monolignol composition and decreased total lignin in secondary cell walls can increase enzymatic sugar release [32], and therefore potentially lead to decreased recalcitrance, it is not surprising that BESC researchers nominated known lignin biosynthesis genes early in the TP. Indeed, two target genes within the BESC switchgrass TP whose modified expression resulted in significant decreases in cell wall recalcitrance were modified in lignin composition and amount [21, 33–36].

**Table 1:** Compiled subset of constructs approved by the BESC Transformation Committee for overexpression or knockdown of target genes to modify cell wall traits

Gene name	BESC ID	Transgene origin	Expression system	Gene in expression vector	Expression vector	Events^a^	Target expression level^b^	Visual phenotype	Comments	PI^c^	Ref.
*4*-*Coumarate: coenzyme A ligase* (*4CL*)	180	Switchgrass	KD	Yes	pVT1629	100	80% decrease	Brown and red pigment in shoots^d^		2	[48, 62]
*Cinnamic acid 4*-*hydroxylase* (*C4H*)	182	Switchgrass	KD	Yes	pANIC 4B	9	50% decrease	Normal growth	No change in lignin or S/G monomer ratio	2	[48]
*Hydroxycinnamoyl CoA: shikimate hydroxycinnamoyl transferase* (*HCT1*)	184-1^e^	Switchgrass	KD	Yes	pANIC 8A	28	0–95% decrease	Normal growth	Revised to target HCT1. No change in lignin content or composition	7	This report
*Hydroxycinnamoyl CoA: shikimate hydroxycinnamoyl transferase* (*HCT2*)	184-2^e^	Switchgrass	KD	Yes	pANIC 8A	41	0–90% decrease	Normal growth	Revised to target HCT2. Increase in H lignin monomer in majority of transgenic lines analyzed. No change in lignin content.	7	This report
*Caffeoyl CoA 3*-*O*-*methyl transferase* (*CCoAOMT*)	186	Switchgrass	KD	Yes	pANIC8A	17	0–90% decrease	Normal growth	No change in lignin content or composition	7	[48]
*Coumaroyl shikimate 3′*-*hydroxylase* (*C3′H*)	264	Switchgrass	KD	Yes	pANIC 4A	48	0–80% decrease	Normal growth	No change in lignin content or composition	7	
*VirE2*-*interactor protein* (*VIP2*)	266	Arabidopsis	OE	No	Not applicable (see comment)	Not applicable (see comment)	Not applicable (see comments)	Not applicable (see comment)	Gene not clonable	7	
*Arabinosyl transferase 1* (Rra1 reduced residual arabinose)	274	Switchgrass	KD	No	Not applicable (see comment)	Not applicable (see comment)	Not applicable (see comments)	Not applicable (see comment)	Gene not clonable	3	
*Endoxylanase* (PttXyn10A) /*Endoxyla*nase	282	Switchgrass	OE	No	Not applicable (see comment)	Not applicable (see comment)	Not applicable (see comments)	Not applicable (see comment)	Gene not clonable	3	
*LIM transcription factor* (*LIM 1*)	292	Switchgrass	OE	No	Not applicable (see comment)	Not applicable (see comment)	Not applicable (see comments)	Not applicable (see comment)	Gene not clonable	2	
*R2R3*-*MYB transcription factor* (*MYB4*)	294	Switchgrass	OE	Yes	pANIC 2B	5	ninefold to 11-fold increase	Varying impacts on growth, from reduced to increased yield		2	[33, 34, 36]
*Gibberellin 20*-*oxidase* (*GA20*-*ox*)	318	Foxtail millet	OE	Yes	pANIC 10A	3	No change	Normal growth. No change in biomass yield	No change in lignin content, S/G monomer ratio or sugar release	4	
*Gibberellin 20*-*oxidase* (*GA20*-*ox*)	319	Switchgrass	KD	Yes	pANIC 12A	6	No change	Normal growth. No change in biomass yield	No change in lignin content, S/G monomer ratio or sugar release	4	
*NAC transcription factor* (*NAC 2*)	321	Switchgrass	OE	No	Not applicable (see comment)	Not applicable (see comment)	Not applicable (see comment)	Not applicable (see comment)	Gene not clonable	1	
*Peroxidase*-*30*	324	Switchgrass	OE	Yes	pANIC 6A	52	Not done	Normal growth	No change in lignin content or composition	5	
*Dirigent protein*	327	Switchgrass	OE	Yes	pANIC 6A	23	Not done	Normal growth	No change in lignin content or composition	5	
*Dirigent protein*	328	Switchgrass	KD	Yes	pANIC 8A	30	Not done	Normal growth	Full length construct in antisense orientation. No change in lignin content or composition	5	
*Peroxidase*-*1*	343	Switchgrass	FKD	Yes	pANIC 8A	19	Not done	Normal growth	No change in lignin content or composition	5	
*NAC transcription factor* (*NAC*-*AP2*)	348	Switchgrass	OE	Yes	pCAMBIA 1305	65	21-fold to 65-fold increase	Increased biomass yield in greenhouse		6	[63]
*NAC transcription factor* (*NAC*-*AP2*)	349	Switchgrass	FKD	Yes	pSTARGATE	6	No change		Discontinued (regenerated plants were false positive)	6	
*NAC transcription factor* (*NAC 033*)	350	Switchgrass	OE	No	Not applicable (see comment)	Not applicable (see comment)	Not applicable (see comment)	Not applicable (see comment)	Gene not clonable	2	
*Dirigent protein*-*2*	356	Switchgrass	KD	Yes	pANIC 8A	23	Not done	Normal growth		5	
*Sucrose synthase 1* (*SUS1*)	413	Switchgrass	OE	Yes	pANIC 10A	5	twofold to sevenfold increase	Increased plant height, tiller number and biomass yield.	Increased lignin	4	[64]
*Cellulose synthase*-*like*; *subfamily D* (*CslD4*)	540	Switchgrass	OE	Yes	pANIC 10A	10	Not done	Normal growth	Under further analysis	3	
*Cellulose synthase*-*like*; *subfamily J* (*CslJ*)	543	Switchgrass	OE	Yes	pANIC 10A	11	Not done	Normal growth	Under further analysis	3	
*Cellulose synthase*-*like; subfamily F* (*CslF6*)	549	Switchgrass	OE	Yes	pANIC 10A	23	Not done	Normal growth	Under further analysis	3	
*Cellulose synthase*-*like; subfamily F* (*CslF9*)	552	Switchgrass	OE	No	Not applicable (see comment)	Not applicable (see comment)	Not applicable (see comment)	Not applicable (see comment)	Gene not clonable	3	
*Cellulose synthase 8* (*CesA8*)	558	Switchgrass	OE	Yes	pANIC 10A	10	Not done	Normal growth	Under further analysis	4	
*NAC transcription factor* (*NAC*-*AP2*)	692	Switchgrass	OE	Yes	modified pER8	30	No change	Not determined	Discontinued as plants unresponsive to estradiol treatment	6	
*Laccase 4* (*Lac 4*)	693	Switchgrass	KD	Yes	pANIC 8A	30	Not done	Normal growth	No change in lignin amount or composition	5	
*R2R3*-*MYB transcription factor* (*MYB63*-*like2*)	721	Switchgrass	OE	No	Not applicable (see comment)	Not applicable (see comment)	Not applicable (see comment)	Not applicable (see comment)	Gene not clonable	2	
*R2R3*-*MYB transcription factor* (*MYB63*-*like3*)	722	Switchgrass	OE	No	Not applicable (see comment)	Not applicable (see comment)	Not applicable (see comment)	Not applicable (see comment)	Gene not clonable	2	
*Knotted*-*like homeobox protein 1* (*KN1*)	833	Switchgrass	KD	Yes	pANIC 12A	8	No change	Normal growth. No change in biomass yield	Discontinued	4	
*Knotted*-*like homeobox protein 1* (*KN1*)	834	Switchgrass	OE	Yes	pANIC 10A	5	Up to sevenfold increase	Normal growth. No change in biomass yield		4	[65]
*Gibberellin 2*-*oxidase* (*GA2*-*ox*)	835-1^e^	Switchgrass	OE	Yes	pANIC 10A	14	Up to 14-fold increase	Dwarf to semi-dwarf growth		4	[66]
*Gibberellin 2*-*oxidase* (*GA2*-*ox*)	835-2^e^	Switchgrass	OE	Yes	pANIC 10A	7	Up to fourfold increase	Dwarf growth		4	[66]
*Ethylene response factor/SHINE transcription factor* (*ERF/SHN 1*)	837	Switchgrass	OE	Yes	pANIC 10A	6	Up to ninefold increase	Normal growth. Increased biomass dry weight		4	[67]
*UTR6*	838	Switchgrass	FKD	Yes	pANIC 8A	30	18–70% decrease		No consistent correlation in lignin content, S/G monomer ratio or sugar release with gene expression levels	2	
*UTR6*	839	Switchgrass	OE	Yes	pANIC 10A	50	Up to 38-fold increase		No consistent correlation in lignin content, S/G monomer ratio or sugar release with gene expression levels	2	
*Laccase 17*-*like gene A* (*LAC17a*)	844	Switchgrass	FKD	Yes	pANIC 8A	40	No change	Normal growth	No change in lignin content	7	
*Laccase 17*-*like gene A* (*LAC17a*)	845	Switchgrass	OE	Yes	pANIC 6A	40	No change	Normal growth	No change in lignin content	7	
*Laccase 17*-*like gene B* (*LAC17b*)	846	Switchgrass	OE	Yes	pANIC 6A	40	No change	Normal growth	No change in lignin content	7	
*Purple acid phosphatase 2* (*PAP2*)	847	Switchgrass	OE	Yes	pMDC32	40	No change	Normal growth	No change in lignin content	7	
*Caffeoyl CoA 3*-*O*-*methyl transferase 2* (*CCoAOMT2*)	848	Switchgrass	KD	Yes	pANIC 8A	30	40–90% decrease	Normal growth	No change in lignin content.	7	[48]
*Coumaroyl shikimate 3′*-*hydroxylase 2* (*C3′H2*)	849	Switchgrass	KD	Yes	pANDA	30	10–85% decrease	Normal growth	No change in lignin content	7	
*Basic helix*-*loop*-*helix transcription factor* (*bHLH1*)	859	Switchgrass	OE	Yes	pANIC 10A	7	No change	Normal growth. No change in biomass yield	Discontinued	4	
*p*-*coumarate 3*-*hydroxylase 2* (*C3H2*)	861	Switchgrass	KD	No	Not applicable (see comment)	Not applicable (see comment)	Not applicable (see comment)	Not applicable (see comment)	Gene not clonable	2	
*p*-*coumarate 3*-*hydroxylase 2* (*C3H2*)	862	Switchgrass	OE	No	Not applicable (see comment)	Not applicable (see comment)	Not applicable (see comment)	Not applicable (see comment)	Gene not clonable	2	
*Xyloglucan endotransglucosylase/hydrolase* (*XTH*-*like2a*)	863	Switchgrass	KD	Yes	pANIC 8A	30	40–80% decrease	Not observed	No change in lignin content	2	This report
*Xyloglucan endotransglucosylase/hydrolase* (*XTH*-*like2a*)	864	Switchgrass	OE	Yes	pANIC 10A	40	15–70% increase	Semidwarf to normal growth	No change in lignin content	2	This report
*Xyloglucan endotransglucosylase/hydrolase* (*XTH*-*like1b*)	870	Switchgrass	KD	Yes	pANIC 8A	30	10–80% decrease	Semidwarf to normal growth	No change in lignin content	2	This report
*Xyloglucan endotransglucosylase/hydrolase* (*XTH*-*like1b*)	871	Switchgrass	OE	Yes	pANIC 10A	40	20–150% increase	Semidwarf to normal growth	No change in lignin content	2	This report
*Caffeic acid 3*-*O*-*methyltransferase* (*COMT*)	930	Switchgrass	KD	Yes	pANDA	9	90% decrease	Normal growth. Increased biomass yield		7	[21, 35, 47, 48, 68–70]

*ID* identification number, *OE* overexpression, *KD* knockdown, *FKD* family knockdown, *Ref*. References

^a^Number of independent events

^b^Determined by RT-qPCR

^c^Principal Investigator associated with construct: (1) Fang Chen; (2) Richard A. Dixon; (3) Debra Mohnen; (4) C. Neal Stewart, Jr.; (5) Yuhong Tang; (6), Michael Udvardi; (7) Zeng-Yu Wang

^d^Brown coloration in parts of leaf veins; brown patches in stems; reddish-brown coloration on the inner sides of basal stems and brownish color in the mature roots

^e^Homologs of *HCT* or *GA2*-*ox* genes targeted after initial TP submission, designated by dashed numeral after BESC ID number

In 11 instances of the 53 constructs targeted for cloning, the target gene sequences were not clonable (Table 1). Since switchgrass was the source organism for most of these genes and there was no switchgrass reference genome available in the early part of the work, we did not find the number of cloning failures to be inordinate. Most of the remaining genes or gene fragments were successfully expressed in switchgrass, indicating little or no lethality imparted due to construct expression and that the pANIC vector was effective for transformation. An average of 26 putative transgenic events per construct were produced for constructs listed in Table 1 and T0 plants were sent for analysis to the PIs who had submitted the proposals for the specific targeted genes. In a minority of cases, the transformation process was difficult in that transgenic plants representing less than 10 independent transformation events per construct were received by the submitting PI (Table 1). Although BESC researchers greatly improved switchgrass transformation efficiency during rounds 1–12 of the TP, transformation of this species, like that of many crops, remains a slow process (see [37] for a general discussion of crop transformation bottlenecks). In most cases, sufficient numbers of independent transgenic events (three or more per construct) were produced to gauge the potential impact of the expression of the target gene or gene fragment on recalcitrance. Where available, we have noted cell wall and recalcitrance phenotypes of these plants. Acquiring 26 independent transgenic events per construct on average for this many target genes in an experimentally challenging crop required a processing pipeline only made available to a large research center such as BESC.

In order to significantly improve switchgrass as a lignocellulosic feedstock for enzymatic deconstruction [38] or consolidated bioprocessing [39], end-of-season aboveground biomass yield should be maintained or improved and cell wall recalcitrance decreased. Where available, we have noted plant growth phenotypes for the plants (Table 1). For 83% of the target constructs where growth and biomass characteristics were reported, growth and/or biomass yield of the individual transgenic plants was always similar to or greater than the non-transgenic parent (Table 1). Regarding cell wall modification, although 60% of the constructs reported here were expected to affect the lignin pathway, the manipulation of many of these did not appreciably affect lignin content. This result may be explained by the fact that cell wall biosynthesis in angiosperms marshals up to ten percent of the genome, c.a. 2500 genes [40] and many of these single gene targets reside in gene families [41]. Residence in a gene family can be indicative of redundant function between family members (e.g. [42, 43]) and the need to silence expression of multiple family members to achieve a modified cell wall. There are also examples where silencing of a saccharide biosynthesis gene led to altered saccharide or lignin content [44, 45]. Thus, it is possible that silencing genes involved in the synthesis of one polymer may be compensated for by those synthesizing a related or alternative wall polymer. Specific lines modified for expression of genes in different wall synthesis pathways are being crossed to evaluate any possible additive effects that become significant in decreasing wall recalcitrance [46].

### Notable findings regarding altered cell walls and plant growth

Although manipulated expression of multiple genes simultaneously may provide additional decreases in cell wall recalcitrance, results from multiple lines generated in the BESC TP have demonstrated that manipulated expression of single genes can be useful in molecular breeding of switchgrass for cellulosic biofuel production. For example, a gene chosen by BESC for downregulation early in the project (officially incorporated into the TP after round 12, but for which work was begun prior to round 12) was a caffeic acid 3-*O*-methyltransferase (*PvCOMT*; GenBank Accession No. HQ645965), which led to the selection of two transgenic switchgrass lines with lower S/G ratio, lower lignin, and higher sugar release and biofuel yield in greenhouse-grown plants [21]. Importantly, for these transgenic lines greenhouse results translated to the field [35]. After the second year of growth in the field, one transgenic event (COMT KD line 2) had increased biomass and biofuel yield, which was calculated to result in 50% more biofuel per field area over the non-transgenic parent switchgrass ([35, 47]; also see Table 1).

A second notable finding, involving overexpression of the gene *MYB4*, encoding a transcription factor targeting the repression of a suite of lignin biosynthesis genes, was the range of growth phenotypes exhibited in individually transformed switchgrass lines [33, 34]. Greenhouse-grown *MYB4* overexpressing switchgrass lines had a wide range of transgene expression and growth phenotypes. Some greenhouse-grown transgenic lines overexpressing *MYB4* had up to three times greater sugar release and 2.6-fold higher biofuel production compared with the non-transgenic parents. When transplanted into the field in Knoxville, TN, USA, the line with the highest *MYB4* expression, lowest lignin, and highest biofuel yield per gram cell wall residue did not survive the first winter. Furthermore, the highest *MYB4* expressers had poorly developed roots [36]. In contrast, a line with lower levels of *MYB4* transcript produced 63% more aboveground biomass and 32% more biofuel than controls ([33, 34, 36]; also see Table 1). These results demonstrated that growth of the biomass in the field is required to identify lines that retain high performance characteristics under natural environmental conditions.

### Characterization of switchgrass transgenic lines altered for xyloglucan endotransglucosylase/hydrolase (XTH) expression

Through a comprehensive microarray analysis, we found a group of xyloglucan endotransglucosylase/hydrolases (PvXTH-like1a to 1d and PvXTH-like2a to 2c) that were down-regulated during the lignification process in both stem tissue and an induced suspension cell system [48]. *PvXTH*-*like1b* and *PvXTH*-*like2a* genes were then submitted and accepted into the TP and transgenic switchgrass generated (Table 1).

Xyloglucan endotransglucosylase/hydrolases are enzymes involved in the modification of cell wall structure through cleavage and re-joining of xyloglucan molecules in primary plant cell walls [49]. Xyloglucan binds non-covalently to cellulose, coating and cross-linking adjacent cellulose microfibrils, and the resulting extensive xyloglucan-cellulose network is thought to act as the major tension-bearing structure in the primary wall [50]. However, the function of XTHs in monocotyledon secondary cell wall formation is poorly understood.

Full-length mRNA sequences of *XTH1b* and *XTH2a* were isolated from switchgrass. Sequence analysis indicated that there was 61% similarity between *XTH1b* and *XTH2a*, with 29% similarity in their GH16 XET functional domain(s). Based on these characteristics, constructs aimed toward overexpressing and silencing *XTH1a* and *XTH1b* were designed (Additional file 3). Notably, the fragments cloned to silence the *XTH* genes each would be specific for their respective targeted *XTH*, having no more than 16 nucleotide stretches of identity between the two *XTH*s (Additional file 3).

**Fig. 1:**
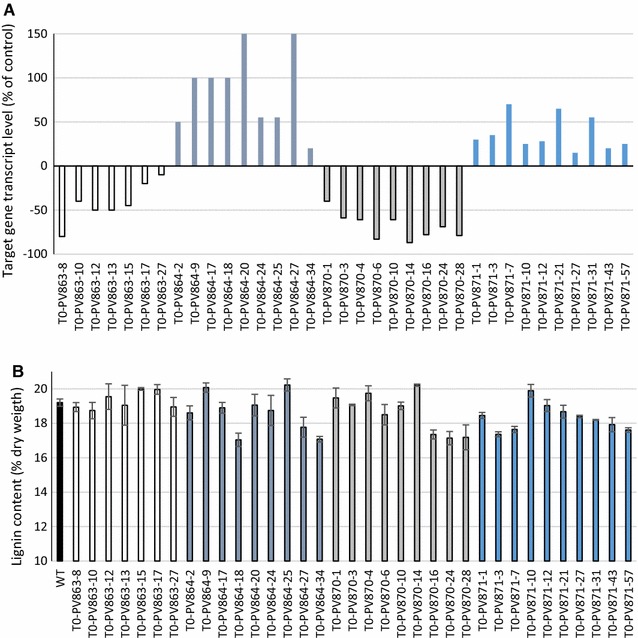
Xyloglucan endotransglucosylase/hydrolase (XTH) transcript level and lignin content in transgenic switchgrass lines silenced for or overexpressing *XTH*-*like1b* or *XTH*-*like2a*. **A** Percent of target gene expression (target gene represents transgene for overexpressing lines and targeted plant gene for silenced lines) relative to control (mean value for three independent control plants) for plants representing listed transgenic lines at E3 stage of development. Expression of *Ubq1* in these plants was analyzed and used to normalize *XTH* expression levels across lines. **B** Lignin content for plants representing listed transgenic lines and control (WT) at R1 stage. Transgenic lines: T0-Pv863-xx (silencing *PvXTH*-*like2a*); T0-Pv864-xx (overexpressing *PvXTH*-*like2a*); T0-Pv870-xx (silencing *PvXTH*-*like1b*); T0-Pv871-xx (overexpressing *PvXTH*-*like1b*)

Forty independently transformed plants expected to overexpress *PvXTH*-*like2a* (T0-Pv864) and 40 plants expected to overexpress *PvXTH*-*like1b* (T0-Pv871) were generated (Table 1). Clonal lines with high transgene transcript abundance compared with control plants were selected for additional analysis (Fig. 1A). Lignin content was reduced in three *PvXTH*-*like2a* overexpression lines (#18, #27 and #34) (Fig. 1B); however, the lignin content level did not correlate with the expression level of the transgene. In the case of *PvXTH*-*like1b* overexpression, reduced lignin content was also observed in seven out of ten plants subjected to the analysis (Fig. 1B), although, again, transgene expression level was not correlated with lignin content.

Thirty independently transformed plants designed to silence *PvXTH*-*like2a* (T0-863) and an identical number of plants designed to silence *PvXTH*-*like1b* (T0-870) also were generated. Through RT-qPCR, a small number of plants that expressed the transgene and were silenced were selected for further analysis (Fig. 1A). Downregulation of *PvXTH*-*like2a* expression resulted in no changes in lignin content (Fig. 1B). For the *PvXTH*-*like1b* silenced plants, three plants out of nine showed reduced lignin content, but as for the overexpressing lines, no correlation with measured transcript level was obtained (Fig. 1B).

The lack of correlation between transgene expression in lines over-expressing the individual *XTH* genes and their lignin content was perhaps unexpected based on the negative relationship between *XTH* expression and lignification in stems and cell cultures [48]. Furthermore, the lack of a correlation between transgene expression in the knockdown lines and lignin content for both *XTH* genes also was unexpected. It is possible that redundancy between *XTH*s compensated for reduced expression of a particular *XTH*. Further work is necessary to understand the influence of XTHs on lignin content in monocotyledonous plants. Clearly, although XTHs function to maintain primary cell wall extensibility during growth, they were not shown to directly impact lignification by the approach taken in the present work.

### Characterization of switchgrass transgenic lines altered for hydroxycinnamoyl CoA: shikimate hydroxycinnamoyl transferase (HCT) expression

Another set of genes accepted into the TP were members of the *HCT* family (Table 1). HCTs are reported to be required in two steps during the conversion of *p*-coumaroyl CoA to guaiacyl (G) and syringyl (S) lignin monomers, but are not involved in the biosynthesis of hydroxyphenyl (H) lignin monomers [32, 51]. Downregulation of HCT in *Nicotiana benthamiana*, *A. thaliana*, alfalfa and Monterey pine (*Pinus radiata*) resulted in high H monomer levels, decreased recalcitrance for sugar release and/or decreased biomass [32, 52–55]. Additionally, silencing *HCT* in *Arabidopsis* and alfalfa [55, 56] showed increased flavonoid biosynthesis and improved drought tolerance and resistance to fungal infection, although plant growth was inhibited. The potential of decreasing recalcitrance in switchgrass by silencing *HCT* homologs was investigated through the TP.

Through a BLAST search using the Switchgrass Functional Genomics Server (https://switchgrassgenomics.noble.org/index.php), two EST sequences, AP13ISTG44531 and AP13CTG44233, were identified as *HCT* genes in switchgrass and named *HCT1* and *HCT2*, respectively. Two full-length mRNA sequences of *HCT1* and *HCT2* were amplified from switchgrass genotype NFCX1. Sequence analysis indicated that there was 62% similarity between *HCT1* and *HCT2*, with higher similarity in their functional domain (domain PLN02663) and less toward the 5′ and 3′-ends of the ORF (Additional file 4). Based on these characteristics, constructs to silence *HCT1* or *HCT2* were designed. Additionally, a fragment within the *HCT* conserved domain was amplified to silence both genes (Additional file 4).

**Fig. 2:**
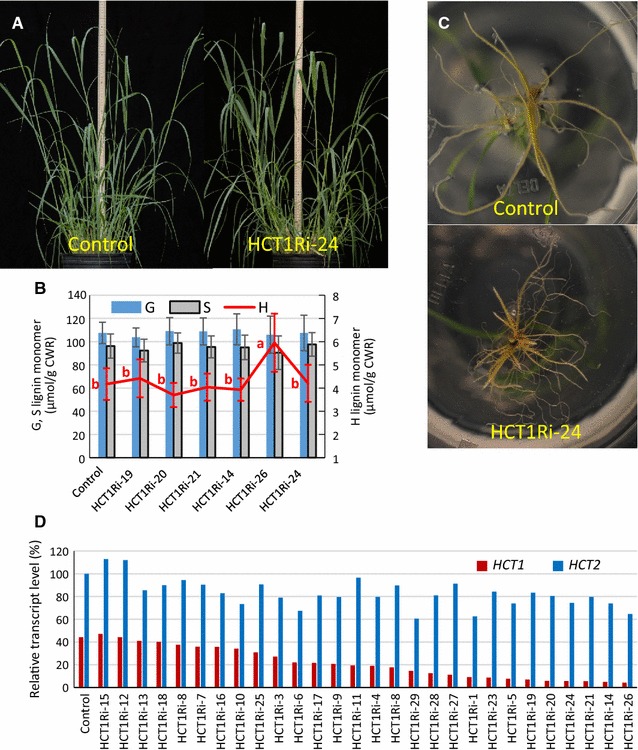
Visual and molecular phenotypes of *HCT1*-*RNAi* transgenic plants (HCT1Ri). **A** T1 generation null segregant (Control; left) and HCT1Ri transgenic (HCT1Ri-24 line, right) plants at R1 stage of development. **B** Lignin monomer content in stems of plants from null segregant (control) and HCT1Ri lines sampled at R1 stage. Values represent mean ± S.D. of three biological (plant) replicates. For G and S monomer levels, no significant differences were seen between lines at the 0.05 significance level. For G and S monomer levels, no significant differences were observed between lines at the 0.05 significance level. Non-identical letters in red indicate significant difference in the H monomer levels between two lines at the 0.05 level determined by ANOVA and least significant difference (LSD) test. Guaiacyl (G), syringyl (S) and *p*-hydroxyphenyl (H) lignin monomers were measured. *CWR* cell wall residue. **C** Root architecture of 1-month-old plants. **D** Relative transcript levels of *HCT1* and *HCT2* in control and HCT1Ri plants representing 28 independent transformation events at 1 month post sub-tillering. Expression of *Ubq1* in these plants was analyzed and used to normalize *HCT* expression levels across lines. Relative treatment level values (vertical axis) are normalized to the HCT2 control value set at 100%

Twenty-eight transgenic plants produced to silence *HCT1* (HCT1Ri) expression were analyzed. No transgenic plant displayed a visible phenotype in the aerial tissue (Fig. 2A). *HCT1* expression levels were dramatically decreased in plants representing these T0 HCT1Ri lines with generally minimal influence on *HCT2* expression (Fig. 2D). The minimal effect on *HCT2* expression in these transgenic lines demonstrated the specificity of the *HCT1* silencing construct to influence *HCT1* and not *HCT2* expression. Based on these RT-qPCR results, six transgenic lines with more than 90% reduction in *HCT1* transcript level were selected for further analysis (i.e. lines HCT1Ri-14, 19, 20, 21, 24 and 26). Lignin content and lignin monomer composition were determined for whole stem tissue (leaf and sheath removed) of T1 generation plants at the R1 developmental stage [22] for each transgenic line and a line that had lost the inserted sequence through segregation (i.e. a null segregant control). There was no significant difference in G and S monomer content between the HCT-silenced and control plants (Fig. 2B). H monomer content also was unchanged in the HCT-silenced lines with one exception, HCT1Ri-26, which was the most silenced of the HCTRi lines studied and where H monomer content was increased (Fig. 2B).

**Fig. 3:**
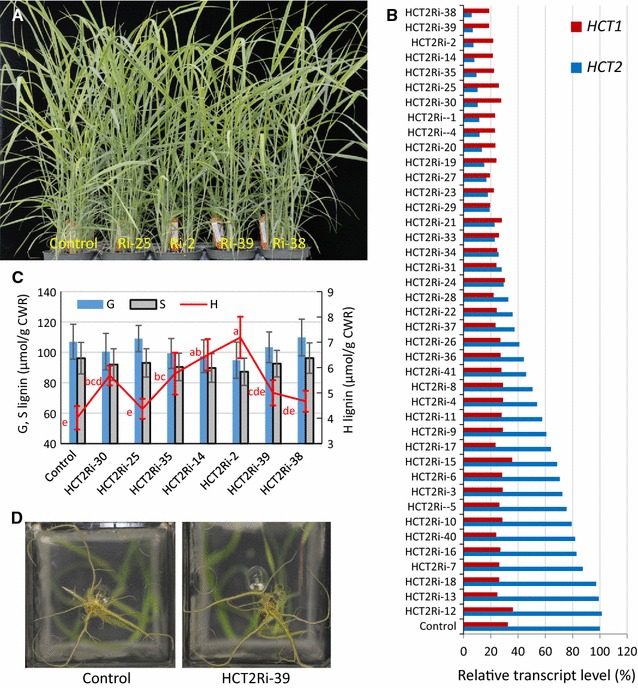
Visual and molecular phenotypes of *HCT2*-*RNAi* transgenic plants (HCT2Ri). **A** NFCX1 wild type (WT) (Control; left) and *HCT2*-*RNAi* transgenic plants (Ri-25, -2, -39 and -38 lines, right) at R1 stage. **B** Relative transcript levels of *HCT1* and *HCT2* in control and HCT2Ri plants representing 41 independent transformation events at 1 month post sub-tillering. Expression of *Ubq1* in these plants was analyzed and used to normalize *HCT* expression levels across lines. Relative treatment level values (horizontal axis) are normalized to the HCT2 control value set at 100%. **C** Lignin monomer content in stems of WT control and *HCT2*-*RNAi* transgenic plants (HCT2Ri) at R1 stage. Values represent mean ± S.D. of three biological replicates. For G and S monomer levels, no significant differences were observed between lines at the 0.05 significance level. Non-identical letters in red indicate significant difference in the H monomer levels between two lines at the 0.05 level determined by ANOVA and least significant difference (LSD) test. Guaiacyl (G), syringyl (S) and *p*-hydroxyphenyl (H) lignin monomers were measured. *CWR* cell wall residue. **D** Root architecture of 5-week-old plants

Subsequently, 41 transgenic plants targeted for *HCT2* downregulation (HCT2Ri) were produced. Similar to the *HCT1* downregulated plants, there was no alteration in the visible growth phenotype of HCT2Ri aerial tissue compared with the parent control (Fig. 3A). *HCT2* transcript expression was dramatically decreased in multiple lines while *HCT1* expression was unaffected (Fig. 3B). Seven transgenic lines with more than 90% reduction in *HCT2* transcript level were selected for lignin monomer analysis (i.e. lines HCT2Ri-2, 14, 25, 30, 35, 38 and 39). Similar to findings with *HCT1* downregulated plants, there was no difference in G and S monomer levels in *HCT2* downregulated plants, but unlike results from the majority of HCT1 downregulated plants, there was an increase in H lignin monomers in most HCT2Ri lines compared with the parental control (Fig. 3C).

**Fig. 4:**
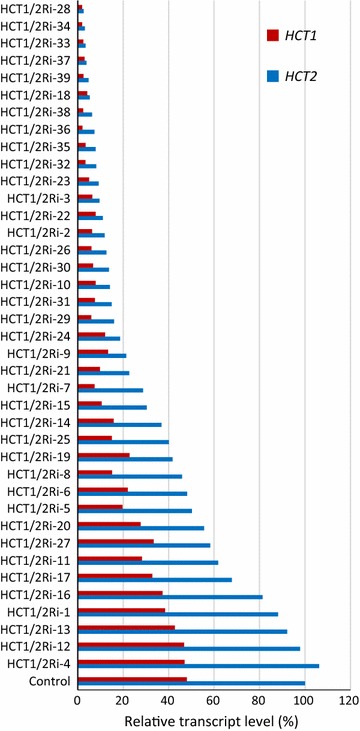
Relative *HCT1* and *HCT2* transcript levels in 39 independently-transformed plants targeted for simultaneous downregulation of *HCT1* and *2* (HCT1/2Ri-xx) at 1 month post sub-tillering. Expression of *Ubq1* in these plants was analyzed and used to normalize *HCT* expression levels across plants. Relative treatment level values (horizontal axis) are normalized to the HCT2 control value set at 100%

Considering that silencing *HCT1* and *HCT2* individually did not alter G and S lignin monomer units, a construct was created to determine if silencing both genes simultaneously would lead to an expected change in lignin monomer content. This construct was submitted to the TP after the 12th round and demonstrates the process whereby the TP committee reviewed and accepted constructs in later rounds that more extensively studied specific target gene family function based on results from plant lines expressing individual family members accepted in TP rounds 1–12. Thirty-nine plants transformed with a gene fragment having multiple regions of 100% identity for greater than 21 contiguous nucleotides between *HCT1* and *HCT2* (HCT1/2Ri) were analyzed. Both *HCT1* and *HCT2* transcript levels were decreased more than 90% in approximately half of the transgenic plants compared with the parental control (Fig. 4). Three lines were shown to have more than a 94% decrease in *HCT1* and *HCT2* expression in a repeat analysis and these lines were selected for further study (i.e. lines HCT1/2Ri-28, 34 and 37).

**Fig. 5:**
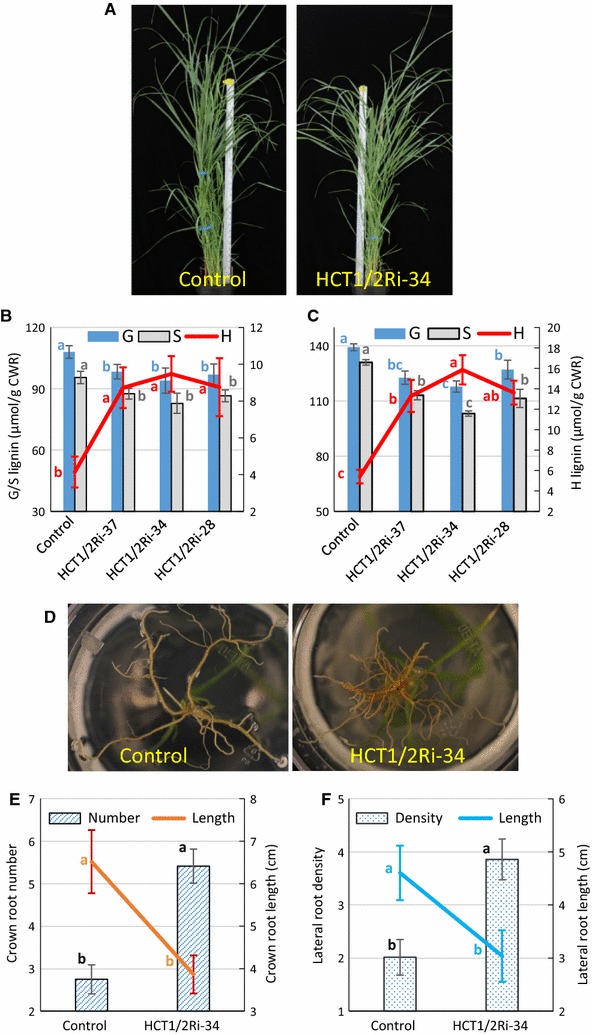
Visual and molecular phenotypes of *HCT1/2*-*RNAi* transgenic plants (HCT1/2Ri-xx) simultaneously downregulated for *HCT1* and *2* expression. **A** NFCX1 wild type (WT) (Control; left) and HCT1/2Ri transgenic (HCT1/2Ri-34 line, right) plants at R1 stage. **B** Lignin monomer content in stems of WT and HCT1/2Ri plants at R1 stage. Guaiacyl (G), syringyl (S) and *p*-hydroxyphenyl (H) lignin monomers were measured. **C** Lignin monomer content in roots of WT and HCT1/2Ri plants at R1 stage. **D** Root architecture of 1-month-old plants. **E** Comparison of crown root number (per plant) and length (cm) in 1-month-old WT and HCT1/2Ri plants. **F** Comparison of lateral root density (lateral roots number/1 cm main root) and length (cm) in 1-month-old WT and HCT1/2Ri plants. Values represent mean ± S.D. of three biological replicates. *CWR* cell wall residue. Non-identical letters in corresponding colors in the charts (**B**, **C**, **E**, **F**) indicate significant differences in analyzed trait between treatments at the 0.05 level determined by ANOVA and least significant difference (LSD) test

**Fig. 6:**
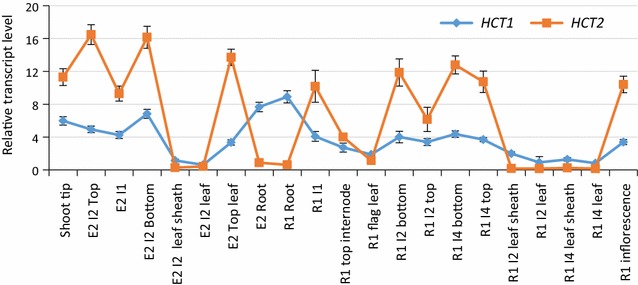
Expression profiles of *HCT1* and *HCT2* in various tissues at different stages in NFCX1 wild type (WT). *E2* elongation stage 2, *R1* reproductive stage 1, *I1* internode 1, *I2* internode 2, *I4* internode 4. Values represent mean ± S.D. of three biological replicates. Expression of *Ubq1* in these plants was analyzed and used to normalize *HCT* expression levels across tissues

There was little visible difference in the aerial organs between control and HCT1/2Ri plants except in plant height. All three transgenic lines were consistently slightly shorter than the parental control at the R1 stage (e.g. Fig. 5A). HCT1/2Ri aerial (stem) tissue displayed moderate differences in lignin monomer content with both G and S monomers decreased and the H monomer increased compared with respective monomer levels in the parental control (Fig. 5B). Compiling G, S and H lignin monomer levels, the three HCT1/2Ri lines were decreased 5–8% in total lignin content in stems. Interestingly, HCT1/2Ri plants also displayed significant differences in root architecture. HCT1/2Ri plants had increased crown root numbers and root densities, and shorter root length than the parental plant (Fig. 5D–F). There also was a brown color correlated with the increase in root numbers and root densities (Fig. 5D); the brown color suggesting the accumulation of flavonoids similar to findings from *Arabidopsis* and alfalfa where HCT activity was downregulated [55, 56]. It will be important to determine if higher flavonoid accumulation in roots of HCT1/2Ri plants occurs and whether it is correlated with greater abiotic and biotic stress resistance in switchgrass [57]. A similar effect on visible root structure was observed when silencing *HCT1* alone (Fig. 2C), but not *HCT2* alone (Fig. 3D), suggesting that the effect on root structure was predominantly due to silencing *HCT1* expression. Interestingly, *HCT1* expression was observed to predominate over *HCT2* expression in root tissue (Fig. 6), correlating well with the greater influence of *HCT1* expression over *HCT2* expression on root structure. As in the aerial tissue, G and S lignin monomers were decreased and H lignin monomers increased in the HCT1/2Ri root tissue compared with the parental control root tissue. Compiling G, S and H lignin monomer levels, the three HCT1/2Ri lines were decreased 7–12% in total lignin content in roots (Fig. 5C): a difference greater than that observed in aerial tissue.

These findings indicate that, in switchgrass, silencing multiple HCTs was necessary to begin to mimic the enhanced H and minimal G and S monomer accumulation exhibited by alfalfa after silencing HCT expression [52]. The switchgrass results begin to define activities of individual HCTs in specific plant tissues. Findings here suggest redundant functions for *HCT1* and *HCT2* in aerial switchgrass tissue. However, redundancy between these genes may be less complete in roots where a modified growth phenotype was apparent when silencing only *HCT1*, which predominates in expression over *HCT2* in root tissue. The relationship between the root growth phenotype and lignin content in root cells, especially when downregulating *HCT1*, requires further study. A second conclusion from these studies is that the aerial growth phenotype was only modestly affected when silencing switchgrass *HCT1* or *HCT2* expression alone or both *HCT*s simultaneously. This finding may indicate that additional *HCT*s with similar function in aerial tissue exist in switchgrass. Four *HCT*s have been identified in the grass model plant, rice [58]. Rice is a self-fertilizing diploid, while lowland switchgrass is an outcrossing tetraploid species. Taken together these findings suggest that there could be at least 4 *HCT* homologs in switchgrass, and further indicate the complexity associated with modifying a polyploid species to obtain a measurable influence on cell wall structure.

### Compilation of data from the TP

Considering that hundreds of BESC researchers were producing data on thousands of feedstock and microbe samples, it was imperative to capture this information in a centralized system that provided simplified access by multiple researchers within BESC. Specific information detailing construct formation, plant transformation, plant growth and cell wall trait analysis was captured within the LIMS. Completed submission forms (e.g. Additional file 1) were captured as PDFs within the BESC-based Wiki website. Additionally, a committee was formed within BESC to identify the types of data to accumulate and store to provide details of construct synthesis, plant transformation, plant growth and cell wall trait analyses. Tables were added to the Oracle database to store gene names, gene models, expression type, primer sequences, gene and gene fragment sequences, recipient plant transformation vectors, plant transformation status, plant growth parameters and cell wall traits (a portion of this information is found in Table 1). A unique BESC identification barcode for each construct was used to link individual information (e.g. gene sequence) pertaining to each construct. BESC team members were able to share information within BESC based on the parent institute’s signature on a mutual Material Transfer Agreement (MTA). All information available in the BESC LIMS for constructs described in this manuscript can be accessed at http://bioenergycenter.org/besc/.

## Conclusions

Part of the BESC mission to produce feedstock lines for basic and applied studies in biofuel production was addressed by (a) organizing a TP to identify and select target genes that could alter cell wall structure in perennial feedstocks for better biofuel production, and (b) implementing a pipeline to produce transgenic plants altered in expression of these target genes, analyze tissue, and centralize data storage. Within this report, these activities are detailed in the context of evaluating transgenic switchgrass growth, wall traits and recalcitrance for basic and applied goals.

Among constructs accepted into the TP during rounds 1–12 because of their hypothesized importance for biofuel production, some were found to be difficult to express or had no impact on measured cell wall traits in transgenic plants. Most members of the latter group were not selected for in-depth analysis. Difficulties cloning or expressing particular sequences or a lack of effect on cell wall traits in transgenic lines expressing a specific sequence are, however, useful results instructive to researchers considering future target sequences for cell wall modification. To illustrate this point, detailed analysis of the effect on lignin monomer and/or lignin content and plant growth traits for transgenic lines modified for expression of individual *HCT* genes was described. While no influence on lignin G and S monomer accumulation was observed after silencing two individual *HCT*s, simultaneous knockdown of these genes demonstrated that family silencing could result in modified lignin G and S monomer content and less total lignin in aerial tissue, but poor root growth.

For a smaller number of genes, there were measurable differences in cell wall traits within the transgenic lines and these modified wall traits were correlated with reduced recalcitrance of the tissue. Findings published for some of these lines are tabulated and summarized in this report. These lines are undergoing advanced cell wall and growth trait studies. Modified expression of regulatory genes, such as transcription factors, more often yielded measurable qualitative differences in traits than when specific pathway genes were modified in expression. Also, it is clear that perturbing cell wall biosynthesis does not necessarily lead to decreased biomass and may lead to more biomass compared with control tissue.

All information from these studies was captured for public dissemination through a LIMS. Thus, the target genes and transgenic plants reported here provide the research community new information when attempting to identify gene candidates for improved recalcitrance or when comparing results with those from BESC studies. Certainly, researchers could consider plant crosses based on the growth and recalcitrance phenotypes reported for these transgenic lines. Additionally, considering that these plants were heterozygous for all constructs, researchers may begin additional silencing studies by employing CRISPR/Cas9 technology to completely downregulate all homoeologous or sufficiently homologous target genes within switchgrass [59], as has been approached in another allopolyploid species, wheat [60, 61]. The BESC switchgrass TP approach proved to be an example of a successful organizational model to study the role of target genes in cell wall biosynthesis and recalcitrance that can be used in part or in its entirety when considering methods and targets for manipulation of other traits in other species.

## Additional files

**Additional file 1.** Example of a completed and accepted BESC TP submission form. The completed form illustrates the information requested of each submitter and an example of the response necessary for the BESC TP Committee to evaluate the worth of the submission for inclusion in the TP. In this instance submission 461, requesting overexpression of *Sucrose synthase 1* (SUS1) to decrease switchgrass biomass recalcitrance for biofuel conversion, was accepted as BESC construct ID number 413.

**Additional file 2.** Switchgrass TP primers for listed genes.

**Additional file 3.** Alignment of *PvXTH*-like genes selected for transformation and location of unique sequences for silencing individual family members. Diagram of Pv*XTH* family member sequences and position of sequence fragments used to silence individual Pv*XTH*s in switchgrass. Unique *PvXTH-like1b* for silencing *XTH-1b* expression is indicated in the red box and unique *PvXTH-like2a* sequence for silencing *XTH-2a* expression is indicated in blue box. Areas with identity between the sequences are highlighted.

**Additional file 4.** Diagram illustrating position of three RNAi construct sequences used to silence *HCT*s in switchgrass. Sequences amplified and expressed in switchgrass to silence *HCT1* (HCT1RNAi, purple box), *HCT2* (HCT2RNAi, red box), and *HCT 1* and *2* together (HCT1/2RNAi, blue box) are shown within the aligned *PvHCT* sequences. Areas with identity between the sequences are highlighted.
