# Supplementary material for: Development and use of a switchgrass (Panicum virgatum L.) transformation pipeline by the BioEnergy Science Center to evaluate plants for reduced cell wall recalcitrance
Source: Biotechnol Biofuels. 2017 Dec 22;10:309. doi: 10.1186/s13068-017-0991-x (PMC5740764; doi:10.1186/s13068-017-0991-x)
Supplement: Supplementary file 1 — Additional file 1. Example of a completed and accepted BESC TP submission form. The completed form illustrates the information requested of each submitter and an example of the response necessary for the BESC TP Committee to evaluate the worth of the submission for inclusion in the TP. In this instance submission 461, requesting overexpression of Sucrose synthase 1 (SUS1) to decrease switchgrass biomass recalcitrance for biofuel conversion, was accepted as BESC construct ID number 413. [file 13068_2017_991_MOESM1_ESM.pdf]

CONFIDENTIAL

Submission No. 461 **Date of submission:** 01/19/2010 12:52:01 pm

Information necessary for consideration of candidate genes for inclusion in the *Populus*/Switchgrass transformation pipeline (BESC)

Requester: David Mann

Laboratory (e.g., PI): Neal Stewart

Institution: UTK

Checklist Criteria:

Is this a resubmission? No

1) Information on Gene Model, Protein ID and Gene Name (if function is unknown, state "unknown"):

Populus

Gene Model:

Gene Name:

Protein ID:

Switchgrass or alternative grass species or non-plant species name: Switchgrass, Foxtail millet

Gene Model:

Gene Name: Panicum virgatum Sucrose synthase 1 (PvSUS1)

Protein ID:

2) Submitting checklists for homologous genes? No

Gene Model Name:

3) Are homologs of this gene currently accepted in the Transformation Pipeline?

Yes

Gene Model Name: Ext_fgenesh4_pg.C_280066 Populus Sucrose synthase

4) Expression type:	Family knockdown	Individual gene knockdown	overexpression

a) *Populus*

	Family knockdown	Individual gene knockdown	overexpression	VIGS Family knockdown	VIGS individual knockdown
b)Switchgrass[2]			✓		

5) Rationale for each desired gene and construct as important to recalcitrance-associated targets.

5.1) Construct Type: Overexpression

Rationale: Sucrose synthase is a well-studied enzyme which converts sucrose and UDP into UDP-glucose and fructose (Kruger, 1990). Sucrose synthase besides its soluble form is also associated with the plasma membrane (Carlson et al., 1996) where it is linked to the synthesis of cell wall polysaccharides callose and cellulose through the formation of UDP-glucose which is the precursor of cellulose (Amor et al., 1995; Subbaiah et al., 2001). Over expression of sucrose synthase has been shown to increase glucose and fructose levels in tobacco (Coleman et al., 2006) which are the precursors for cellulose in cell walls leading to the formation of higher amounts of cellulose (Amor et al., 1995); however, no significant increase in starch levels was found (Coleman et al., 2006). More importantly, over expression of sucrose synthase has been shown to increase cellulose content in Acetobacter xylinum with modified mung bean sucrose synthase (Nakai et al., 1999) and suppression of sucrose synthase has decreased cellulose in carrots (Tang et al., 1999). Also sucrose synthase over expression increased plant height and total biomass (Coleman et al., 2006). Sucrose synthase over expression increases the amount of glucose and fructose. Additionally, Duncan et al. found three sucrose synthase genes in maize (ZmSUS1, ZmSUS-SH1, ZmSUS2). ZmSUS1 and ZmSUS-SH1 are membrane bound while ZmSUS2 is not. However, ZmSUS1 is one of the membrane bound forms which is predominantly expressed during stem elongation and has the VLSRLHSVR motif necessary for membrane association (Hardinet al. 2004). Therefore, the switchgrass sucrose synthase gene most closely related to ZmSUS1, termed PvSUS1, has been selected in this proposal. Over expressing PvSUS1 in switchgrass will cause changes in the structural properties of the cell wall by increasing the amount of cellulose, which may elucidate a better understanding of cell wall biosynthesis and ultimately result in higher cellulose content of the biomass, a desirable trait for the production of ethanol.

5.2) Construct Type:

Rationale:

5.3) Construct Type:

Rationale:

5.4) Construct Type:

Rationale:

5.5) Construct Type:

Rationale:

5.6) Construct Type:

Rationale:

5.7) Construct Type:

Rationale:

5.8) Construct Type:

Rationale:

6) Provide evidence that this gene is expressed:

***Populus*:**

Switchgrass: According to microarray data from Genevestigator, maize SUS1 (GI:514945, NP_001105323.1) is expressed during seedling stage and stem elongation stage at high levels and at lower levels during fruit formation, with the lowest expression at dough stage. 306 ESTs from Panicum virgatum were used to make a tentative contig TC36733 in DFCI gene indices at Harvard University. Out of these 163 ESTs were from switchgrass apex and stem, 24 from root, 23 from early floral buds and reproductive tissue and 9 were from late flowering buds and seed development

Other Species:

7) Provide cDNA or genomic sequence(s) with accession number for each gene, if available.

i)Populus genomic sequence

ii)Populus cDNA

iii)monocot genomic DNA

iv)monocot cDNA

8.1)Submitted multiple sequence alignment of Nucleotide sequences of the gene family:

```
CLUSTAL 2.0.11 multiple sequence alignment


SoSUS2_34391403      ATG---GCTGCCAAG-----------TTRACTCGCCTCCACAGTCTTCGCGAACGCCTT
OsSUS2_16905492      ATG---GCTGCCAAG-----------CTAGCTCGCCTCCACAGTCTCCGCGAACGCCTC
```

```
OsSUS3_1196836      ATGGGGGAAACTACTGGAGAACGTGCCCTGAACCGTCTCCACAGCATGAGGGAGCGCATC
ZmSUS1_162460680    ATGGGGGAAGGTGCAGGTGACCGTGTCCTGAGCCGCCTCCACAGCGTCAGGGAGCGCATT
BoSUS1_62865492     ATGGGGGAAGCTGCCGGCGACCGTGTCCTGAGCCGCCTCCACAGCGTCAGGGAGCGCATC
SbSUS2_222876000    ATG---GCTGCCAAG------------TTGACTCGCCTCCACAGTCTTCGCGAACGCCTT
LpSUS_88687740      ATG---GCCGCCAAA------------CTGACTCGTCTCCACAGTCTCAGGGAGCGGCTT
BoSUS4_55741122     ATG---GCTGCCAAG------------CTGACTCGCCTCCACAGTCTCCGTGAGCGCCTC
BoSUS2_17980242     ATG---GCTGCCAAG------------CTGACTCGCCTCCACAGTCTCCGTGAGCGCCTC
BoSUS3_17980240     ATGGGGGAAACTGCCGGCGACCGTGTCCTGAGCCGCCTCCACAGCGTGAGGGAGCGCATC
PvSuS2-TC34561      ATG---GCTGCCAAG------------CTGACTCGCCTCCACAGTCTTCGCGAACGACTT
PvSuS1-TC36733      ATGGGGGAAGCTGCCGGCGACCGCGTCCTGAGCCGCCTCCACAGCGTCAGGGAGCGCATC
ZmSuSy2_162458267   ATGTCTGCGCCGAAG------------CTGGACCGCAACCCGAGCATCCGCGACCGCGTC
                    ***                         *         **  **  *     * *** *

SoSUS2_34391403     GGTGCCACCTTCTCCTCTCATCCCAATGAGCTGATTGCACTCTTCTCCAGGTATGTTAAC
OsSUS2_16905492     GGTGCCACCTTCTCGTCTCATCCCAATGAGTTGATTGCACTCTTCTCTAGGTATGTTAAC
OsSUS3_1196836      GGCGATTCCCTCTCCGCGCACACCAATGAGCTTGTGGCTGTCTTCTCAAGGCTTGTGAAC
ZmSUS1_162460680    GGCGACTCACTCTCTGCCCACCCCAATGAGCTTGTCGCCGTCTTCACCAGGCTGAAAAAC
BoSUS1_62865492     GGCGATTCCCTCTCCGCGCACCCCAATGAGCTTGTTGCCGTCTTCACGAGGCTGGTCAAC
SbSUS2_222876000    GGTGCCACCTTCTCCTCTCATCCCAATGAGCTGATTGCACTCTTCTCCAGGTATGTTAAC
LpSUS_88687740      GGTGCCACCTTCTCCTCCCATCCCAATGAGCTCATTGCTCTGTTTTCCAGGTATGTTCGC
BoSUS4_55741122     GGTGCCACCTTCTCCTCTCATCCTAATGAGCTGATTGCACTATTTTCCAGGTATGTTAAC
BoSUS2_17980242     AGTGCCACCTTCTCCTCTCATCCTAACGAGCTGATTGCACTGTTCTCCAGGTATGTTCAC
BoSUS3_17980240     GGCGATTCCCTCTCCGCCCACCCCAATGAGCTCGTCGCTGTCTTCACGAGGCTGGTCAAC
PvSuS2-TC34561      GGTGCCACCTTCTCCTCTCATCCCAATGAGCTGATTGCACTCTTTTCCAGGTATGTTAAC
PvSuS1-TC36733      GGTGACTCCCTATCTGCTCACCCCAATGAGCTCGTCGCTGTCTTCACCAGGCTGAAAAAC
ZmSuSy2_162458267   GAGGACACCCTCCACGCGCACCGCAACGAGCTCGTCGCCCTCCTGTCCAAGTACGTGAAC
                           *  *       **    **  ** *  * **  *  *  * *        ***

SoSUS2_34391403     CAGGGCAAGGGAATGCTTCAGCGCCATCAACTGCTTGCTGAGTTTGATGCCCTGTT----
OsSUS2_16905492     CAGGGAAAGGGAATGCTCCAGCGTCACCAGCTGCTTGCGGAGTTCGATGCCTTGAT----
OsSUS3_1196836      CAAGGAAAGGGAATGCTACAGCCCCACCAGATCATTGCTGAGTACAACGCCGCAATCCCT
ZmSUS1_162460680    CTTGGAAAGGGTATGCTGCAGCCCCACCAGATCATTGCCGAGTACAACAATGCGATCCCT
BoSUS1_62865492     CTTGGAAAGGGAATGCTGCAGCCCCACCAGATCATCGCTGAGTACAACAACGCAATCCCT
SbSUS2_222876000    CAGGGCAAGGGAATGCTTCAGCGCCATCAACTGCTTGCTGAGTTTGATGCCCTGTT----
LpSUS_88687740      CAGGGCAAAGGCATGCTTCAGCGCCACCAGCTGCTCGTTGAGTTTGACGCCCTGTT----
BoSUS4_55741122     CAGGGCAAAGGAATGCTTCAGCGTCACCAGCTGCTTGCTGAGTTCGATGCCCTTAT----
BoSUS2_17980242     CAGGGCAAAGGAATGCTTCAACGCCACCAGCTGCTTGCTGAGTTTGACGCCCTGAT----
BoSUS3_17980240     CTTGGAAAGGGAATGCTGCAGCCCCACCAGATCATCGCTGAGTACAACAATGCAATCCCT
PvSuS2-TC34561      CAGGGCAAGGGAATGCTTCAGCGCCATCAACTGCTTGCTGAGTTTGATGCGCTGTT----
PvSuS1-TC36733      CTTGGAAAGGGCATGCTGCAGCCCCACCAGATCATTGCTGAGTACAACAGCGCTATCCCT
ZmSuSy2_162458267   AAGGGGAAGGGCATCCTGCAGCCGCACCACATCCTCGACGCGCTCGACGAGGTCCAGGGC
                       ** ** ** ***** ** *  ** **  *  *    *      *

SoSUS2_34391403     -----TGATAGTGACAAGGAGAAGTATGCGCCCTTCGAAGACTTTCTTCGYGCTGCTCAG
OsSUS2_16905492     -----CGAAGCTGACAAAGAGAAATATGCTCCCTTTGAAGACATTCTCCGGGCTGCTCAG
OsSUS3_1196836      GAGGGCGAGCGTGAGAAGCTGAAGGACTCTGCCTTAGAGGATGTCCTGAGGGGAGCACAG
ZmSUS1_162460680    GAGGCTGAGCGCGAGAAGCTCAAGGATGGTGCTTTTGAGGATGTCCTGAGGGCAGCTCAG
BoSUS1_62865492     GAGGCAGAGCGTGAGAAGCTGAAGGATGGTGCCTTTGAGGATGTCCTGAGGGCAGCACAG
SbSUS2_222876000    -----TGATAGTGACAAGGAGAAGTATGCGCCCTTCGAAGACTTTCTTCGTGCTGCTCAG
LpSUS_88687740      -----CGAATCCGACAAGGAGAAGTATGCTCCTTTTGAAGACATCCTCCGTGCTGCCCAG
BoSUS4_55741122     -----GGATGCTGACAAGGAGAAGTATGCACCCTTTGAAGACATTCTCCGTGCTGCTCAG
BoSUS2_17980242     -----TGCTGCTGACAAGGAGAAGTATGCACCCTTTGAAGACATTCTCCGTGCTGCTCAG
BoSUS3_17980240     GAGGCAGAGCGTGATAAGCTGAAGGATGGCGCCTTTGAGGATGTCCTGCGGGCAGCACAG
PvSuS2-TC34561      -----TGATAGTGACAAGGAGAAGTATGCACCCTTTGAAGACTTTCTTCGTGCTGCTCAG
PvSuS1-TC36733      GAGGCTGAGCGCGAGAAGCTGAAGGATGGTGCCTTTGAGGACGTCCTGAGGGCAGCTCAG
ZmSuSy2_162458267   TCCGGGGGCCGCGCGCTAGCCGAGGGAC---CCTTCCTCGACGTCCTCCGCTCCGCGCAG
                                *         *        ****        *  *           **
```

```
SoSUS2_34391403     GAAGCAATTGTGCTCCCTCCCTGGATAGCACTTGCTATCAGGCCAAGGCCTGGTGTCTGG
OsSUS2_16905492     GAAGCCATTGTGCTGCCGCCCTGGGTTGCACTGGCCATCAGGCCAAGGCCTGGTGTCTGG
OsSUS3_1196836      GAGGCGATTGTCATCCCTCCATGGATTGCCCTTGCCATTCGCCCAAGGCCTGGTGTCTGG
ZmSUS1_162460680    GAGGCGATTGTCATCCCCCCATGGGTTGCACTTGCCATCCGCCCTAGGCCTGGTGTCTGG
BoSUS1_62865492     GAGGCGATCGTTATCCCCCCATGGGTTGCCCTTGCCATCCGCCCGAGGCCTGGTGTCTGG
SbSUS2_222876000    GAAGCAATTGTGCTCCCCCCCTGGGTAGCACTTGCTATCAGGCCAAGGCCTGGTGTCTGG
LpSUS_88687740      GAAGCAATTGTGCTGCCCCCATGGGTTGCACTTGCCATCAGGCCCAGGACTGGTGTCTGG
BoSUS4_55741122     GAAGCAATTGTGCTGCCCCCCTGGGTTGCACTTGCCATCAGGCCGAGGCCTGGTGTCTGG
BoSUS2_17980242     GAAGCAATTGTGCTGCCCCCCTGGGTTGCACTTGCCATCAGGCCAAGGCCTGGTGTCTGG
BoSUS3_17980240     GAGGCGATCGTTATCCCCCCATGGGTTGCCCTTGCCATCCGCCCGAGGCCTGGTGTCTGG
PvSuS2-TC34561      GAAGCGATTGTGCTTCCCCCCTGGGTTGCACTTGCTATCAGGCCAAGGCCTGGTGTCTGG
PvSuS1-TC36733      GAAGCTATTGTTATCCCCCCATGGGTTGCACTCGCCATCCGCCCTAGGCCTGGTGTCTGG
ZmSuSy2_162458267   GAGGCGATCGTGCTGCCGCCGTTCGTGGCCATCGCGGTGCGCCCGCGCCCGGGAGTTTGG
                    ** ** ** **  * ** ** *   * **  * **  *  * **  *  * ** ** ***

SoSUS2_34391403     GATTACATTCGAGTGAATGTAAGCGAGTTGGCTGTGGAGGAGCTGAGTGTTTCTGAGTAC
OsSUS2_16905492     GACTACATTCGGGTGAATGTAAGTGAGTTGGCAGTGGAAGAGCTGAGTGTTTCTGAGTAC
OsSUS3_1196836      GAGTATCTGAGGATCAATGTAAGCCAGCTTGGTGTTGAGGAGCTGAGTGTCCCTGAATAC
ZmSUS1_162460680    GAGTATGTGAGGGTCAACGTCAGTGAGCTCGCTGTTGAGGAGCTGAGAGTTCCTGAGTAC
BoSUS1_62865492     GAGTACGTGAGGGTCAACGTGAGCGAGCTCGCTGTTGAGGAGCTGAGAGTCCCCGAGTAC
SbSUS2_222876000    GATTACATTCGAGTGAATGTAAGCGAGTTGGCTGTGGAGGAGCTGAGTGTTTCTGAGTAC
LpSUS_88687740      GACTACATTCGGGTGAATGTTAGCGACTTGGCTGTGGAAGAGCTGACTGTTTCTGAGTAC
BoSUS4_55741122     GACTACATACGGGTGAATGTTAGTGAGTTGGCTGTGGAGGAGCTGAGTGTTTCTGAGTAC
BoSUS2_17980242     GACTACATACGGGTGAATGTTAGTGAGTTGGCTGTGGAGGAGCTGAGCGTTTCTGAGTAC
BoSUS3_17980240     GAGTATGTGAGGGTCAACGTGAGCGAGCTCGCTGTTGAGGAGTTGAGAGTCCCTGAGTAC
PvSuS2-TC34561      GACTACATTCGGGTGAATGTAAGTGAGTTGGCTGTGGAAGAGCTGAGTGTTTCTGAATAC
PvSuS1-TC36733      GAGTATGTGAGGGTCAATGTCAGCGAGCTCGCGGTTGAGGAGCTGAGAGTCCCTGAGTAC
ZmSuSy2_162458267   GAGTACGTCCGCGTCAACGTTCACGAGCTCAGCGTCGAGCAGCTCACAGTCTCGGAGTAC
                    ** **  *  *  * ** **     *  *    ** **  ** * *  **  * ** ***

SoSUS2_34391403     TTGGCATTCAAGGAACAGCTGGTGGATGGAAATTCCAACAGCAACTTTGTTCTTGAGCTT
OsSUS2_16905492     TTGGCATTCAAGGAACAGCTTGTTGATGGACACACCAACAGCAACTTTGTTCTTGAGCTT
OsSUS3_1196836      TTGCAGTTCAAGGAGCAGCTTGTGGATGGAAGCACCCAGAACAACTTTGTGCTTGAGCTG
ZmSUS1_162460680    CTGCAGTTCAAGGAACAGCTTGTGGAAGAAGGCCCCAACAACAACTTTGTTCTTGAGCTG
BoSUS1_62865492     TTGCAGTTCAAGGAACAGCTTGTGGAAGGAAGCACCAACAACAACTTTGTTCTTGAGCTG
SbSUS2_222876000    TTGGCATTCAAGGAACAGCTGGTGGATGGAAATTCCAACAGCAACTTTGTGCTTGAGCTT
LpSUS_88687740      TTGGCATTCAAGGAGCAGCTTGTTGAAGAGCATGCCAGCAGGAAATTTGTGCTTGAGCTT
BoSUS4_55741122     TTGGCATTCAAGGAACAGCTTGTTGATGGACACACCAACAGCAACTTTGTGCTTGAGCTT
BoSUS2_17980242     TTGGAATTCAAGGAACAGCTTGTTGATGGACACACCAACAGCAACTTTGTGCTTGAGCTT
BoSUS3_17980240     TTGCAGTTCAAGGAACAGCTTGTGGAAGGAAGCACCAATAACAACTTTGTGCTTGAGCTG
PvSuS2-TC34561      TTGGCATTCAAGGAACAGCTGGTGGATGGACACAACAACAGCAACTTTGTGCTTGAGCTT
PvSuS1-TC36733      CTGCAGTTCAAGGAACAGCTTGTGGAAGAAGGCCCCAACAACAACTTTGTTCTTGAGCTG
ZmSuSy2_162458267   CTCCGCTTCAAGGAGGAGCTTGTCGACGGCCAGCACAATGATCCCTACGTTCTCGAGCTT
                     *    ********  **** ** ** *       *         *  ** ** *****

SoSUS2_34391403     GATTTTGAGCCCTTCAATGCCTCATTCCCTCGTCCTTCCATGTCAAAGTCCATTGGAAAT
OsSUS2_16905492     GATTTTGAGCCCTTCAATGCCTCCTTCCCGCGCCCGTCCATGTCCAAGTCCATCGGAAAT
OsSUS3_1196836      GACTTTGAGCCATTCAATGCCTCCTTCCCTCGCCCATCGTTGTCGAAGTCTATTGGCAAT
ZmSUS1_162460680    GACTTTGAGCCATTCAATGCCTCCTTCCCCCGTCCTTCTCTGTCAAAGTCCATTGGCAAT
BoSUS1_62865492     GACCTTGAGCCTTTCAATGCCTCCTTCCCTCGTCCTTCTCTGTCGAAGTCCATTGGCAAC
SbSUS2_222876000    GATTTTGAGCCCTTCAATGCCTCGTTCCCTCGTCCTTCCATGTCAAAGTCCATTGGGAAT
LpSUS_88687740      GATTTTGAGCCTTTCAATGCCTCGGCCCCACGCCCTTCCATGTCCAAGTCCTACGGAAAA
BoSUS4_55741122     GATTTTGAGCCCTTCAATGCCTCCTTCCCGCGTCCTTCCATGTCCAAGTCCATTGGAAAT
BoSUS2_17980242     GATTTTGAGCCCTTCAATGCCTCCTTCCCACGTCCTTCAATGTCCAAGTCCATCGGAAAT
BoSUS3_17980240     GACTTTGAGCCGTTCAATGCCTCCTTCCCTCGTCCTTCTCTGTCGAAGTCCATTGGCAAC
PvSuS2-TC34561      GACTTTGAGCCCTTCAATGCCTCATTCCCTCGTCCTTCCATGTCAAAGTCCATTGGAAAT
```

```
PvSuS1-TC36733      GACTTCGAGCCATTCAATGCATCCTTCCCCCGTCCTTCTCTGTCCAAGTCCATTGGCAAT
ZmSuSy2_162458267   GACTTCGAGCCGTTCAATGTCTCCGTCCCACGCCCAAATCGGTCATCATCTATTGGAAAC
                    **  * ***** *******  **   *** ** **      ***    **    ** **

SoSUS2_34391403     GGAGTGCAATTCCTTAACCGACACCTGTCTTCCAAGTTGTTCCAGGACAAGGAGAGCCTG
OsSUS2_16905492     GGGGTGCAGTTCCTTAACCGTCACCTGTCGTCCAAGTTGTTCCAGGACAAGGAGAGCCTC
OsSUS3_1196836      GGGGTGCAGTTCTTGAACAGGCACCTGTCGTCAAAGCTGTTCCATGACAAAGAGAGCATG
ZmSUS1_162460680    GGCGTGCAGTTCCTCAACAGGCACCTGTCATCAAAGCTCTTCCATGACAAGGAGAGCATG
BoSUS1_62865492     GGTGTGCAGTTCCTCAACAGGCACCTGTCATCAAAGCTCTTCCATGATAAGGAGAGCATG
SbSUS2_222876000    GGAGTGCAATTCCTTAACCGACACCTGTCTTCCAAGTTGTTCCAGGACAAGGAGAGCCTG
LpSUS_88687740      GGGGTGCAGTTCCTTAACCGTCACTCGTCTTCCAAGTTGTTCCAGGACAAGGAGAGCCTC
BoSUS4_55741122     GGGGTGCAGTTCCTTAATCGTCACCTGTCTTCCAAGTTGTTCCAGGACAAGGAGAGCCTC
BoSUS2_17980242     GGGGTGCAGTTCCTTAACCGTCACCTGTCTTCCAAGTTGTTCCAGGACAAGGAGAGCCTC
BoSUS3_17980240     GGTGTGCAGTTCCTCAACAGGCACCTGTCATCAAAGCTCTTCCATGACAAGGAAAGCATG
PvSuS2-TC34561      GGAGTACAGTTCCTTAACAGGCACCTGTCTTCCAAGTTGTTTCAGGACAAGGAGAGCTTG
PvSuS1-TC36733      GGTGTGCAGTTCCTCAACAGGCACCTGTCGTCAAAGCTCTTCCATGACAAGGAGAGCATG
ZmSuSy2_162458267   GGTGTGCAGTTCCTCAACCGACACTTGTCCTCAATCATGTTCCGCAACAGGGATTGCTTG
                    ** ** ** *** * **  * ***  *** ** *   * ** *   * *  **  ** *

SoSUS2_34391403     TACCCATTGCTGAATTTCCTCAAAGCCCATAACTACAAGGGCACGACGATGATGTTGAAT
OsSUS2_16905492     TACCCCTTGCTGAACTTCCTGAAAGCCCATAACCACAAGGGCACGACAATGATGCTGAAT
OsSUS3_1196836      TACCCCCTGCTCAACTTTCTTCGTGCGCACAACTACAAAGGGATGACCATGATGTTGAAC
ZmSUS1_162460680    TACCCCTTGCTCAACTTCCTTCGCGCCCACAACTACAAGGGGATGACCATGATGTTGAAC
BoSUS1_62865492     TACCCCTTGCTCAACTTCCTTCGCGCGCACAACTACAAGGGCATGACTATGATGTTGAAC
SbSUS2_222876000    TACCCATTGCTGAATTTCCTCAAAGCCCATAACTACAAGGGCACGACGATGATGTTGAAT
LpSUS_88687740      TACCCACTGCTCAACTTCCTGAAAGGGCATAACTACAAGGGCACGACAATGATATTGAAC
BoSUS4_55741122     TACCCCCTGCTGAACTTCCTGAAAGCTCATAACCCCAAGGGCAAAACAATGATGCTGAAC
BoSUS2_17980242     TACCCCCTGCTGAACTTCCTGAAAGCCCATAACCACAAGGGCACGACAATGATGCTGAAC
BoSUS3_17980240     TACCCCTTGCTCAACTTCCTTCGTGCACACAACTACAAGGGCATGACTATGATGTTGAAC
PvSuS2-TC34561      TACCCCCTGCTGAACTTTCTCAAAGCCCATAACTACAAGGGCACGACAATGATGTTAAAT
PvSuS1-TC36733      TACCCCCTGCTCAACTTCCTTCGCGCCCACAACTACAAGGGGATGACCATGATGTTGAAC
ZmSuSy2_162458267   GAGCCCCTGTTGGATTTCCTCCGTGGCCACCGGCACAAGGGGCATGTTATGATGCTTAAT
                     * **  ** *  * ** **    *  **      *** **       *****  * **

SoSUS2_34391403     GACAGAATTCAGAGCCTCCGTGGGCTCCAGTCATCCCTTAGAAAGGCAGAAGAGTATCTA
OsSUS2_16905492     GACAGAATTCAGAGCCTTCGTGGGCTCCAATCATCCCTTAGAAAGGCAGAAGAATATCTG
OsSUS3_1196836      GACAGGATTCGCAGTCTCGATGCTCTCCAAGGTGCATTGAGGAAGGCAGAAAAACATCTT
ZmSUS1_162460680    GACAGAATCCGCAGTCTCAGTGCTCTGCAAGGTGCGCTGAGGAAGGCTGAGGAGCACCTG
BoSUS1_62865492     GACAGAATCCGCAGTCTCAGTGCTCTCCAAGGTGCTCTGAGGAAGGCTGAGGAGCATCTG
SbSUS2_222876000    GACAGAATTCAGAGCCTCCGTGGGCTCCAGTCATCCCTTAGAAAGGCAGAAGAGTATCTA
LpSUS_88687740      GACAGAATTCAGAGCCTTCGTGGTGTCCAGTCAGCCCTTAGGAAGGCAGAAGAGTATCTA
BoSUS4_55741122     GACAGAATTCAGAGCCTTCGTGGGCTCCAATCAGCCCTTAGAAAGGCTGAAGAGTATCTC
BoSUS2_17980242     GACAGGATTCAGAGCCTTCGTGGACTCCAATCAGCCCTTAGAAAGGCAGAAGAGTATCTA
BoSUS3_17980240     GACAGAATCCGCAGCCTCAGTGCTCTCCAAGGTGCTCTGAGGAAGGCTGAGGAGCATCTG
PvSuS2-TC34561      GACAGAATTCAGAGTCTTCGCGGGCTCCAGTCATCCCTCAGAAAGGCAGAGGAGTACCTA
PvSuS1-TC36733      GACAGAATCCGCAGCCTCAGTGCTCTCCAAGGTGCTCTGAGGAAGGCTGAGGAGCATCTG
ZmSuSy2_162458267   GATAGAATACAAAGCTTGGGGAGGCTTCAGTCTGTGCTGACCAAAGCTGAGGAGCACTTG
                    ** ** ** *  **  *        * **        * *  ** ** **  *  *  *

SoSUS2_34391403     CTGAGTGTCCCTCAAGACACTCCCTACTCAGAGTTCAACCATAGGTTCCAAGAGCTTGGC
OsSUS2_16905492     ATGGGCATTCCTCAAGACACGCCCTACTCGGAGTTCAACCACAGGTTCCAAGAGCTCGGT
OsSUS3_1196836      GCAGGCATTACAGCTGACACCCCATATTCAGAGTTCCATCACAGGTTCCAAGAGCTTGGT
ZmSUS1_162460680    TCCACCCTACAAGCTGATACCCCATACTCTGAATTTCACCACAGGTTCCAGGAACTTGGT
BoSUS1_62865492     TCTGGTCTTTCAGCAGACACCTCGTACTCGGACTTCCACCACAGATTCCAGGAACTTGGT
SbSUS2_222876000    CTGAGTGTCCCTCAAGACACTCCCTACTCAGAGTTCAACCATAGGTTCCAAGAGCTTGGC
LpSUS_88687740      GTTAGCATCCCTGAAGACACTCCCAGCTCTGAATTCAACCACAGGTTCCAAGAGCTTGGT
BoSUS4_55741122     ATAAGCATTCCTCAGGACACCCCCTGCTCAGAGTTCAACCACAGGTTCCAAGAGCTCGGC
```

```
BoSUS2_17980242     ATGAGCTTTCCTCAGGACACCCCCTACTCAGAGTTCAACCACAGGTTCCAAGAGCTCGGC
BoSUS3_17980240     TCTGGTCTTTCAGCAGACACCCCGTACTCGGATTTCCACCACAGGTTCCAGGAACTTGGT
PvSuS2-TC34561      CTGAGCATCCCTCAAGATACTCCATACTCAGAGTTCAACCACAGGTTCCAAGAGCTCGGC
PvSuS1-TC36733      TCCAGCCTTCCAGCTGATACCCCATACTCTGACTTCCACCACAGATTCCAGGAGCTTGGT
ZmSuSy2_162458267   TCAAAGCTCCCTGCTGACACACCATACTCACAATTTGCTTATAAATTTCAAGAGTGGGGC
                           *       ** **  *    **  * **     * *  ** ** **    **

SoSUS2_34391403     TTGGAGAAGGGTTGGGGTGACACTGCAAAGCGTGTACTTGATACACTCCACTTGCTTCTT
OsSUS2_16905492     TTGGAGAAGGGTTGGGGTGACTGTGCAAAGCGTGTGCTTGACACCATCCACTTGCTTCTT
OsSUS3_1196836      TTGGAGAAGGGTTGGGGTGACTGCGCTCAGCGAGTGCGTGAGACTATTCACCTTCTCTTG
ZmSUS1_162460680    CTGGAGAAGGGTTGGGGTGATTGCGCTAAGCGTGCACAGGAGACTATCCACCTCCTCTTG
BoSUS1_62865492     CTGGAGAAGGGTTGGGGTGATTGTGCCAAGCGTGCGCAGGAGACCATCCACCTCCTCTTG
SbSUS2_222876000    TTGGAGAAGGGTTGGGGTGACACTGCAAAGCGCGTACTTGACACACTCCACTTGCTTCTT
LpSUS_88687740      TTGGAGAAGGGTTGGGGTGACACCGCAAAGCGTGTACACGACACCATCCATTTGCTTCTG
BoSUS4_55741122     TTGGAGAAGGGTTGGGGTGACACTGCAAAGCGTGTACTTGACACCATCCACTTGCTTCTC
BoSUS2_17980242     TTGGAGAAGGGTTGGGGTGACACCGCAAAGCGTGTACTTGACACCATCCACTTGCTTCTC
BoSUS3_17980240     CTGGAGAAGGGTTGGGGTGACTGTGCCAAGCGTGCGCAGGAGACCATTCACCTCCTCTTG
PvSuS2-TC34561      TTGGAGAAAGGTTGGGGTGACACCGCAAAGCGTGTACTTGACACACTCCACTTGCTTCTT
PvSuS1-TC36733      CTGGAGAAGGGTTGGGGTGACTGCGCTAAGCGTGCGCAGGAGACTATTCACCTCCTCTTG
ZmSuSy2_162458267   CTGGAGAAAGGTTGGGGTGATACAGCAGGACATGTTTTGGAAATGATCCATCTCCTTCTA
                     ******* ***********    **    *  *     ** *   * **  * **  *

SoSUS2_34391403     GACCTTCTTGAGGCCCCTGATCCTGCCAACTTGGAGAAGTTCCTTGGAACTATACCAATG
OsSUS2_16905492     GACCTTCTTGAGGCCCCTGATCCGGCCAACTTGGAGAAGTTCCTTGGAACTATTCCAATG
OsSUS3_1196836      GACCTTCTTGAGGCCCCTGAGCCGTCCGCCTTGGAGAAGTTCCTTGGAACAATCCCAATG
ZmSUS1_162460680    GACCTCCTGGAGGCCCCAGATCCGTCCACCCTGGAGAAGTTCCTTGGAACGATCCCCATG
BoSUS1_62865492     GACCTTCTTGAGGCCCCTGATCCGTCCACCCTGGAGAAGTTCCTTGGAACAATCCCAATG
SbSUS2_222876000    GACCTTCTTGAGGCCCCTGATCCTGCCAACTTGGAGAAGTTCCTTGGAACTATACCAATG
LpSUS_88687740      GACCTTCTTGAGGCCCCTGACCCTGCCAGCTTGGAGAAGTTCCTTGGAACTATTCCGATG
BoSUS4_55741122     GATCTTCTTGAGGCCCCCGATCCGGCCAACTTGGAGAAGTTCCTTGGAACTATACCAATG
BoSUS2_17980242     GATCTTCTTGAGGCCCCTGATCCGGCCAACTTGGAGAAGTTCCTTGGAACTATACCAATG
BoSUS3_17980240     GACCTTCTTGAGGCCCCTGATCCGTCCACCCTGGAGAAGTTCCTTGGAACAATCCCAATG
PvSuS2-TC34561      GACCTTCTTGAGGCTCCTGATCCTGCCAACTTGGAGAAGTTCCTTGGAACTATGCCTATG
PvSuS1-TC36733      GACCTTCTTGAGGCCCCAGATCCGTCCACCCTGGAGAAGTTCCTTGGGACTATCCCCATG
ZmSuSy2_162458267   GACATCATTCAGGCGCCAGACCCATCTACCCTAGAGAAATTCTTGGGGAGGATCCCCATG
                    **  *  *  **** ** ** **  *   * * ***** *** * ** *  ** ** ***

SoSUS2_34391403     ATGTTCAATGTTGTTATCCTGTCTCCTCATGGCTACTTTGCCCAATCCAATGTGCTTGGA
OsSUS2_16905492     ATGTTCAATGTTGTTATCCTGTCTCCGCATGGATACTTTGCCCAATCCAATGTGTTGGGA
OsSUS3_1196836      GTGTTCAATGTTGTTATCCTCTCCCCGCATGGTTACTTTGCACAGGCTAATGTCTTGGGG
ZmSUS1_162460680    GTGTTCAATGTCGTTATCCTCTCCCCTCATGGTTACTTCGCTCAAGCTAATGTCTTGGGT
BoSUS1_62865492     GTGTTCAATGTTGTCATCCTCTCCCCGCATGGTTACTTTGCCCAAGCCAATGTCTTGGGG
SbSUS2_222876000    ATGTTCAATGTTGTTATCCTGTCTCCTCATGGCTACTTTGCCCAATCCAATGTGCTTGGA
LpSUS_88687740      ATGTTCAATGTTGTCATCCTGTCTCCCCATGGATACTTTGCTCAATCCAATGTGTTGGGA
BoSUS4_55741122     ACGTTCAATGTTGTTATCCTGTCTCCACATGGCTACTTTGCCCAATCCAATGTGTTGGGA
BoSUS2_17980242     ACGTTCAATGTTGTTATCCTGTCTCCACACGGCTACTTTGCCCAATCCAATGTGTTGGGA
BoSUS3_17980240     GTGTTCAATGTTGTCATCCTCTCCCCACATGGTTACTTTGCCCAAGCCAATGTCTTGGGG
PvSuS2-TC34561      ATGTTTAATGTTGTTATCCTGTCTCCACATGGCTACTTTGCCCAATCCAATGTACTTGGA
PvSuS1-TC36733      GTGTTCAATGTTGTTATCCTCTCCCCCCATGGTTACTTCGCTCAAGCTAATGTCTTGGGT
ZmSuSy2_162458267   ATTTTTAACGTTGTTGTGGTATCCCCTCATGGATACTTTGGTCAAGCTAATGTATTAGGC
                       ** ** ** **  *  * ** ** ** ** ***** *  **  * *****  * **

SoSUS2_34391403     TACCCTGACACTGGTGGTCAGGTTGTGTACATTTTGGATCAAGTCCGTGCTTTGGAGAAT
OsSUS2_16905492     TACCCTGATACTGGTGGTCAGGTTGTGTACATTTTGGACCAAGTCCGCGCTTTGGAGAAT
OsSUS3_1196836      TACCCTGATACCGGTGGGCAGGTTGTCTACATTTTGGATCAAGTCCGTGCTATGGAGAAT
ZmSUS1_162460680    TACCCTGACACCGGAGGCCAGGTTGTCTACATCTTGGATCAAGTGCGCGCTATGGAGAAC
BoSUS1_62865492     TACCCTGACACTGGAGGGCAGGTTGTCTACATTTTGGATCAAGTCCGTGCTATGGAGAAT
```

```
SbSUS2_222876000    TACCCTGACACTGGTGGCCAGGTTGTGTACATTTTGGATCAAGTCCGTGCTTTGGAGAAT
LpSUS_88687740      TACCCTGATACCGGTGGCCAGGTTGTGTACATCTTGGATCAAGTCCGTGCTTTGGAGAAT
BoSUS4_55741122     TACCCTGACACCGGTGGTCAGGTTGTGTACATTTTGGATCAAGTACGCGCTTTGGAGAAT
BoSUS2_17980242     TACCCTGATACCGGTGGTCAGGTTGTGTACATTTTAGATCAAGTCCGCGCTCTGGAGAAT
BoSUS3_17980240     TACCCTGACACCGGAGGGCAGGTTGTCTACATTTTGGATCAAGTCCGTGCTATGGAGAAT
PvSuS2-TC34561      TACCCTGATACGGGTGGTCAGGTTGTGTACATTTTGGACCAAGTCCGTGCTTTGGAGAAT
PvSuS1-TC36733      TACCCTGACACTGGAGGCCAGGTTGTCTACATTTTGGATCAAGTCCGCGCTATGGAGAAT
ZmSuSy2_162458267   TTGCCAGACACAGGAGGACAGATCGTCTATATACTGGACCAAGTCCGTGCACTAGAAAAT
                    *  ** ** ** ** ** *** * ** ** **  * ** ***** ** **  * ** **

SoSUS2_34391403     GAGATGCTTCTTAGGATTAAGCAGCAAGGCCTTGACATCACCCCGAAGATCCTCATTGTT
OsSUS2_16905492     GAGATGCTTTTGAGGATCAAGCAGCAAGGCCTTGATATCACACCTAAGATCCTCATTGTA
OsSUS3_1196836      GAGATGCTGCTGAGGATCAAGCAACAAGGTCTAAACATCACACCAAGGATTCTCATTGTG
ZmSUS1_162460680    GAAATGCTGCTGAGGATCAAGCAGTGTGGTCTTGACATCACGCCGAAGATCCTTATTGTC
BoSUS1_62865492     GAGATGCTGCTGAGGATCAAGCAGCAAGGTCTCAACATCACACCACGGATTCTTATTGTC
SbSUS2_222876000    GAGATGCTTCTTAGGATTAAGCAGCAAGGCCTTGACATCACCCCGAAGATCCTCATTGTT
LpSUS_88687740      GAGATGCTTCTGAGGATTAAGCAGCAAGGCCTTGACATAACTCCTAAGATCCTCATTGTA
BoSUS4_55741122     GAGATGCTTCTGAGGATCAAGCAGCAAGGCCTTGACATCACCCCTAAGATCCTGATTGTA
BoSUS2_17980242     GAGATGCTTCTGAGGATCAAGCAGCAAGGCCTTGACATCACCCCTAAGATCCTCATTGTA
BoSUS3_17980240     GAGATGCTGCTGAGGATCAAGCAGCAAGGTCTCAACATCACGCCACGGATCCTTATTGTC
PvSuS2-TC34561      GAGATGCTTCTCAGGATTAAGCAGCAAGGCCTTGATATCACCCCTAAGATCCTCATTGTT
PvSuS1-TC36733      GAAATGCTTCTGAGGATCAAGCAGTGCGGTCTTGACATCACACCAAAGATCCTTATTGTC
ZmSuSy2_162458267   GAGATGGTTCTCCGTTTAAAGAAACAAGGGCTTGATGTTTCCCCAAAGATTCTCATTGTT
                    ** *** *  *  *  * *** *    ** **  *  *  * **   *** ** *****

SoSUS2_34391403     ACCAGGCTGTTGCCTGATGCTGTTGGGACTACGTGCGGTCAGCGTCTGGAGAAGGTCATT
OsSUS2_16905492     ACCAGGCTGTTGCCTGATGCTGTTGGTACTACATGCGGCCAGCGTGTGGAGAAGGTTATT
OsSUS3_1196836      ACCAGGTTGCTACCTGATGCGCATGGCACCACATGTGGCCAGCGCCTTGAGAAGGTCCTA
ZmSUS1_162460680    ACCAGGTTGCTCCCTGATGCAACTGGCACCACCTGTGGCCAGCGCCTTGAGAAGGTCCTT
BoSUS1_62865492     ACCAGGCTGCTCCCTGATGCAACTGGCACCACTTGTGGTCAGCGTCTTGAGAAGGTCCTT
SbSUS2_222876000    ACCAGGCTGTTGCCTGATGCTGTTGGGACTACGTGCGGTCAGCGTCTGGAGAAGGTCATT
LpSUS_88687740      ACCAGGTTGTTGCCTGATGCTGTTGGAACTACATGTGGCCAGCGGCTGGAGAAGGTCATT
BoSUS4_55741122     ACCAGGCTGTTGCCTGATGCTGTTGGGACTACATGCGGCCAGCGCCTGGAGAAGGTTCTT
BoSUS2_17980242     ACCAGGCTGTTGCCCGATGCTGTTGGGACTACATGTGGCCAGCGTCTGGAGAAGGTTATT
BoSUS3_17980240     ACCAGGTTGCTCCCTGATGCAACTGGCACCACCTGTGGTCAGCGTCTTGAGAAGGTCCTT
PvSuS2-TC34561      ACCAGGCTGTTGCCTGATGCTGTTGGTACTACTTGTGGCCAGCGGCTAGAGAAGGTCATT
PvSuS1-TC36733      ACCAGGTTGCTCCCTGATGCAACTGGCACCACCTGTGGCCAGCGTCTCGAGAAGGTCCTT
ZmSuSy2_162458267   ACTCGGCTGATACCAGATGCAAAAGGAACATCATGCAATCAGCGGCTTGAGAGAATTAGT
                    **  ** ** * ** *****    ** **  * **    *****  * ****   *

SoSUS2_34391403     GGAACCGAGCACACAGACATTATTCGTATTCCATTCAGAAATGAGAATGGTATTCTCCGC
OsSUS2_16905492     GGAACTGAGCACACTGACATTCTTCGTGTTCCATTCAGGAGTGAGAATGGTATCCTCCGC
OsSUS3_1196836      GGCACTGAGCACACTCATATCCTGCGTGTGCCATTCCGAACAGAAAATGGGACTGTTCGC
ZmSUS1_162460680    GGCACCGAGCACTGCCATATCCTTCGCGTGCCATTCAGAACAGAAAACGGAATCGTTCGC
BoSUS1_62865492     GGCACTGAACACACGCACATCCTTCGTGTGCCATTCAGAACTGAAAATGGAATCGTTCGC
SbSUS2_222876000    GGAACTGAGCACACTGACATTATTCGTATTCCATTCAGAAATGAGAATGGTATTCTCCGC
LpSUS_88687740      GGAACTGAGCACACTGACATTCTCCGCGTTCCCTTTAGAACCGAGAATGGGA---TCCGT
BoSUS4_55741122     GGAACTGAGCACACAGACATTCTCCGTGTACCATTCAGAACTGAGAATGGGATCCTCCGT
BoSUS2_17980242     GGAACTGAGCACACAGACATTCTCCGTGTTCCATTCAGAACTGAGAATGGGATCCTCCGC
BoSUS3_17980240     GGCACCGAGCACACCCACATCCTTCGTGTGCCATTCAGAACTGAAAATGGAATTGTTCGC
PvSuS2-TC34561      GGAACTGAGCACACAGACATTATTCGTGTTCCATTCAGAAATGAGAATGGTATTCTTCGC
PvSuS1-TC36733      GGCACTGAGCACTGCCATATCCTTCGTGTGCCATTCAGAACTGAGAATGGAATTGTTCGC
ZmSuSy2_162458267   GGAACACAACATACTTACATATTACGAGTTCCCTTCAGAAATGAAAATGGGATACTTAAG
                    ** **  * **     * **  * **  * ** **  *  *  ** ** ** *   *

SoSUS2_34391403     AAGTGGATCTCTCGTTTTGATGTCTGGCCATACCTGGAGACATACACTGAGGATGTTGCC
OsSUS2_16905492     AAGTGGATCTCCCGTTTTGATGTCTGGCCATTCCTGGAAACATACACTGAGGATGTTGCA
```

```
OsSUS3_1196836      AAATGGATCTCGCGTTTTGAAGTCTGGCCTTACCTGGAAACTTACACCGATGATGTGGCA
ZmSUS1_162460680    AAGTGGATCTCGCGATTTGAAGTCTGGCCGTACCTGGAGACTTACACTGATGACGTGGCG
BoSUS1_62865492     AAATGGATCTCACGTTTTGAAGTCTGGCCGTACCTGGAGACTTTCACTGATGATGTGGCA
SbSUS2_222876000    AAGTGGATCTCTCGTTTTGATGTCTGGCCATACCTGGAGACATACACTGAGGATGTTGCC
LpSUS_88687740      AAGTGGATCTCGCGTTTCGATGTCTGGCAATACCTGGAGACATACACCGAGGATGTTGCA
BoSUS4_55741122     AAGTGGATCTCTCGTTTTGATGTCTGGCCATTCCTGGAGACATACACTGAGGATGTTGCG
BoSUS2_17980242     AAGTGGATCTCTCGTTTTGATGTCTGGCCATTCCTGGAGACATACACTGAGGATGTTGCA
BoSUS3_17980240     AAATGGATCTCACGTTTTGAAGTCTGGCCGTACCTGGAGACTTTCACTGATGATGTGGCA
PvSuS2-TC34561      AAGTGGATCTCTCGTTTTGATGTCTGGCCATACCTAGAGACATACACTGAGGATGTTGCC
PvSuS1-TC36733      AAGTGGATCTCGCGTTTTGAAGTCTGGCCATACCTGGAGACTTACACCGATGACGTGGCA
ZmSuSy2_162458267   AAATGGATATCAAGATTTGATGTGTGGCCATATCTGGAAACATTTGCTGAGGATGCTGCT
                    ** ***** **  * ** ** ** ****  *  ** ** ** *   * ** ** *  **

SoSUS2_34391403     AGTGAAATAATGTTAGAAATGCAGGCCAAGCCTGACCTTATTGTTGGCAACTACAGTGAT
OsSUS2_16905492     AACGAAATTATGAGGGAAATGCAAGCCAAACCTGATCTCATCATTGGCAATTACAGTGAT
OsSUS3_1196836      CACGAGATTTCTGGAGAGCTGCAGGCCACCCCTGACCTGATCATTGGGAACTACAGTGAT
ZmSUS1_162460680    CATGAGATTGCTGGAGAGCTTCAGGCCAATCCTGACCTGATCATCGGAAACTACAGTGAC
BoSUS1_62865492     CACGAGATTGCTGGAGAGCTCCAGGCCAACCCTGACCTGATCATCGGAAACTACAGTGAT
SbSUS2_222876000    AGTGAAATAATGTTAGAAATGCAGGCCAAGCCTGACCTTATCGTTGGCAACTACAGTGAT
LpSUS_88687740      AACGAACTCATGAGGGAAATGCAGACCAAGCCTGATTTGATCATTGGCAACTACAGTGAT
BoSUS4_55741122     AACGAAATCATGCGAGAAATGCAGGCCAAGCCTGATCTCATCATTGGTAACTACAGTGAC
BoSUS2_17980242     AACGAGATTATGAGAGAAATGCAGGCCAAGCCTGATCTCATCATTGGCAATTACAGTGAC
BoSUS3_17980240     CACGAGATTGCTGGAGAGCTCCAAGCCAATCCCGACCTGATCATCGGGAACTACAGTGAT
PvSuS2-TC34561      AGTGAAATCATGAAAGAAATGCAGGCCAAGCCTGACCTTATCATTGGCAACTACAGTGAT
PvSuS1-TC36733      CACGAGATTGCTGGAGAGCTTCAGGCCAATCCTGACCTGATCATCGGTAACTACAGTGAT
ZmSuSy2_162458267   GGTGAAATTGCTGCTGAATTACAAGGTACTCCAGACTTCATAATTGGAAACTACAGTGAT
                       **  *       **  * **    *  ** **  * **  * ** ** *********

SoSUS2_34391403     GGCAATCTAGTCGCCACTCTGCTCGCGCACAAGTTGGGAGTTACTCAGTGTACCATTGCC
OsSUS2_16905492     GGAAACCTTGTTGCCACTCTGCTGGCTCACAAATTAGGAGTTACCCAGTGTACCATTGCT
OsSUS3_1196836      GGCAACCTTGTTCGATGTTTGCTGGCACACAAGTTGGGTGTCACTCATTGTACAATCGCC
ZmSUS1_162460680    GGAAACCTTGTTGCGTGTTTGCTCGCCCACAAGATGGGTGTTACTCACTGTACCATTGCC
BoSUS1_62865492     GGAAACCTTGTTGCGTGCTTGCTTGCACACAAGATGGGTGTTACTCATTGTACCATCGCC
SbSUS2_222876000    GGCAACCTAGTCGCCACTCTGCTCGCACACAAGTTGGGAGTTACTCAGTGTACCATTGCC
LpSUS_88687740      GGTAACCTTGTGGCCACTCTGCTTGCCCATAAATTGGGTGTTACCCAGTGCACCATTGCC
BoSUS4_55741122     GGTAACCTTGTTGCCACTCTGCTTGCGCACAAACTGGGAGTTACTCAGTGTACCATTGCC
BoSUS2_17980242     GGCAACCTTGTTGCCACTCTGCTTGCGCACAAATTGGGAGTTACTCAGTGTACCATCGCC
BoSUS3_17980240     GGAAACCTTGTTGCATGCTTGCTTGCACACAAGATGGGTGTTACTCATTGTACCATTGCC
PvSuS2-TC34561      GGCAACCTAGTTGCCACTCTGCTTGCGCACAAGCTGGGAGTTACTCAGTGTACCATTGCC
PvSuS1-TC36733      GGAAACCTTGTTGCATGTTTGCTTGCGCACAAGATGGGTGTTACTCACTGTACCATTGCC
ZmSuSy2_162458267   GGAAATCTTGTGGCGTCATTGCTATCTTACAAGATGGGAATTACCCAGTGCAACATTGCT
                    ** ** ** **        ****  *  * **  * **  * ** ** ** *  ** **

SoSUS2_34391403     CACGCCTTGGAGAAAACCAAATATCCCAACTCAGACATATACTTAGACAAATTTGACAGC
OsSUS2_16905492     CATGCCTTGGAGAAAACCAAATACCCCAACTCAGACATATACTTGGACAAGTTTGACAGC
OsSUS3_1196836      CATGCACTTGAGAAAACCAAGTACCCCAACTCCGACCTTTACTGGAAGAAGTTTGAGGAT
ZmSUS1_162460680    CATGCGCTTGAGAAAACTAAGTACCCTAACTCCGACCTCTACTGGAAGAAGTTTGAGGAT
BoSUS1_62865492     CATGCGCTTGAGAAAACCAAGTACCCCAATTCCGACCTCTACTGGAAGAAGTTTGAGGAC
SbSUS2_222876000    CATGCCTTGGAGAAAACCAAATACCCCAACTCGGACATATACTTGGACAAATTTGACAGC
LpSUS_88687740      CATGCCTTGGAGAAAACCAAGTACCCCAACTCAGACATATACTTGGACAAATTCGACAGC
BoSUS4_55741122     CACGCCTTGGAGAAAACAAAATACCCCAACTCGGACATATACTTGGACAAATTTGACAGT
BoSUS2_17980242     CACGCCTTGGAGAAAACCAAATACCCCAACTCAGACATATACTTGGACAAATTCGACAGC
BoSUS3_17980240     CATGCGCTTGAGAAAACCAAGTACCCCAACTCCGACCTCTACTGGAAGAAGTTTGAGGAC
PvSuS2-TC34561      CACGCCTTGGAGAAAACCAAATACCCTAACTCAGACATATACTTGGACAAATTCGACAGT
PvSuS1-TC36733      CACGCTCTTGAGAAAACTAAGTACCCCAACTCTGACCTCTACTGGAAGAAGTTTGAGGAC
ZmSuSy2_162458267   CATGCTCTGGAAAAGACTAAGTATCCAGATTCAGACATATTTTGGAAGAATTTCGATGAG
                    ** **  * ** ** ** ** ** **  * ** *** * *  *   * ** ** **
```

```
SoSUS2_34391403      CAATACCACTTCTCATGCCAGTTCACAGCTGACCTTATTGCCATGAATCACACTGATTTC
OsSUS2_16905492      CAGTACCACTTCTCATGCCAATTCACTGCTGATCTTATCGCCATGAATCACACTGATTTC
OsSUS3_1196836       CACTATCACTTCTCCTGCCAGTTCACAGCTGACCTGATTGCAATGAACCATGCTGACTTC
ZmSUS1_162460680     CACTACCACTTCTCGTGCCAGTTCACCACTGACTTGATTGCAATGAACCATGCCGACTTC
BoSUS1_62865492      CACTACCACTTCTCATGCCAGTTTACTACTGACTTGATTGCCATGAACCACGCTGACTTC
SbSUS2_222876000     CAATACCACTTCTCATGCCAGTTCACAGCTGACCTTATTGCCATGAATCACACTGATTTC
LpSUS_88687740       CAGTATCACTTTTCATGCCAGTTCACAGCTGACCTGATTGCCATGAACCACACTGATTTC
BoSUS4_55741122      CAGTACCACTTCTCATGCCAGTTCACAGCAGACCTTATTGCCATGAATCACACTGATTTC
BoSUS2_17980242      CAGTACCACTTCTCATGCCAGTTCACAGCGGACCTTATTGCTATGAATCACACTGATTTC
BoSUS3_17980240      CACTACCACTTCTCATGCCAGTTCACTACTGACTTGATCGCTATGAACCACGCCGACTTC
PvSuS2-TC34561       CAGTATCACTTCTCATGCCAGTTCACAGCTGACCTTATTGCTATGAATCATACCGATTTC
PvSuS1-TC36733       CACTACCACTTCTCGTGCCAGTTCACCACTGACTTGATCGCTATGAACCACGCTGACTTC
ZmSuSy2_162458267    AAGTACCATTTCTCCTGCCAGTTCACTGCTGATATAATTGCTATGAACAATGCTGATTTT
                      * ** ** ** ** ***** ** **  * **  * ** ** *****  *  * ** **

SoSUS2_34391403      ATCATCACCAGTACATTCCAAGAAATCGCGGGAAGCAAGGACACTGTGGGGCAGTATGAG
OsSUS2_16905492      ATCATCACCAGTACATTCCAAGAAATTGCTGGAAGCAAGGACACTGTGGGGCAGTATGAA
OsSUS3_1196836       ATCATCACAAGTACCTTCCAGGAGATTGCTGGAAACAAGGAAACTGTGGGGCAGTATGAG
ZmSUS1_162460680     ATCATCACCAGTACCTTCCAAGAGATCGCCGGAAACAAGGACACCGTCGGCCAGTACGAG
BoSUS1_62865492      ATCATCACCAGTACCTTCCAAGAGATTGCCGGAAACAAGGACACCGTTGGTCAGTACGAG
SbSUS2_222876000     ATCATCACCAGTACATTCCAAGAAATCGCAGGGAGCAAGGACACTGTGGGGCAGTATGAG
LpSUS_88687740       ATCATTACCAGCACATTCCAGGAAATTGCTGGAAGCAAGGATAGCGTGGGTCAATATGAG
BoSUS4_55741122      ATCATCACCAGTACATTCCAAGAAATCGCTGGAAGCAAGGACACCGTGGGCCAATATGAG
BoSUS2_17980242      ATCATCACCAGTACATTCCAAGAAATCGCTGGAAGCAAGGATACTGTGGGGCAATATGAG
BoSUS3_17980240      ATCATCACCAGTACCTTCCAAGAGATTGCCGGAAACAAGGACACCGTTGGTCAGTACGAG
PvSuS2-TC34561       ATCATCACAAGTACATTCCAAGAAATCGCTGGAAGCAAGGACACTGTTGGGCAATATGAG
PvSuS1-TC36733       ATCATCACCAGTACCTTCCAAGAGATTGCTGGAAACAAGGACACCGTCGGTCAGTACGAG
ZmSuSy2_162458267    ATCATCACCAGCACATACCAAGAAATTGCTGGAAGCAAAAATACTGTTGGACAGTATGAG
                     ***** ** ** ** * *** ** ** ** ** * ***  * *  ** ** ** ** **

SoSUS2_34391403      TCCCACATTGCGTTCACTCTTCCTGGACTTTACCGTGTTGTCCATGGCATTGATGTTTTT
OsSUS2_16905492      TCACACATTGCATTCACCCTTCCTGGGCTTTACCGAGTTGTGCATGGCATAGATGTTTTT
OsSUS3_1196836       TCTCACATGGCATTCACAATGCCTGGCCTTTATCGTGTTGTCCATGGTATCGATGTCTTT
ZmSUS1_162460680     TCACACATGGCGTTCACAATGCCTGGCCTGTACCGCGTTGTCCACGGCATTGATGTGTTC
BoSUS1_62865492      TCTCACATGGCATTCACAATGCCTGGCCTGTACCGTGTTGTCCATGGTATCGATGTTTTT
SbSUS2_222876000     TCCCACATCGCGTTCACTCTTCCTGGACTTTACCGTGTTGTCCATGGCATTGATGTTTTT
LpSUS_88687740       TCTCACATTGCTTTCACCCTCCCTGATCTGTACCGGGTTGTCCATGGGATTGACGTGTTT
BoSUS4_55741122      TCCCACATTGCGTTCACTCTTTCTGGGCTCTACCGGGTTGTCCATGGCATTGATGTGTTT
BoSUS2_17980242      TCCCACATCGCCTTCACCCTTCCCGGGCTCTACCGGGTTGTCCATGGCATTGATGTGTTT
BoSUS3_17980240      TCTCACATGGCATTCACAATGCCTGGCCTGTACCGTGTTGTCCACGGTATCGATGTTTTT
PvSuS2-TC34561       TCCCACATTGCATTTACACTTCCTGGGCTCTACCGTGTTGTCCATGGCATTGATGTTTTT
PvSuS1-TC36733       TCACACATGGCATTCACAATGCCTGGCCTCTACCGTGTCGTCCATGGTATCGATGTGTTT
ZmSuSy2_162458267    AGTCATACTGCCTTTACTCTGCCTGGTCTGTACCGAGTTGTCCATGGGATCGATGTCTTC
                        ** *  ** ** **  *  * *  ** ** ** ** ** ** ** ** ** ** **

SoSUS2_34391403      GATCCCAAATTCGACATTGTCTCTCCTGGAGCAGACATGAGTGTTTACTACCCATACACT
OsSUS2_16905492      GATCCCAAGTTCAACATTGTCTCTCCTGGAGCTGACATGAGTGTCTACTTCCCGTACACC
OsSUS3_1196836       GACCCCAAGTTCAACATCGTCTCTCCTGGTGCTGACATGTCCATCTACTTCCCATTCACC
ZmSUS1_162460680     GACCCCAAGTTCAACATCGTGTCTCCTGGCGCGGACCTGTCCATCTACTTCCCGTACACC
BoSUS1_62865492      GACCCCAAGTTTAACATTGTCTCACCTGGTGCGGACCTGTCCATCTACTTCCCTTACACC
SbSUS2_222876000     GATCCCAAATTCAACATTGTCTCTCCTGGAGCAGACATGAGTGTTTACTACCCATACACT
LpSUS_88687740       GATCCTAAGTTCAACATTGTCTCTCCTGGAGCAGACATGACTGTCTACTTCCCATATACC
BoSUS4_55741122      GATCCTAAGTTCAACATTGTCTCTCCTGGAGCAGACATGAGTGTCTACTTCCCATACACC
BoSUS2_17980242      GATCCTAAGTTCAACATTGTCTCTCCTGGAGCAGACATGAGCGTCTACTTCCCATACACC
BoSUS3_17980240      GACCCCAAGTTTAACATTGTCTCACCTGGTGCGGACATGTCCATCTACTTCCCTTACTCC
PvSuS2-TC34561       GATCCCAAGTTCAATATCGTCTCTCCTGGAGCAGACATGAGTGTGTACTACCCATACACT
```

```
PvSuS1-TC36733      GACCCTAAGTTCAACATTGTCTCCCCTGGTGCGGACATGTCCATCTACTTCCCTTACACT
ZmSuSy2_162458267   GATCCAAAGTTCAATATAGTCTCTCCTGGAGCTGACATGTCCATATACTTTCCACATACC
                    ** ** ** **  * ** ** ** ***** ** *** **    * ****  **     *

SoSUS2_34391403     GAAACTGACAAGAGACTCACTGCCTTCCATCCTGAAATTGAGGAGCTCATCTACAGTGAT
OsSUS2_16905492     GAGGCTGACAAGAGGCTCACTGCTTTCCACCCTGAAATTGAGGAGCTTCTCTACAGTGAA
OsSUS3_1196836      GAATCACAGAAGAGGCTCACCTCTCTCCATTTAGAGATAGAGGAGCTACTCTTCAGTGAT
ZmSUS1_162460680    GAGTCGCACAAGAGGCTGACCTCCCTTCACCCGGAGATTGAGGAGCTCCTGTACAGCCAA
BoSUS1_62865492     GAATCACACAAGAGGCTCACCTCCCTCCACCCAGAGATTGAGGAGTTGCTCTACAGTGAT
SbSUS2_222876000    GAAACTGACAAGAGACTCACTGCCTTCCATCCTGAAATTGAGGAGCTCATCTACAGTGAT
LpSUS_88687740      GAGACTGACAAGAGGCTCACCGCCTTCCACTCTGAAATTGAGGAGCTCCTGTACAGCGAT
BoSUS4_55741122     GAGACTGACAAGAGGCTCACTGCCTTCCACCCTGAAATTGAAGAGCTCATTTACAGTGAT
BoSUS2_17980242     GAGACTGACAAGAGGCTCACTGCCTTCCACCCTGAAATTGAGGAGCTCATTTACAGCGAT
BoSUS3_17980240     GAGTCACACAAGAGGCTCACCTCCCTCCACCCAGAGATTGAGGAGTTGCTCTACAGTGAT
PvSuS2-TC34561      GAAACTGACAAGAGACTCACTGCCTTCCACCCTGAGATTGAGGAGCTCATTTACAGTGAT
PvSuS1-TC36733      GAGTCACACAAGAGGCTGACCTCCCTCCACCCTGAGATCGAGGAGCTCCTCTACAGCCAA
ZmSuSy2_162458267   GAGAAGGCCAAGCGACTCACCTCTCTTCATGGTTCAATCGAAAATTTGATTTATGACCCG
                    **       *** * ** **  *  * **       ** **  *  *  * *

SoSUS2_34391403     GTTGAGAATGATGAGCACAAGTTTGTGTTGAAGGACAAGAACAAGCCGATCATCTTCTCA
OsSUS2_16905492     GTCGAGAACGATGAACACAAGTTTGTATTGAAGGACAAGAACAAGCCAATCATCTTCTCC
OsSUS3_1196836      GTTGAAAACACTGAGCACAAGTTTGTTCTGAAGGACAAGAAGAAGCCAATCATCTTCTCG
ZmSUS1_162460680    ACCGAGAACACGGAGCACAAGTTCGTTCTGAACGACAGGAACAAGCCAATCATCTTCTCC
BoSUS1_62865492     GTTGACAACCATGAGCACAAGTTTGTGCTGAAGGACAGGAACAAGCCAATCATCTTCTCG
SbSUS2_222876000    GTTGAGAACGATGAGCACAAGTTTGTGTTGAAGGACAAGAACAAGCCGATCATCTTCTCA
LpSUS_88687740      GTTGAGAACGATGAACACAAATTTGTGAAGAAGGACAGGAACAAGCCAATCATCTTTTCA
BoSUS4_55741122     GTCGAGAACTCTGAACACCAGTTTGTATTGAAGGACAAGAACAAGCCGATCATCTTCTCA
BoSUS2_17980242     GTCGAGAACTCTGAACACAAGTTTGTATTGAAGGACAAGAACAAGCCGATCATATTCTCA
BoSUS3_17980240     GTTGACAACAATGAGCACAAGTTTGTGCTAAAGGACAGGAACAAGCCAATCATCTTCTCG
PvSuS2-TC34561      GTTGAGAACTCGGAGCACAAGTTTGTCTTGAAGGACAAGAACAAGCCGATCATTTTCTCA
PvSuS1-TC36733      ACCGAGAACAACGAGCACAAGTTCGTGCTGAACGACAGGAACAAGCCCATCATCTTCTCC
ZmSuSy2_162458267   GAGCAAAACGATGAACACATTGGGCATCTGGATGACCGGTCAAAGCCCATCCTCTTCTCC
                        * **    ** ***             * ***  *   ***** *** * ** **

SoSUS2_34391403     ATGGCTCGTCTTGACCGTGTGAAGAACATGACAGGCTTGGTTGAGATGTATGGTAAGAAT
OsSUS2_16905492     ATGGCTCGTCTTGACCGAGTGAAGAACATGACAGGTCTGGTTGAGATGTATGGTAAGAAT
OsSUS3_1196836      ATGGCTAGGCTAGACCATGTCAAGAATTTGACTGGTCTGGTTGAGTTGTATGGTCGGAAC
ZmSUS1_162460680    ATGGCTCGTCTCGACCGTGTGAAGAACTTGACTGGGCTGGTGGAGCTGTACGGCCGGAAC
BoSUS1_62865492     ATGGCTCGTCTTGACCGTGTCAAGAACTTGACTGGTCTGGTTGAGCTGTATGGCCGAAAT
SbSUS2_222876000    ATGGCTCGTCTTGACCGTGTGAAGAACATGACAGGCTTGGTTGAGATGTATGGTAAGAAT
LpSUS_88687740      ATGGCTCGTCTTGACCGCGTGAAGAACATGACTGGCTTGGTTGAGATGTACGGAAAGAAC
BoSUS4_55741122     ATGGCTCGTCTTGACCGTGTGAAGAACATGACCGGTTTGGTTGAGATGTATGGCAAGAAT
BoSUS2_17980242     ATGGCTCGCCTCGACCGAGTGAAGAACATGACTGGTTTAGTTGAGATGTACGGTAAGAAT
BoSUS3_17980240     ATGGCTCGTCTCGACCGTGTCAAGAACTTGATTGGTCTGGTTGAGCTGTATGGCCGGAAC
PvSuS2-TC34561      ATGGCTCGTCTTGACCGTGTGAAGAACATGACAGGCTTGGTTGAGATGTACGGTAAGAAT
PvSuS1-TC36733      ATGGCTCGTCTTGACCGTGTCAAGAACTTGACTGGTCTGGTCGAGCTCTACGGCCGGAAC
ZmSuSy2_162458267   ATGGCAAGACTCGACAGGGTGAAGAACATTACAGGGCTGGTCGAAGCTTTTGCTAAGTGC
                    *****  * ** ***   ** *****  * *  **  * ** **    *  *

SoSUS2_34391403     GCACGCCTGAGGGAATTGGCAAACCCTGTGATTGTTGCTGGTGACC---ATGGCAAGGAA
OsSUS2_16905492     GCACATCTCAGGGATTTGGCAAACCTTGTGATTGTTTGTGGTGACC---ACGGCAATCAG
OsSUS3_1196836      CCTCGCCTGCAAGAGCTAGTAAACCTTGTGGTTGTCTGTGGTGACC---ATGGCAAGGAA
ZmSUS1_162460680    AAGCGGCTGCAGGAGCTGGTGAACCTCGTGGTCGTCTGCGGCGACC---ATGGCAACCCT
BoSUS1_62865492     CCTCGCCTGCAAGAGCTGGTTAACCTTGTGGTTGTCTGTGGTGACC---ATGGCAATCCA
SbSUS2_222876000    GCACGCCTGAGGGAATTGGCAAACCTTGTGATTGTTGCTGGTGACC---ATGGCAAGGAA
LpSUS_88687740      GCACATCTGAAGGACTTGGCAAACCTTGTGATTGTTGCTGGTGACC---ATGGCAAGGAG
BoSUS4_55741122     GCACATCTGAGGGATTTGGCAAACCTTGTGGTTGTTGCTGGTGACC---ATGGCAAGGAG
```

```
BoSUS2_17980242     GCACATCTGAGGGATTTGGCAAACCTTGTGATTGTTGCTGGTGACC---ATGGCAAGGAG
BoSUS3_17980240     CCTCGCCTGCAGGAGCTGGTTAACCTTGTGGTTGTCTGTGGTGACC---ATGGCAATCCA
PvSuS2-TC34561      GCGCACCTGAGGGAATTGGCGAACCTTGTGATTGTTGCTGGTGACC---ATGGCAAGGAG
PvSuS1-TC36733      AAGCGTCTGCAGGAGCTGGTCAACCTTGTGGTCGTCTGCGGTGACC---ACGGCAACCCG
ZmSuSy2_162458267   GCTAAGCTGAGGGAGCTGGTAAACCTTGTCGTCGTTGCCGGGTACAATGATGTCAACAAG
                          **    **  * *  ****  **  * **    **  **    * * ***

SoSUS2_34391403     TCGAAGGACAGGGAGGAGCAGGCAGAGTTCAAGAAGATGTACAGTCTCATTGATGAGTAC
OsSUS2_16905492     TCCAAGGACAGGGAGGAGCAGGCTGAGTTCAAGAAGATGTACGGTCTCATTGACCAGTAC
OsSUS3_1196836      TCCAAGGACAAAGAAGAGCAGGCTGAGTTCAAGAAGATGTTTAATCTGATCGAGCAGTAC
ZmSUS1_162460680    TCCAAGGACAAGGAGGAGCAGGCCGAGTTCAAGAAGATGTTTGACCTCATCGAGCAGTAC
BoSUS1_62865492     TCCAAGGACAAGGAGGAGCAGGCTGAGTTCCAGAAGATGTTTGACCTTATCGAGCAATAC
SbSUS2_222876000    TCGAAGGACAGGGAGGAGCAGGCAGAGTTCAAGAAGATGTACAGTCTCATTGATGAGTAC
LpSUS_88687740      TCCAAGGATAGGGAGGAGCAGGCTGAGTTCAAGAGGATGTACAGCCTCATTGAGGAGTAC
BoSUS4_55741122     TCTAAGGACAGGGAGGAGCAGGCTGAGTTCAAGAGGATGTACAGTCTAATTGAGGAGTAC
BoSUS2_17980242     TCCAAGGACAGGGAGGAGCAAGCTGAGTTCAAGAGGATGTACAGTCTAATTGATGAGTAC
BoSUS3_17980240     TCCAAGGACAAGGAGGAGCAGGCTGAGTTCAAGAAGATGTTTGACCATATTGAGCAATAC
PvSuS2-TC34561      TCCAAGGACAGGGAGGAGCAGGCAGAGTTCAAGAAGATGTACAGTCTCATTGACCAGTAC
PvSuS1-TC36733      TCCAAGGACAAGGAGGAGCAGGCCGAGTTCAAGAAGATGTTCGACCTCATCGAGCAGTAC
ZmSuSy2_162458267   TCCAAGGACAGGGAAGAGATCGCGGAGATAGAGAAGATGCATGAACTCATCAAGACCCAC
                    ** ***** *  ** ***   ** *** *  *** ****      *  **  *     **

SoSUS2_34391403     AACTTGAAGGGCCATATCCGGTGGATCTCAGCTCAGATGAACCGTGTCCGCAACGCTGAG
OsSUS2_16905492     AAGTTGAAGGGGCATATCCGCTGGATCTCAGCTCAGATGAACCGTGTTCGTAACGGGGAG
OsSUS3_1196836      AATTTGAATGGCCACATCCGCTGGATCTCCGCTCAGATGAACCGTGTCCGCAATGGTGAG
ZmSUS1_162460680    AACCTGAACGGGCACATCCGCTGGATCTCCGCCCAGATGAACCGCGTCCGCAACGGCGAG
BoSUS1_62865492     AACCTGAACGGCCACATCCGCTGGATCTCTGCTCAGATGAACCGTGTCCGCAATGGTGAG
SbSUS2_222876000    AACTTGAAGGGCCATATTCGGTGGATCTCAGCTCAGATGAACCGTGTCCGCAATGCGGAG
LpSUS_88687740      AAGCTGAAAGGCCATATCCGTTGGATCTCAGCTCAGATGAACCGTGTTCGCAATGGCGAG
BoSUS4_55741122     AAGTTGAAGGGCCATATCCGGTGGATCTCCGCTCAGATGAACCGTGTTCGCAATGGGGAG
BoSUS2_17980242     AAGTTGAAGGGTCATATCCGGTGGATCTCGGCTCAGATGAACCGTGTTCGGAATGGGGAG
BoSUS3_17980240     AACCTGAATGGCCACATCCGCTGGATCTCTGCTCAGATGAACCGTGTCCGCAATGGTGAG
PvSuS2-TC34561      AATTTGAAGGGCCATATCCGGTGGATCTCGGCTCAGATGAACCGTGTCCGCAATGGGGAG
PvSuS1-TC36733      AACCTGAACGGGCACATCCGCTGGATCTCTGCCCAGATGAACCGTGTCCGCAACGGTGAG
ZmSuSy2_162458267   AACTTGTTCGGGCAGTTCCGCTGGATCTCTGCCCAGACAAACAGGGCCCGTAACGGCGAG
                    **  **   ** **  * ** ******** ** ****  *** * *  ** ** *  ***

SoSUS2_34391403     TTGTACCGCTACATTTGTGACACAAAGGGAGCATTTGTGCAGCCTGCATTCTATGAAGCA
OsSUS2_16905492     TTGTACCGATACATTTGTGACACCAAGGGAGTCTTTGTCCAGCCTGCATTCTATGAAGCG
OsSUS3_1196836      CTCTACCGCTACATCTGCGACATGAGGGGAGCCTTTGTGCAGCCCGCTCTCTATGAGGCC
ZmSUS1_162460680    CTGTACCGCTACATCTGCGACACCAAGGGCGCCTTCGTGCAGCCTGCTTTCTACGAGGCT
BoSUS1_62865492     CTCTACCGTTACATCTGCGACACCAAGGGTGCTTTCGTGCAGCCTGCTTTCTACGAGGCT
SbSUS2_222876000    TTGTACCGCTACATTTGTGACACGAAGGGAGCATTTGTGCAGCCTGCATTCTATGAAGCA
LpSUS_88687740      CTGTACCGCTACATCTGCGACACCAAGGGAGCATTTGTGCAGCCTGCATTCTATGAAGCT
BoSUS4_55741122     CTGTACCGCTACATTTGTGACACCAGGGGAGTATTTGTGCAGCCTGCATTCTATGAAGCG
BoSUS2_17980242     TTGTACCGCTACATTTGCGACACCAAGGGAGTATTTGTGCAGCCTGCATTCTACGAAGCG
BoSUS3_17980240     CTCTACCGCTACATTTGCGACACCAGGGGTGCCTTTGTGCAGCCTGCTTTCTACGAGGCT
PvSuS2-TC34561      TTGTACCGCTACATTTGTGACACAAAGGGAGCATTTGTGCAGCCTGCATTCTATGAAGCG
PvSuS1-TC36733      CTCTACCGCTACATCTGCGACACCCAGGGCGCCTTCGTGCAGCCTGCTTTCTACGAGGCC
ZmSuSy2_162458267   CTCTATCGCTACATCGCTGATACCCATGGTGCTTTCGTACAGCCGGCCTTCTATGAAGCG
                     * ** ** *****    ** *     ** *  ** ** ***** **  **** ** **

SoSUS2_34391403     TTCGGCCTGACTGTCATTGAGTCCATGACGTGCGGTTTGCCAACAATTGCAACCTGCCAT
OsSUS2_16905492     TTTGGTCTGACTGTCATCGAAGCCATGACATGTGGTTTGCCAACAATCGCAACATGCCAT
OsSUS3_1196836      TTTGGGCTAACTGTGATTGAGGCCATGACCTGTGGTCTTCCAACATTTGCAACTGCCTAT
ZmSUS1_162460680    TTCGGGCTGACGGTGGTTGAGGCCATGACCTGCGGCCTGCCCACGTTCGCCACCGCCTAC
BoSUS1_62865492     TTCGGGCTTACCGTGGTCGAGTCCATGACCTGCGGCCTTCCGACATTTGCAACTGCCTAT
```

```
SbSUS2_222876000    TTTGGCCTGACTGTCATTGAGTCCATGACGTGCGGTTTGCCAACAATTGCAACCTGCCAT
LpSUS_88687740      TTTGGCCTGACTGTCATTGAGGCCATGACATGTGGTCTGCCGACAATTGCGACATGCCAC
BoSUS4_55741122     TTTGGCCTGACTGTCATTGAGTCCATGACATGCGGTTTGCCAACAATCGCAACATGCCAT
BoSUS2_17980242     TTTGGCCTGACTGTCATTGAGTCCATGACATGCGGTTTGCCAACAATCGCAACATGCCAT
BoSUS3_17980240     TTCGGGCTCACCGTGGTTGAGTCCATGAGCTGCGGTCTCCCGACATTCGCAACCGCCTAC
PvSuS2-TC34561      TTTGGCCTGACTGTCATTGAGTCCATGACATGTGGTTTGCCAACAATTGCGACCTGCCAT
PvSuS1-TC36733      TTCGGTCTGACCGTGGTCGAGGCCATGACCTGCGGCCTGCCCACGTTCGCCACCGCCTAC
ZmSuSy2_162458267   TTCGGTCTCACCGTCGTTGAGGCCATGACCTGTGGGCTTCCTACTTTCGCGACGCTCCAT
                    ** ** ** ** **  * **  ******  ** **  * ** **  * ** **   * *

SoSUS2_34391403     GGTGGCCCTGCTGAAATAATTGTGGACGGGGTGTCTGGTTTGCACATTGATCCTTACCAC
OsSUS2_16905492     GGTGGCCCTGCTGAGATTATTGTTGATGGGGTGTCTGGTCTGCACATTGATCCTTACCAC
OsSUS3_1196836      GGTGGTCCAGCCGAGATCATCGTGCACGGCGTGTCTGGCTACCACATTGATCCTTACCAG
ZmSUS1_162460680    GGCGGTCCGGCCGAGATCATCGTGCACGGCGTGTCTGGCTACCACATCGACCCTTACCAG
BoSUS1_62865492     GGTGGTCCGGCTGAGATCATCGTGCACGGTGTGTCTGGTTTCCACATTGACCCTTACCAG
SbSUS2_222876000    GGTGGCCCTGCTGAAATCATTGTGGACGGGGTGTCTGGTTTACACATTGATCCCTACCAC
LpSUS_88687740      GGTGGCCCTGCTGAGATCATCGTGGATGGAGTGTCTGGTCTGCACATTGATCCTTACCAC
BoSUS4_55741122     GGTGGCCCTGCCGAAATAATTGTCGATGGGGTGTCTGGTTTGCACATTGATCCTTACCAC
BoSUS2_17980242     GGTGGCCCTGCCGAAATCATTGTCGATGGGGTGTCTGGTTTGCACATTGATCCTTACCAC
BoSUS3_17980240     GGTGGTCCGGCTGAGATCATCGTGCACGGTGTGTCAGGCTTCCACATTGATCCTTACCAG
PvSuS2-TC34561      GGTGGCCCTGCTGAAATCATTGTGGACGGG-TGTCTGGTTTGCACATTGACCCTTACACA
PvSuS1-TC36733      GGTGGCCCGGCCGAGATCATCGTGCACGGCGTGTCCGGCTACCACATCGACCCGTACCAG
ZmSuSy2_162458267   GGAGGTCCAGCTGAGATCATAGAGCATGGCGTCTCGGGCTTCCACATTGACCCGTACCAC
                    ** ** ** ** ** ** ** *   * **  * ** **    ***** ** ** ***

SoSUS2_34391403     AGTGACAAGGCTGCAGATATTTTGGTCAACTTCTTTGAGAGGTGCAAGGCAGACCCAAGC
OsSUS2_16905492     AGTGACAAGGCTGCTGATATCTTGGTCAACTTCTTTGAGAAGTGCAAGCAGGATTCAACC
OsSUS3_1196836      AACGACAAGGCCTCGGCGCTGCTCGTGGAGTTCTTTGAGAAGTGTCAGGAAGACCCAAAC
ZmSUS1_162460680    GGCGACAAGGCGTCGGCCCTGCTCGTGGACTTCTTCGACAAGTGCCAGGCGGAGCCGAGC
BoSUS1_62865492     GGCGACAAGGCCTCGGCGCTGCTCGTCGAGTTCTTCGAGAAGTGCCAGCAAGACCACAGC
SbSUS2_222876000    AGTGACAAAGCTGCAGATATCTTGGTCAACTTCTTTGAGAAGTGCAAGGCAGACCCAAGC
LpSUS_88687740      AGCGACAAGGCCGCAGATATCTTGGTCAACTTCTTTGAGAAGAGCACGGCGGATCCAAGC
BoSUS4_55741122     AGTGACAAGGCTGCAGATATCTTGGTCAACTTCTTTGAGAAGTGCAAGGAGGATCCAACC
BoSUS2_17980242     AGTAACAAGGCTGCAGATATCTTGGTCAACTTCTTTGAGAAGTGCAAGGAGGATCCAACC
BoSUS3_17980240     GGTGACAAGGCCTCGGCGCTGCTCGTCGAGTTCTTCGAGAAGTGCCAGCAAGACCCCGCC
PvSuS2-TC34561      GTGA--------------------------------------------------------
PvSuS1-TC36733      GGCGACAAGGCCTCGGCGCTGCTGGTGGACTTCTTCGAGAAGTGCAAGGAGGACTCGAGC
ZmSuSy2_162458267   CCCGAACAGGCTGCTAATCTGATGGCCGACTTCTTCGACCGGTGCAAGCAAGACCCAGAT


SoSUS2_34391403     TACTGGGACAAGATCTCACAGGGTGGACTGCAGAGAATTTATGAGAAGTACACCTGGAAG
OsSUS2_16905492     TACTGGGACAATATTTCACAGGGAGGTCTGCAGAGGATTTACGAGAAGTACACCTGGAAG
OsSUS3_1196836      CACTGGATCAAGATCTCGCAGGGTGGACTTCAGCGCATCGAGGAGAAGTACACATGGAAG
ZmSUS1_162460680    CACTGGAGCAAGATCTCCCAGGGCGGGCTCCAGCGTATCGAGGAGAAGTACACCTGGAAG
BoSUS1_62865492     CACTGGACCAAGATCTCCCAGGGCGGGCTTCAGCGTATTGAGGAGAAGTACACCTGGAAG
SbSUS2_222876000    TACTGGGACAAGATCTCACAGGGTGGACTGCAGAGAATTTATGAGAAGTACACCTGGAAG
LpSUS_88687740      TACTGGGACAAAATCTCCCAGGGAGGCCTGAAGAGAATTTATGAGAAGTACACCTGGAAG
BoSUS4_55741122     TACTGGGACAAGATTTCACTGGGAGGCCTCAAGAGAATTTATGAGAAGTACACCTGGAAG
BoSUS2_17980242     TACTGGGACAAGATTTCACAGGGAGGCCTGAAGAGAATTTATGAGAAGTACACCTGGAAG
BoSUS3_17980240     CACTGGACCAAGATCTCCCAGGGCGGGCTTCAGCGTATTGAGGAGAAATACACCTGGAAG
PvSuS2-TC34561      ------------------------------------------------------------
PvSuS1-TC36733      CACTGGAGCAAGATCTCGCAGGGCGGGCTCCAGCGCATCGAGGAGAAGTACACATGGAAG
ZmSuSy2_162458267   CACTGGGTGAATATATCTGGAGCAGGGCTGCAGCGCATATACGAGAAGTACACATGGAAG


SoSUS2_34391403     CTCTACTCCGAGAGGCTGATGACCCTGACTGGTGTATACGGATTCTGGAAGTATGTGAGC
OsSUS2_16905492     CTGTACTCTGAGAGGCTGATGACCTTGACTGGTGTATACGGATTCTGGAAGTACGTAAGC
```

```
OsSUS3_1196836       CTCTACTCTGAGAGGCTGATGACTCTCTCCGGTGTCTACGGTTTCTGGAAGTATGTCACC
ZmSUS1_162460680     CTGTACTCGGAGAGGCTGATGACCCTCACCGGCGTGTACGGGTTCTGGAAGTACGTGTCC
BoSUS1_62865492      CTCTACTCTGAGAGGCTGATGACCCTCACCGGTGTTTACGGGTTCTGGAAGTACGTCTCC
SbSUS2_222876000     CTCTACTCCGAGAGGCTGATGACCCTGACTGGTGTATACGGATTCTGGAAGTATGTGAGC
LpSUS_88687740       CTCTACTCAGAGAGGCTGATGACCCTGACCGGTGTGTATGGGTTCTGGAAGTACGTGAGC
BoSUS4_55741122      CTGTACTCCGAGAGGCTGATGACCCTGACTGGTGTGTACGGATTCTGGAAGTACGTGAGC
BoSUS2_17980242      CTGTACTCGGAGAGGCTGATGACCCTGACTGGTGTCTATGGATTCTGGAAGTACGTGAGC
BoSUS3_17980240      CTCTACTCTGAGAGGCTGATGACCCTCACTGGTGTTTACGGATTCTGGAAGTACGTCTCC
PvSuS2-TC34561       ------------------------------------------------------------
PvSuS1-TC36733       CTCTACTCGGAGAGGCTGATGACCCTGACCGGCGTGTACGGGTTCTGGAAGTACGTGTCC
ZmSuSy2_162458267    ATATACTCAGAGAGGTTGATGACACTGGCCGGGGTCTACGGTTTCTGGAAGTACGTGTCG

SoSUS2_34391403      AATCTGGAGAGGCGTGAGACTCGCCGCTACCTTGAGATGTTCTATGCTCTGAAATACCGT
OsSUS2_16905492      AACCTTGAGAGGCGCGAGACTCGCCGTTACATTGAGATGTTCTATGCTCTGAAATACCGC
OsSUS3_1196836       AACCTCGACAGGCGTGAGACACGCCGCTACCTGGAGATGCTGTACGCCCTCAAGTACCGC
ZmSUS1_162460680     AACCTGGAGAGGCGCGAGACCCGGCGGTACCTGGAGATGCTGTACGCGCTCAAGTACCGC
BoSUS1_62865492      AACCTCGAGAGGCGCGAGACCCGGCGCTACCTTGAGATGCTGTACGCCCTCAAGTACCGC
SbSUS2_222876000     AACCTGGAGAGGCGTGAGACCCGCCGCTACCTTGAGATGTTCTATGCTTTGAAATACCGT
LpSUS_88687740       AACCTGGAGAGGCGCGAGACTCGCCGTTACCTGGAGATGTTCTACGCTCTCAAGTACCGT
BoSUS4_55741122      AACCTAGAGAGGCGCGAGACTCGGCGCTACCTCGAGATGTTCTACGCTCTGAAATACCGC
BoSUS2_17980242      AACCTAGAGAGGCGTGAGACTCGCCGTTACCTCGAGATGTTCTACGCTCTGAAATACCGT
BoSUS3_17980240      AACCTCGAGAGGCGTGAGACCCGCCGCTACCTTGAGATGCTGTACGCCCTCAAGTACCGC
PvSuS2-TC34561       ------------------------------------------------------------
PvSuS1-TC36733       AACCTGGAGAGGCGCGAGACCCGGCGGTACCTGGAGATGCTGTACGCGCTCAAGTACCGC
ZmSuSy2_162458267    AAGCTCGAGAGGCTGGAGACGAGGCGCTACCTTGAGATGTTCTACATACTGAAGTTCCGC

SoSUS2_34391403      AGCCTGGCAAGTGCGGTTCCATTGTCCTTCGATTAG------------------------
OsSUS2_16905492      AGCCTGGCCAGCGCCGTCCCATTGGCTGTCGATGGAGAGAGCACATCCAAGTAA------
OsSUS3_1196836       AAGATGGCTACCACCGTTCCATTGGCCATTGAGGGAGAGGCCTCCACCAAATGA------
ZmSUS1_162460680     ACCATGGCGAGCACCGTGCCGCTGGCCGTGGAGGGAGAGCCCTCCAGCAAGTGA------
BoSUS1_62865492      ACGATGGCTAGCACTGTTCCATTGGCTGTTGATGGGGAGCCCTCGAGCAAATGA------
SbSUS2_222876000     AGCCTGGCAAGCGCGGTTCCCTTGTCCTACGACTAG------------------------
LpSUS_88687740       AGCCTGGCTGCTGCAGTTCCATTGGCGGTCGCCGGCGAGAGCAGCGGCAACTTGCGCGGA
BoSUS4_55741122      AGCCTGGCAAGCGCCGTCCCATTGGCCGTCGACGGCGACAGCGTAGCCAAGTAG------
BoSUS2_17980242      AGCCTGGCCAGCGCCGTTCCATTGGCCGTCGACGGCGACAGCGCTGCCAATTAG------
BoSUS3_17980240      AAGATGGCTAGCACCGTTCCATTGGCTGTTGAAGGAGAGCCCTCGAACAAATGA------
PvSuS2-TC34561       ------------------------------------------------------------
PvSuS1-TC36733       ACCATGGCCAGCACGGTGCCGCTGGCCGTGGAGGGAGAGCCCTCCAGCAAGTGA------
ZmSuSy2_162458267    GAGCTGGCGAAGACCGTGCCGCTTGCAATTGACCAACCGCAGTAG---------------

SoSUS2_34391403      ------------------------------------------------------------
OsSUS2_16905492      ------------------------------------------------------------
OsSUS3_1196836       ------------------------------------------------------------
ZmSUS1_162460680     ------------------------------------------------------------
BoSUS1_62865492      ------------------------------------------------------------
SbSUS2_222876000     ------------------------------------------------------------
LpSUS_88687740       TTTGGGGCATGCAGAGGGCGCATTCATCGAGCAGGAGGGAGAAGTGTCGGCTGCGCTATG
BoSUS4_55741122      ------------------------------------------------------------
BoSUS2_17980242      ------------------------------------------------------------
BoSUS3_17980240      ------------------------------------------------------------
PvSuS2-TC34561       ------------------------------------------------------------
PvSuS1-TC36733       ------------------------------------------------------------
ZmSuSy2_162458267    ------------------------------------------------------------
```

```
SoSUS2_34391403     ------------------------------------------------------------
OsSUS2_16905492     ------------------------------------------------------------
OsSUS3_1196836      ------------------------------------------------------------
ZmSUS1_162460680    ------------------------------------------------------------
BoSUS1_62865492     ------------------------------------------------------------
SbSUS2_222876000    ------------------------------------------------------------
LpSUS_88687740      ATTTGTCTACCGTCGTTTCCATTTGGTCTGTCTGTCGTGGGGTGTGCGATTTTATGTCTC
BoSUS4_55741122     ------------------------------------------------------------
BoSUS2_17980242     ------------------------------------------------------------
BoSUS3_17980240     ------------------------------------------------------------
PvSuS2-TC34561      ------------------------------------------------------------
PvSuS1-TC36733      ------------------------------------------------------------
ZmSuSy2_162458267   ------------------------------------------------------------


SoSUS2_34391403     ------------------------------------------------------------
OsSUS2_16905492     ------------------------------------------------------------
OsSUS3_1196836      ------------------------------------------------------------
ZmSUS1_162460680    ------------------------------------------------------------
BoSUS1_62865492     ------------------------------------------------------------
SbSUS2_222876000    ------------------------------------------------------------
LpSUS_88687740      GGCACATTCGTGAGGTCTTGGGCAGTGCTGGTTGCTGGTTCATGCCAGCCGCCTCGAATA
BoSUS4_55741122     ------------------------------------------------------------
BoSUS2_17980242     ------------------------------------------------------------
BoSUS3_17980240     ------------------------------------------------------------
PvSuS2-TC34561      ------------------------------------------------------------
PvSuS1-TC36733      ------------------------------------------------------------
ZmSuSy2_162458267   ------------------------------------------------------------


SoSUS2_34391403     ------------------------------------------------
OsSUS2_16905492     ------------------------------------------------
OsSUS3_1196836      ------------------------------------------------
ZmSUS1_162460680    ------------------------------------------------
BoSUS1_62865492     ------------------------------------------------
SbSUS2_222876000    ------------------------------------------------
LpSUS_88687740      AACGCCTGCCCGTGGTGTTCATCTTTCATCAGGGAGTTCAATGCATAA
BoSUS4_55741122     ------------------------------------------------
BoSUS2_17980242     ------------------------------------------------
BoSUS3_17980240     ------------------------------------------------
PvSuS2-TC34561      ------------------------------------------------
PvSuS1-TC36733      ------------------------------------------------
ZmSuSy2_162458267   ------------------------------------------------
```

8.2)Submitted multiple sequence alignment of Amino acid sequences of the gene family:

```
SoSUS2      MAAKLT-----RLHSLRERLGATFSSHPNELIALFSRYVNQGKGMLQRHQLLAEFDALF-
OsSUS2      MAAKLA-----RLHSLRERLGATFSSHPNELIALFSRYVNQGKGMLQRHQLLAEFDALI-
OsSUS3      MGETTGERALNRLHSMRERIGDSLSAHTNELVAVFSRLVNQGKGMLQPHQIIAEYNAAIP
ZmSUS1      MGEGAGDRVLSRLHSVRERIGDSLSAHPNELVAVFTRLKNLGKGMLQPHQIIAEYNNAIP
BoSUS1      MGEAAGDRVLSRLHSVRERIGDSLSAHPNELVAVFTRLVNLGKGMLQPHQIIAEYNNAIP
SbSUS2      MAAKLT-----RLHSLRERLGATFSSHPNELIALFSRYVNQGKGMLQRHQLLAEFDALF-
LpSUS       MAAKLT-----RLHSLRERLGATFSSHPNELIALFSRYVRQGKGMLQRHQLLVEFDALF-
BoSUS4      MAAKLT-----RLHSLRERLGATFSSHPNELIALFSRYVNQGKGMLQRHQLLAEFDALM-
BoSUS2      MAAKLT-----RLHSLRERLSATFSSHPNELIALFSRYVHQGKGMLQRHQLLAEFDALI-
BoSUS3      MGETAGDRVLSRLHSVRERIGDSLSAHPNELVAVFTRLVNLGKGMLQPHQIIAEYNNAIP
```

```
PvSuS1      MGEAAGDRVLSRLHSVRERIGDSLSAHPNELVAVFTRLKNLGKGMLQPHQIIAEYNSAIP
PvSuS2      MAAKLT-----RLHSLRERLGATFSSHPNELIALFSRYVNQGKGMLQRHQLLAEFDALF-
            *.         ****:***:. ::*:*.***:*:*:*  . ****** **::.*::   :

SoSUS2      --DSDKEKYAPFEDFLRAAQEAIVLPPWIALAIRPRPGVWDYIRVNVSELAVEELSVSEY
OsSUS2      --EADKEKYAPFEDILRAAQEAIVLPPWVALAIRPRPGVWDYIRVNVSELAVEELSVSEY
OsSUS3      EGEREKLKDSALEDVLRGAQEAIVIPPWIALAIRPRPGVWEYLRINVSQLGVEELSVPEY
ZmSUS1      EAEREKLKDGAFEDVLRAAQEAIVIPPWVALAIRPRPGVWEYVRVNVSELAVEELRVPEY
BoSUS1      EAEREKLKDGAFEDVLRAAQEAIVIPPWVALAIRPRPGVWEYVRVNVSELAVEELRVPEY
SbSUS2      --DSDKEKYAPFEDFLRAAQEAIVLPPWVALAIRPRPGVWDYIRVNVSELAVEELSVSEY
LpSUS       --ESDKEKYAPFEDILRAAQEAIVLPPWVALAIRPRTGVWDYIRVNVSDLAVEELTVSEY
BoSUS4      --DADKEKYAPFEDILRAAQEAIVLPPWVALAIRPRPGVWDYIRVNVSELAVEELSVSEY
BoSUS2      --AADKEKYAPFEDILRAAQEAIVLPPWVALAIRPRPGVWDYIRVNVSELAVEELSVSEY
BoSUS3      EAERDKLKDGAFEDVLRAAQEAIVIPPWVALAIRPRPGVWEYVRVNVSELAVEELRVPEY
PvSuS1      EAEREKLKDGAFEDVLRAAQEAIVIPPWVALAIRPRPGVWEYVRVNVSELAVEELRVPEY
PvSuS2      --DSDKEKYAPFEDFLRAAQEAIVLPPWVALAIRPRPGVWDYIRVNVSELAVEELSVSEY
                :* * ..:**.**.******:***:*******.***:*:*:***:*.**** *.**

SoSUS2      LAFKEQLVDGNSNSNFVLELDFEPFNASFPRPSMSKSIGNGVQFLNRHLSSKLFQDKESL
OsSUS2      LAFKEQLVDGHTNSNFVLELDFEPFNASFPRPSMSKSIGNGVQFLNRHLSSKLFQDKESL
OsSUS3      LQFKEQLVDGSTQNNFVLELDFEPFNASFPRPSLSKSIGNGVQFLNRHLSSKLFHDKESM
ZmSUS1      LQFKEQLVEEGPNNNFVLELDFEPFNASFPRPSLSKSIGNGVQFLNRHLSSKLFHDKESM
BoSUS1      LQFKEQLVEGSTNNNFVLELDLEPFNASFPRPSLSKSIGNGVQFLNRHLSSKLFHDKESM
SbSUS2      LAFKEQLVDGNSNSNFVLELDFEPFNASFPRPSMSKSIGNGVQFLNRHLSSKLFQDKESL
LpSUS       LAFKEQLVEEHASRKFVLELDFEPFNASAPRPSMSKSYGKGVQFLNRHSSSKLFQDKESL
BoSUS4      LAFKEQLVDGHTNSNFVLELDFEPFNASFPRPSMSKSIGNGVQFLNRHLSSKLFQDKESL
BoSUS2      LEFKEQLVDGHTNSNFVLELDFEPFNASFPRPSMSKSIGNGVQFLNRHLSSKLFQDKESL
BoSUS3      LQFKEQLVEGSTNNNFVLELDFEPFNASFPRPSLSKSIGNGVQFLNRHLSSKLFHDKESM
PvSuS1      LQFKEQLVEEGPNNNFVLELDFEPFNASFPRPSLSKSIGNGVQFLNRHLSSKLFHDKESM
PvSuS2      LAFKEQLVDGHNNSNFVLELDFEPFNASFPRPSMSKSIGNGVQFLNRHLSSKLFQDKESL
            * ******:   . :******:****** ****:*** *:******** *****:****:

SoSUS2      YPLLNFLKAHNYKGTTMMLNDRIQSLRGLQSSLRKAEEYLLSVPQDTPYSEFNHRFQELG
OsSUS2      YPLLNFLKAHNHKGTTMMLNDRIQSLRGLQSSLRKAEEYLMGIPQDTPYSEFNHRFQELG
OsSUS3      YPLLNFLRAHNYKGMTMMLNDRIRSLDALQGALRKAEKHLAGITADTPYSEFHHRFQELG
ZmSUS1      YPLLNFLRAHNYKGMTMMLNDRIRSLSALQGALRKAEEHLSTLQADTPYSEFHHRFQELG
BoSUS1      YPLLNFLRAHNYKGMTMMLNDRIRSLSALQGALRKAEEHLSGLSADTSYSDFHHRFQELG
SbSUS2      YPLLNFLKAHNYKGTTMMLNDRIQSLRGLQSSLRKAEEYLLSVPQDTPYSEFNHRFQELG
LpSUS       YPLLNFLKGHNYKGTTMILNDRIQSLRGVQSALRKAEEYLVSIPEDTPSSEFNHRFQELG
BoSUS4      YPLLNFLKAHNPKGKTMMLNDRIQSLRGLQSALRKAEEYLISIPQDTPCSEFNHRFQELG
BoSUS2      YPLLNFLKAHNHKGTTMMLNDRIQSLRGLQSALRKAEEYLMSFPQDTPYSEFNHRFQELG
BoSUS3      YPLLNFLRAHNYKGMTMMLNDRIRSLSALQGALRKAEEHLSGLSADTPYSDFHHRFQELG
PvSuS1      YPLLNFLRAHNYKGMTMMLNDRIRSLSALQGALRKAEEHLSSLPADTPYSDFHHRFQELG
PvSuS2      YPLLNFLKAHNYKGTTMMLNDRIQSLRGLQSSLRKAEEYLLSIPQDTPYSEFNHRFQELG
            *******:.** ** **:*****:** .:*.:*****::*  .  **. *:*:*******

SoSUS2      LEKGWGDTAKRVLDTLHLLLDLLEAPDPANLEKFLGTIPMMFNVVILSPHGYFAQSNVLG
OsSUS2      LEKGWGDCAKRVLDTIHLLLDLLEAPDPANLEKFLGTIPMMFNVVILSPHGYFAQSNVLG
OsSUS3      LEKGWGDCAQRVRETIHLLLDLLEAPEPSALEKFLGTIPMVFNVVILSPHGYFAQANVLG
ZmSUS1      LEKGWGDCAKRAQETIHLLLDLLEAPDPSTLEKFLGTIPMVFNVVILSPHGYFAQANVLG
BoSUS1      LEKGWGDCAKRAQETIHLLLDLLEAPDPSTLEKFLGTIPMVFNVVILSPHGYFAQANVLG
SbSUS2      LEKGWGDTAKRVLDTLHLLLDLLEAPDPANLEKFLGTIPMMFNVVILSPHGYFAQSNVLG
LpSUS       LEKGWGDTAKRVHDTIHLLLDLLEAPDPASLEKFLGTIPMMFNVVILSPHGYFAQSNVLG
BoSUS4      LEKGWGDTAKRVLDTIHLLLDLLEAPDPANLEKFLGTIPMTFNVVILSPHGYFAQSNVLG
BoSUS2      LEKGWGDTAKRVLDTIHLLLDLLEAPDPANLEKFLGTIPMTFNVVILSPHGYFAQSNVLG
BoSUS3      LEKGWGDCAKRAQETIHLLLDLLEAPDPSTLEKFLGTIPMVFNVVILSPHGYFAQANVLG
PvSuS1      LEKGWGDCAKRAQETIHLLLDLLEAPDPSTLEKFLGTIPMVFNVVILSPHGYFAQANVLG
```

```
PvSuS2      LEKGWGDTAKRVLDTLHLLLDLLEAPDPANLEKFLGTMPMMFNVVILSPHGYFAQSNVLG
            ******* *:*. :*:**********:*: *******:** **************:****

SoSUS2      YPDTGGQVVYILDQVRALENEMLLRIKQQGLDITPKILIVTRLLPDAVGTTCGQRLEKVI
OsSUS2      YPDTGGQVVYILDQVRALENEMLLRIKQQGLDITPKILIVTRLLPDAVGTTCGQRVEKVI
OsSUS3      YPDTGGQVVYILDQVRAMENEMLLRIKQQGLNITPRILIVTRLLPDAHGTTCGQRLEKVL
ZmSUS1      YPDTGGQVVYILDQVRAMENEMLLRIKQCGLDITPKILIVTRLLPDATGTTCGQRLEKVL
BoSUS1      YPDTGGQVVYILDQVRAMENEMLLRIKQQGLNITPRILIVTRLLPDATGTTCGQRLEKVL
SbSUS2      YPDTGGQVVYILDQVRALENEMLLRIKQQGLDITPKILIVTRLLPDAVGTTCGQRLEKVI
LpSUS       YPDTGGQVVYILDQVRALENEMLLRIKQQGLDITPKILIVTRLLPDAVGTTCGQRLEKVI
BoSUS4      YPDTGGQVVYILDQVRALENEMLLRIKQQGLDITPKILIVTRLLPDAVGTTCGQRLEKVL
BoSUS2      YPDTGGQVVYILDQVRALENEMLLRIKQQGLDITPKILIVTRLLPDAVGTTCGQRLEKVI
BoSUS3      YPDTGGQVVYILDQVRAMENEMLLRIKQQGLNITPRILIVTRLLPDATGTTCGQRLEKVL
PvSuS1      YPDTGGQVVYILDQVRAMENEMLLRIKQCGLDITPKILIVTRLLPDATGTTCGQRLEKVL
PvSuS2      YPDTGGQVVYILDQVRALENEMLLRIKQQGLDITPKILIVTRLLPDAVGTTCGQRLEKVI
            *****************:********** **:***:*********** *******:***:

SoSUS2      GTEHTDIIRIPFRNENGILRKWISRFDVWPYLETYTEDVASEIMLEMQAKPDLIVGNYSD
OsSUS2      GTEHTDILRVPFRSENGILRKWISRFDVWPFLETYTEDVANEIMREMQAKPDLIIGNYSD
OsSUS3      GTEHTHILRVPFRTENGTVRKWISRFEVWPYLETYTDDVAHEISGELQATPDLIIGNYSD
ZmSUS1      GTEHCHILRVPFRTENGIVRKWISRFEVWPYLETYTDDVAHEIAGELQANPDLIIGNYSD
BoSUS1      GTEHTHILRVPFRTENGIVRKWISRFEVWPYLETFTDDVAHEIAGELQANPDLIIGNYSD
SbSUS2      GTEHTDIIRIPFRNENGILRKWISRFDVWPYLETYTEDVASEIMLEMQAKPDLIVGNYSD
LpSUS       GTEHTDILRVPFRTENG-IRKWISRFDVWQYLETYTEDVANELMREMQTKPDLIIGNYSD
BoSUS4      GTEHTDILRVPFRTENGILRKWISRFDVWPFLETYTEDVANEIMREMQAKPDLIIGNYSD
BoSUS2      GTEHTDILRVPFRTENGILRKWISRFDVWPFLETYTEDVANEIMREMQAKPDLIIGNYSD
BoSUS3      GTEHTHILRVPFRTENGIVRKWISRFEVWPYLETFTDDVAHEIAGELQANPDLIIGNYSD
PvSuS1      GTEHCHILRVPFRTENGIVRKWISRFEVWPYLETYTDDVAHEIAGELQANPDLIIGNYSD
PvSuS2      GTEHTDIIRVPFRNENGILRKWISRFDVWPYLETYTEDVASEIMKEMQAKPDLIIGNYSD
            **** .*:*:***.*** :*******:** :***:*:*** *:  *:*:.****:*****

SoSUS2      GNLVATLLAHKLGVTQCTIAHALEKTKYPNSDIYLDKFDSQYHFSCQFTADLIAMNHTDF
OsSUS2      GNLVATLLAHKLGVTQCTIAHALEKTKYPNSDIYLDKFDSQYHFSCQFTADLIAMNHTDF
OsSUS3      GNLVRCLLAHKLGVTHCTIAHALEKTKYPNSDLYWKKFEDHYHFSCQFTADLIAMNHADF
ZmSUS1      GNLVACLLAHKMGVTHCTIAHALEKTKYPNSDLYWKKFEDHYHFSCQFTTDLIAMNHADF
BoSUS1      GNLVACLLAHKMGVTHCTIAHALEKTKYPNSDLYWKKFEDHYHFSCQFTTDLIAMNHADF
SbSUS2      GNLVATLLAHKLGVTQCTIAHALEKTKYPNSDIYLDKFDSQYHFSCQFTADLIAMNHTDF
LpSUS       GNLVATLLAHKLGVTQCTIAHALEKTKYPNSDIYLDKFDSQYHFSCQFTADLIAMNHTDF
BoSUS4      GNLVATLLAHKLGVTQCTIAHALEKTKYPNSDIYLDKFDSQYHFSCQFTADLIAMNHTDF
BoSUS2      GNLVATLLAHKLGVTQCTIAHALEKTKYPNSDIYLDKFDSQYHFSCQFTADLIAMNHTDF
BoSUS3      GNLVACLLAHKMGVTHCTIAHALEKTKYPNSDLYWKKFEDHYHFSCQFTTDLIAMNHADF
PvSuS1      GNLVACLLAHKMGVTHCTIAHALEKTKYPNSDLYWKKFEDHYHFSCQFTTDLIAMNHADF
PvSuS2      GNLVATLLAHKLGVTQCTIAHALEKTKYPNSDIYLDKFDSQYHFSCQFTADLIAMNHTDF
            ****  *****:***:****************:* .**:.:********:*******:**

SoSUS2      IITSTFQEIAGSKDTVGQYESHIAFTLPGLYRVVHGIDVFDPKFDIVSPGADMSVYYPYT
OsSUS2      IITSTFQEIAGSKDTVGQYESHIAFTLPGLYRVVHGIDVFDPKFNIVSPGADMSVYFPYT
OsSUS3      IITSTFQEIAGNKETVGQYESHMAFTMPGLYRVVHGIDVFDPKFNIVSPGADMSIYFPFT
ZmSUS1      IITSTFQEIAGNKDTVGQYESHMAFTMPGLYRVVHGIDVFDPKFNIVSPGADLSIYFPYT
BoSUS1      IITSTFQEIAGNKDTVGQYESHMAFTMPGLYRVVHGIDVFDPKFNIVSPGADLSIYFPYT
SbSUS2      IITSTFQEIAGSKDTVGQYESHIAFTLPGLYRVVHGIDVFDPKFNIVSPGADMSVYYPYT
LpSUS       IITSTFQEIAGSKDSVGQYESHIAFTLPDLYRVVHGIDVFDPKFNIVSPGADMTVYFPYT
BoSUS4      IITSTFQEIAGSKDTVGQYESHIAFTLSGLYRVVHGIDVFDPKFNIVSPGADMSVYFPYT
BoSUS2      IITSTFQEIAGSKDTVGQYESHIAFTLPGLYRVVHGIDVFDPKFNIVSPGADMSVYFPYT
BoSUS3      IITSTFQEIAGNKDTVGQYESHMAFTMPGLYRVVHGIDVFDPKFNIVSPGADMSIYFPYS
PvSuS1      IITSTFQEIAGNKDTVGQYESHMAFTMPGLYRVVHGIDVFDPKFNIVSPGADMSIYFPYT
PvSuS2      IITSTFQEIAGSKDTVGQYESHIAFTLPGLYRVVHGIDVFDPKFNIVSPGADMSVYYPYT
```

```
               ***********.*::*******:***:..***************:*******:::*:*::

SoSUS2         ETDKRLTAFHPEIEELIYSDVENDEHKFVLKDKNKPIIFSMARLDRVKNMTGLVEMYGKN
OsSUS2         EADKRLTAFHPEIEELLYSEVENDEHKFVLKDKNKPIIFSMARLDRVKNMTGLVEMYGKN
OsSUS3         ESQKRLTSLHLEIEELLFSDVENTEHKFVLKDKKKPIIFSMARLDHVKNLTGLVELYGRN
ZmSUS1         ESHKRLTSLHPEIEELLYSQTENTEHKFVLNDRNKPIIFSMARLDRVKNLTGLVELYGRN
BoSUS1         ESHKRLTSLHPEIEELLYSDVDNHEHKFVLKDRNKPIIFSMARLDRVKNLTGLVELYGRN
SbSUS2         ETDKRLTAFHPEIEELIYSDVENDEHKFVLKDKNKPIIFSMARLDRVKNMTGLVEMYGKN
LpSUS          ETDKRLTAFHSEIEELLYSDVENDEHKFVKKDRNKPIIFSMARLDRVKNMTGLVEMYGKN
BoSUS4         ETDKRLTAFHPEIEELIYSDVENSEHQFVLKDKNKPIIFSMARLDRVKNMTGLVEMYGKN
BoSUS2         ETDKRLTAFHPEIEELIYSDVENSEHKFVLKDKNKPIIFSMARLDRVKNMTGLVEMYGKN
BoSUS3         ESHKRLTSLHPEIEELLYSDVDNNEHKFVLKDRNKPIIFSMARLDRVKNLIGLVELYGRN
PvSuS1         ESHKRLTSLHPEIEELLYSQTENNEHKFVLNDRNKPIIFSMARLDRVKNLTGLVELYGRN
PvSuS2         ETDKRLTAFHPEIEELIYSDVENSEHKFVLKDKNKPIIFSMARLDRVKNMTGLVEMYGKN
               *:.****::* *****::*:.:* **:** :*::***********:***: ****:**:*

SoSUS2         ARLRELANPVIVAGDHGKESKDREEQAEFKKMYSLIDEYNLKGHIRWISAQMNRVRNAEL
OsSUS2         AHLRDLANLVIVCGDHGNQSKDREEQAEFKKMYGLIDQYKLKGHIRWISAQMNRVRNGEL
OsSUS3         PRLQELVNLVVVCGDHGKESKDKEEQAEFKKMFNLIEQYNLNGHIRWISAQMNRVRNGEL
ZmSUS1         KRLQELVNLVVVCGDHGNPSKDKEEQAEFKKMFDLIEQYNLNGHIRWISAQMNRVRNGEL
BoSUS1         PRLQELVNLVVVCGDHGNPSKDKEEQAEFQKMFDLIEQYNLNGHIRWISAQMNRVRNGEL
SbSUS2         ARLRELANLVIVAGDHGKESKDREEQAEFKKMYSLIDEYNLKGHIRWISAQMNRVRNAEL
LpSUS          AHLKDLANLVIVAGDHGKESKDREEQAEFKRMYSLIEEYKLKGHIRWISAQMNRVRNGEL
BoSUS4         AHLRDLANLVVVAGDHGKESKDREEQAEFKRMYSLIEEYKLKGHIRWISAQMNRVRNGEL
BoSUS2         AHLRDLANLVIVAGDHGKESKDREEQAEFKRMYSLIDEYKLKGHIRWISAQMNRVRNGEL
BoSUS3         PRLQELVNLVVVCGDHGNPSKDKEEQAEFKKMFDHIEQYNLNGHIRWISAQMNRVRNGEL
PvSuS1         KRLQELVNLVVVCGDHGNPSKDKEEQAEFKKMFDLIEQYNLNGHIRWISAQMNRVRNGEL
PvSuS2         AHLRELANLVIVAGDHGKESKDREEQAEFKKMYSLIDQYNLKGHIRWISAQMNRVRNGEL
                 *::*.* *:*.****: ***:******::*:. *::*:*:***************.**

SoSUS2         YRYICDTKGAFVQPAFYEAFGLTVIESMTCGLPTIATCHGGPAEIIVDGVSGLHIDPYHS
OsSUS2         YRYICDTKGVFVQPAFYEAFGLTVIEAMTCGLPTIATCHGGPAEIIVDGVSGLHIDPYHS
OsSUS3         YRYICDMRGAFVQPALYEAFGLTVIEAMTCGLPTFATAYGGPAEIIVHGVSGYHIDPYQN
ZmSUS1         YRYICDTKGAFVQPAFYEAFGLTVVEAMTCGLPTFATAYGGPAEIIVHGVSGYHIDPYQG
BoSUS1         YRYICDTKGAFVQPAFYEAFGLTVVESMTCGLPTFATAYGGPAEIIVHGVSGFHIDPYQG
SbSUS2         YRYICDTKGAFVQPAFYEAFGLTVIESMTCGLPTIATCHGGPAEIIVDGVSGLHIDPYHS
LpSUS          YRYICDTKGAFVQPAFYEAFGLTVIEAMTCGLPTIATCHGGPAEIIVDGVSGLHIDPYHS
BoSUS4         YRYICDTRGVFVQPAFYEAFGLTVIESMTCGLPTIATCHGGPAEIIVDGVSGLHIDPYHS
BoSUS2         YRYICDTKGVFVQPAFYEAFGLTVIESMTCGLPTIATCHGGPAEIIVDGVSGLHIDPYHS
BoSUS3         YRYICDTRGAFVQPAFYEAFGLTVVESMSCGLPTFATAYGGPAEIIVHGVSGFHIDPYQG
PvSuS1         YRYICDTQGAFVQPAFYEAFGLTVVEAMTCGLPTFATAYGGPAEIIVHGVSGYHIDPYQG
PvSuS2         YRYICDTKGAFVQPAFYEAFGLTVIESMTCGLPTIATCHGGPAEIIVDGCLVCTLTLTQ-
               ****** :*.*****:********:*:*:*****:**.:********.*     :   :

SoSUS2         DKAADILVNFFERCKADPSYWDKISQGGLQRIYEKYTWKLYSERLMTLTGVYGFWKYVSN
OsSUS2         DKAADILVNFFEKCKQDSTYWDNISQGGLQRIYEKYTWKLYSERLMTLTGVYGFWKYVSN
OsSUS3         DKASALLVEFFEKCQEDPNHWIKISQGGLQRIEEKYTWKLYSERLMTLSGVYGFWKYVTN
ZmSUS1         DKASALLVDFFDKCQAEPSHWSKISQGGLQRIEEKYTWKLYSERLMTLTGVYGFWKYVSN
BoSUS1         DKASALLVEFFEKCQQDHSHWTKISQGGLQRIEEKYTWKLYSERLMTLTGVYGFWKYVSN
SbSUS2         DKAADILVNFFEKCKADPSYWDKISQGGLQRIYEKYTWKLYSERLMTLTGVYGFWKYVSN
LpSUS          DKAADILVNFFEKSTADPSYWDKISQGGLKRIYEKYTWKLYSERLMTLTGVYGFWKYVSN
BoSUS4         DKAADILVNFFEKCKEDPTYWDKISLGGLKRIYEKYTWKLYSERLMTLTGVYGFWKYVSN
BoSUS2         NKAADILVNFFEKCKEDPTYWDKISQGGLKRIYEKYTWKLYSERLMTLTGVYGFWKYVSN
BoSUS3         DKASALLVEFFEKCQQDPAHWTKISQGGLQRIEEKYTWKLYSERLMTLTGVYGFWKYVSN
PvSuS1         DKASALLVDFFEKCKEDSSHWSKISQGGLQRIEEKYTWKLYSERLMTLTGVYGFWKYVSN
PvSuS2         ------------------------------------------------------------
```

```
SoSUS2      LERRETRRYLEMFYALKYRSLASAVPLSFD------------------------------
OsSUS2      LERRETRRYIEMFYALKYRSLASAVPLAVDGESTSK------------------------
OsSUS3      LDRRETRRYLEMLYALKYRKMATTVPLAIEGEASTK------------------------
ZmSUS1      LERRETRRYLEMLYALKYRTMASTVPLAVEGEPSSK------------------------
BoSUS1      LERRETRRYLEMLYALKYRTMASTVPLAVDGEPSSK------------------------
SbSUS2      LERRETRRYLEMFYALKYRSLASAVPLSYD------------------------------
LpSUS       LERRETRRYLEMFYALKYRSLAAAVPLAVAGESSGNLRGFGACRGRIHRAGGRSVGCAMI
BoSUS4      LERRETRRYLEMFYALKYRSLASAVPLAVDGDSVAK------------------------
BoSUS2      LERRETRRYLEMFYALKYRSLASAVPLAVDGDSAAN------------------------
BoSUS3      LERRETRRYLEMLYALKYRKMASTVPLAVEGEPSNK------------------------
PvSuS1      LERRETRRYLEMLYALKYRTMASTVPLAVEGEPSSK------------------------
PvSuS2      ------------------------------------------------------------


SoSUS2      ------------------------------------------------------
OsSUS2      ------------------------------------------------------
OsSUS3      ------------------------------------------------------
ZmSUS1      ------------------------------------------------------
BoSUS1      ------------------------------------------------------
SbSUS2      ------------------------------------------------------
LpSUS       CLPSFPFGLSVVGCAILCLGTFVRSWAVLVAGSCQPPRINACPWCSSFIREFNA
BoSUS4      ------------------------------------------------------
BoSUS2      ------------------------------------------------------
BoSUS3      ------------------------------------------------------
PvSuS1      ------------------------------------------------------
PvSuS2      ------------------------------------------------------
```

9) Submitted phylogenetic tree of gene family:

i)Main Phylogenetic tree:

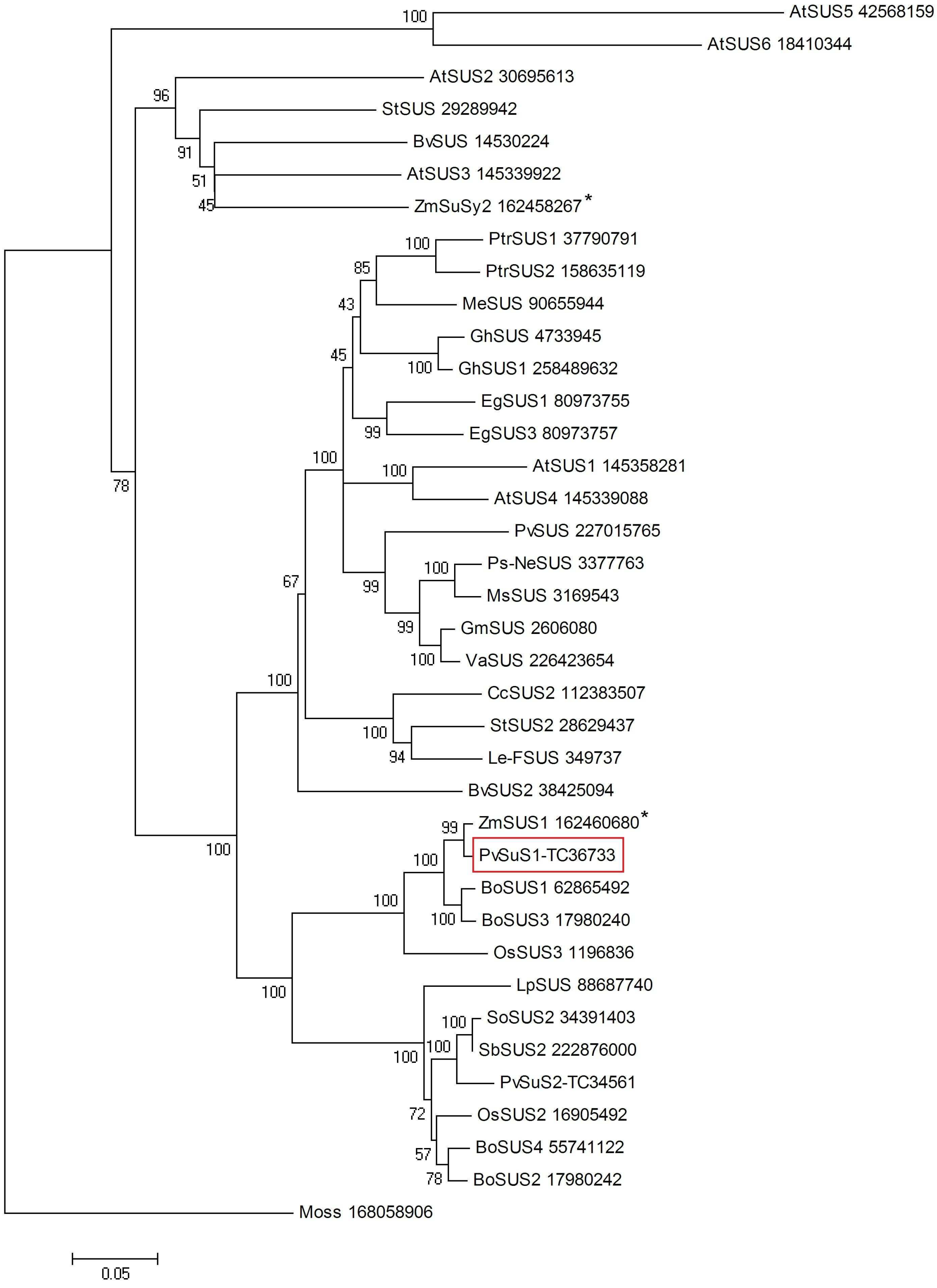

AtSUS5 42568159
AtSUS6 18410344
AtSUS2 30695613
StSUS 29289942
BvSUS 14530224
AtSUS3 145339922
ZmSuSy2 162458267*
PtrSUS1 37790791
PtrSUS2 158635119
MeSUS 90655944
GhSUS 4733945
GhSUS1 258489632
EgSUS1 80973755
EgSUS3 80973757
AtSUS1 145358281
AtSUS4 145339088
PvSUS 227015765
Ps-NeSUS 3377763
MsSUS 3169543
GmSUS 2606080
VaSUS 226423654
CcSUS2 112383507
StSUS2 28629437
Le-FSUS 349737
BvSUS2 38425094
ZmSUS1 162460680*
PvSuS1-TC36733
BoSUS1 62865492
BoSUS3 17980240
OsSUS3 1196836
LpSUS 88687740
SoSUS2 34391403
SbSUS2 222876000
PvSuS2-TC34561
OsSUS2 16905492
BoSUS4 55741122
BoSUS2 17980242
Moss 168058906
0.05

ii)Smaller Phylogenetic tree:

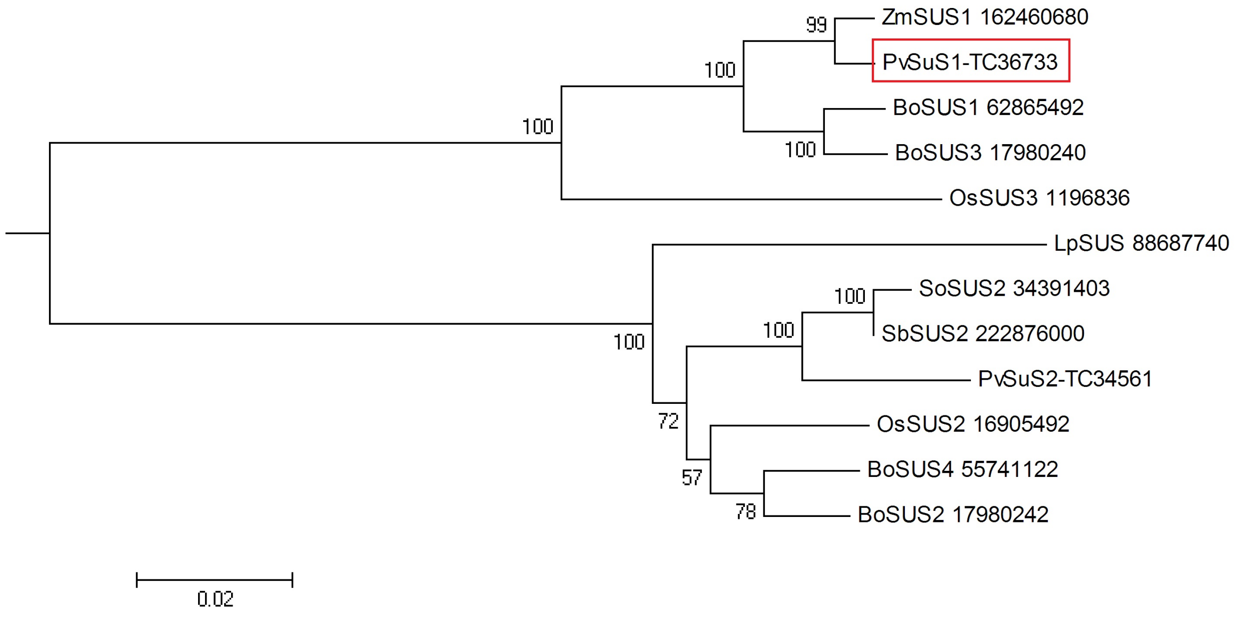

10) Description of phylogenetic tree:
Phylogenetic tree was constructed using amino acid sequences of sucrose synthase genes from 19 different species using clustalw (ver2.11).

11) Known paralogs, orthologs, co-orthologs, homologs:
The maize homolog of Switchgrass SUS1 is NP_001105323.1. Multiple sucrose synthase genes are known; however, sucrose synthase 1 (ZmSuS1) is the most relevant due to its membrane-bound form and relationship with cell wall biosynthesis.

12) Shared motifs and domains:

a) Shared motifs or domains among members of the candidate gene family:
1.IRWISAQMNRVRNGELYRYICDTKGAFVQPAFYEAFGLTV[IV]E[AS]MTCGLPT
2.YHFSCQFTADLIAMNH[AT]DFIITSTFQEIAG[SN]KDTVGQYESH[IM]AFT[LM]PGLY
3.KFLGTIPM[MV]FNVVILSPHGYFAQ[AS]NVLGYPDTGGQVVYILDQVRA[LM]ENEM
4.E[LM]QA[KNT]PDLIIGNYSDGNLVA[TC]LLAHK[LM]GVT[QH]CTIAHALEKTKYPNSD[IL]Y[LW]
5.KSIGNGVQFLNRHLSSKLF[QH]DKES[LM]YPLLNFL[KR]AHN[YH]KG[TM]TMMLNDRI[QR]S
6.[HY]WD[KN]ISQGGLQRI[YE]EKYTWKLYSERLMTLTGVYGFWKYVSNLERRETRRY

b) Unshared unique motifs or domains within sub-class family:

13) Analysis of transient or stable plant lines:

a) What will be measured?
All PCR-verified transgenic plants and controls will be evaluated by real-time RT-PCR to select the events/lines with higher gene expression levels. RT-PCR verified plants will be evaluated at whole plant level for morphological and sugar content changes.

b) Expression level of phenotype:
Recalictrance and MBMS screens, sugar release assays and wet chemistry will be performed at NREL on events/lines exhibiting the desirable phenotypes and modifications.

14) Proposed function
SUBSTRATE BIOSYNTHETIC

[1]Definition for stable non-plant expression: expressionn of a non-plant gene (e.g. bacterial gene) through stable integration into switchgrass or *populus*.

[2]Note: at least for the first part of 2008, VIGS will be done in foxtail millet (which initial results indicate is an excellent model system for switchgrass). Once VIGS is optimized in switchgrass, you will be able to choose between foxtail millet and switchgrass.
