# Supplementary material for: Development and use of a switchgrass (Panicum virgatum L.) transformation pipeline by the BioEnergy Science Center to evaluate plants for reduced cell wall recalcitrance
Source: Biotechnol Biofuels. 2017 Dec 22;10:309. doi: 10.1186/s13068-017-0991-x (PMC5740764; doi:10.1186/s13068-017-0991-x)
Supplement: Supplementary file 2 — Additional file 2. Switchgrass TP primers for listed genes. [file 13068_2017_991_MOESM2_ESM.docx]

| **BESC ID^[[1]](#endnote-1)^** | **Gene name** | **Gene ID^[[2]](#endnote-2)^** | **primer name** | **Forward primer sequence^[[3]](#endnote-3)^** | **primer name** | **Reverse primer sequence^c^** |
| --- | --- | --- | --- | --- | --- | --- |
| 180 | *4-Coumarate: coenzyme A ligase* (4CL) | EU491511 | Pv4CL_H3RI_For | caccaagcttgaattcGGTGTTCCGGTCGAAGCTG | Pv4CL SalXba_Rev | GCTCTAGAGTCGACGAACTCCCGCACCTTGTCC |
| 182 | *Cinnamic acid 4-hydroxylase* (C4H) | JX845712.1 | C4H_5R-7_RNAi_F | CACCCGCAACGTCGTCTTCG | C4H_5R-7_RNAi_R | TGAGGTCGTGGCATCGGTTG |
| 184-1^[[4]](#endnote-4)^ | *Hydroxycinnamoyl CoA: shikimate hydroxycinnamoyl transferase* (HCT1) | AP13ISTG44531 | HCT1RNAi-F | CTAATCTATGAGCTATAAAC | HCT1RNAi-R | CAAAATCTAGAGCTCTTTAC |
| 184-2^d^ | *Hydroxycinnamoyl CoA: shikimate hydroxycinnamoyl transferase* (HCT2) | AP13CTG44233 | HCT2RNAi-F | CAAACGCGCGGGGGGCCACAA | HCT2RNAi-R | GACGAACCAGAGCAAACGGA |
| HCT 1 & 2 | *Hydroxycinnamoyl CoA: shikimate hydroxycinnamoyl transferase 1&2*  (HCT1&2) | AP13ISTG44531 and AP13CTG44233 | HCT1/2RNAi-F | TCGGAGATGGTGTACCCGG | HCT1/2RNAi-R | GATGTCGTCGGTGTACTCGA |
| 186 | *Caffeoyl CoA 3-O-methyl transferase* (CCoAOMT) | KanlCTG10855 | CCOMT-RNAi6F | CACCTTCGACTTCGTCTTCG | CCOMT-ORF-R1 | CGTCTATGATTGGAGTTGGGC |
| 264 | *Coumaroyl shikimate 3′-hydroxylase* (C3′H) | AP13ISTG41630 | C3H-RNAi5F | CACCCTGAAAGAGCAGTATG | C3H-RNAi5R | TTGTACAGCTCCTCGTTCTC |
| 294 | *R2R3-MYB transcription factor* (MYB4) | AP13ISTG63786 | PvMYB_6497.F | CACCATGGGGCGGTCGCCGTGCTG | PVMYB_6497.R | TCAAACAAAAAAAAACAGCCCAAC |
| 318 | *Gibberellin 20-oxidase* (GA20-ox) | AP13ISTG41447 | 0318_g1_f | ATGGTGCAGGCTGCGCTTGATGA | 0318_g1_r | GAGTTGGTGGCTGTTGTTGCCATGGATG |
| 319 | *Gibberellin 20-oxidase* (GA20-ox) | AP13ISTG41447 | Ga20ox2_skd_2F_New | TGCGCCTCAACTACTACCC | Ga20ox2_skd_2R_New | CCTGTAGTGCCTCATGGTGA |
| 324 | *Peroxidase-30* | AP13CTG32230 | 0324_g1_f | ATGGCGAGAATGGCGGTGGTGG | 0324_g1_r | GTGCTTGTTGACGATGGCGCAGTGC |
| 327 | *Dirigent* | AlamSGLT18438 | dirigent/sg/f | ATGGCCAGTCCGACCCAATTCC | dirrigent/Pv/Reverse | GGCTAGTGATGTGTAGACTGCGTA |
| 328 | *Dirigent* | AlamSGLT18438 | dirigent/sg/f | ATGGCCAGTCCGACCCAATTCC | dirrigent/Pv/Reverse | GGCTAGTGATGTGTAGACTGCGTA |
| 343 | *Peroxidase-1* | AP13CTG30491 | Pv PerF2_kd | AGGACCTCGTCGTGCTCTCCG | Pv Per1 R1 | CTAGTTGACGAGGTAGCATTTGTTC |
| 348 | *NAC transcription factor* (NAC-AP2) | AP13_model_12336.m00003/ KanlCTG14756 | NAC-AP2-F | TTAGATCTATGCCGAGCGCGACTA | NAC-AP2-R | TTGGTAACCCTACTGCATCTCGAGGAATGGAT |
| 349 | *NAC transcription factor* (NAC-AP2) | AP13_model_12336.m00003/ KanlCTG14756 | Ri-B8-F1 | CACCTACTCAATCACCGATCTCCTCA | Ri-B8-R1 | ATCTCGAGGAATGGATAGCTCA |
| 356 | *Dirigent-2* | AlamSGLT17912 | Dir2 F1 | GACTCCTTGCTAGCCATGG | Dir2 R1 | CGATCGTAGTCCTCAAGGTTT |
| 413 | *Sucrose synthase 1* (SUS1) | KanlCTG00032 | 0413_g1_f | ATGGGGGAAGCTGCCGGCGACCGC | 0413_g1_r | TCACTTGCTGGAGGGCTCTCCCTC |
| 540 | *Cellulose synthase like; subfamily D* (CslD4) | AP13CTG14119ST | TPC540_SOE-F1 | TCC GCG AAC ATG TCC TGC A | TPC540_SOE-R1 | CGG GAA GCT GAA CCC ACC |
| 543 | *Cellulose synthase like; subfamily J* (CslJ) | AP13CTG17444_1 | 543_oe-F | GGCGAGATGCCGCCGTTCA | 543-R-2 | ACAGCATCGACTGCGACA |
| 549 | *Cellulose synthase like; subfamily F* (CslF6) | AP13ISTG44510 | TPC549_SOE-F2 | ATTATGGCGCCC GGCGGC | TPC549_SOE-R2 | GACGCATCCCTCCAACGGCCAGACC |
| 558 | *Cellulose synthase 8* (CesA8) | AP13CTG01684 | 558_oe-F | GGAGGAGGAGCCACGATGGAGT | 558_oe-R | CCCACAGCAGATGACTGTGTTG |
| 692 | *NAC transcription factor* (NAC-AP2) | AP13_model_12336.m00003/ KanlCTG14756 | NAC-AP2-F: | TTAGATCTATGCCGAGCGCGACTA | NAC-AP2-R | TTGGTAACCCTACTGCATCTCGAGGAATGGAT |
| 693 | *Laccase 4* (Lac 4) | AP13CTG11594 | PvLac4-F1 | CGCCGTTCAACTACACGG | PvLac4-R | TTGGGAAGATCGGACGGT |
| 833 | *Knotted-like homeobox protein 1* (KN1) | AP13CTG17556 | KN-RNAiF | ATGGAGGAGATCACCCACCAC | KN-RNAiR | GACGTCGCCGGCGTAGAG |
| 834 | *Knotted-like homeobox protein 1* (KN1) | AP13CTG17556 | KNF1 | ATGGAGGAGATCACCCACC | KNR12 | GCCGAGCCGGTACAGCC |
| 835-1^d^ | *Gibberellin 2-oxidase* (GA2-ox) | KanlCTG23388 | 835_oe-F1 | ATGAACGACGACGACGATTCCAACC | 835_oe-R1 | CAGTGGGCCGCCTCCTTGGAT |
| 835-2^d^ | *Gibberellin 2-oxidase* (GA2-ox) | Pavir.Ba04002 | PvGA2ox5F | ATGAACGACGACGACGATTCCAACC | PvGA2ox5R | TCACAGTGGGCCGCCTCCTTGGAT |
| 837 | *Ethylene response factor/SHINE transcription factor* (ERF/SHN 1) | Pavir.Da00422 | PvSHN2F | ATGGTGCCGTCGAAGAAGAAGTTC | PvSHN2R | GATGACGAGGCTGCCTTCCAGC |
| 838 | *UTR6/ Nucleotide-sugar transporter* | KanlCTG20141 | 838_kd_F1 | TGCCTAGCAGCAAAGTTGAA | 838_kd_R1 | CTCATCCAAGGAGACGGTGT |
| 839 | *UTR6/ Nucleotide-sugar transporter* | KanlCTG20141 | 839_oe-F1 | ATGAACGGGGAGGTGGAATG | 839_oe-R2 | GCTGTAAAAACATACAGGAAGGA |
| 844 | *Laccase 17 like gene A* (LAC17a) | AP13CTG11594 | 844_kd-F1 | CACCAACGTGCTCCTCACCGCGAAG | 844_kd-R1 | TGGTGCCCTGCATCACCAGCTCCAC |
| 845 | *Laccase 17 like gene A* (LAC17a) | AP13CTG11594 | 845_oe-F1 | CACCATGGCCATGGCGCTCTCCTC | 845_oe-R1 | CAACGTTTCTGATCAGGTCTAAT |
| 846 | *Laccase 17 like gene B* (LAC17b) | AP13ISTG55350 | 846_oe-F1 | CACCATGGCTACCTCCTACCTTCCCCGT | 846_oe-R1 | CGTGTCGCCTTAGCATTTG |
| 847 | *Purple acid phosphatase 2* (PAP2) | AP13CTG01226 | 847_oe-F1 | CACCATGCACCCCGAAAACC | 847_oe-R1 | CAGCACCACTTAAGATTCCTC |
| 848 | *Caffeoyl CoA 3-O-methyl transferase 2* (CCoAOMT2) | KanlCTG01916 | CCOMT2-RNAi4F | CACCGGCATCGACAGCAGCAAC | CCOMT2-RNAi4R | CCACAGCGTGTTGTCGTACACG |
| 849 | *Coumaroyl shikimate 3′-hydroxylase 2* (C3′H2) | AP13ISTG55845 | C3H22-SpeIF | ACTAGTATGAACGCGGCGGCCTCCTTC | C3H2-RNAi-3R | CACCAAGGGCTTACCTTCATTGC |
| 859 | *Basic helix-loop-helix transcription factor* (bHLH1) | AP13CTG05881 | 859_oe-F1 | ATGTATTCCCCTCCCTGCACCGAT | 859_oe-R1 | ACATCCCGTATGGTTCTGGACG |
| 863 | *Xyloglucan endotransglucosylase/hydrolase* (XTH-like2a) | AP13ISTG54783 | 863+70_kd-F3 | ACCGTCACCTCCTTCTACCTG | 863+70_kd-R3 | GTAGGGGAGGTCGTCGTAGC |
| 864 | *Xyloglucan endotransglucosylase/hydrolase* (XTH-like2a) | AP13ISTG54783 | 864_oe-F1 | ATGGCGCGCCGGTCTCTAGCT | 864_oe-R1 | ACATTCGCGGCTGCACTCGC |
| 870 | *Xyloglucan endotransglucosylase/hydrolase* (XTH-like1b) | AP13CTG28985 | TPC 870 F | AGCTGAGCGACACGAGCTAC | TPC 870 R | AGGACGCTGACGGAAGATTA |
| 871 | *Xyloglucan endotransglucosylase/hydrolase* (XTH-like1b) | AP13CTG28985 | TPC 871 F2 | GAGAAGAAGAAGATGAGGTCG | TPC 871 R4 | TAACCGGAGGTGGCA CTCGGTGG |
| 930 | *Caffeic acid 3-O-methyltransferase* (COMT) | HQ645965 | COMT-2-2-2_F | AGGTCCTCATGGAGAGCTGGTA | COMT-2-2-2_R | TGATCATGTCGACGTGGAAGAC |

1. ID, Identification number [↑](#endnote-ref-1)
2. Unique gene identifier [↑](#endnote-ref-2)
3. 5’ to 3’ sequence [↑](#endnote-ref-3)
4. Homologs of HCT or GA2-ox genes targeted after initial TP submission, designated by dashed numeral after BESC ID number. [↑](#endnote-ref-4)
