# Supplementary material for: Development and use of a switchgrass (Panicum virgatum L.) transformation pipeline by the BioEnergy Science Center to evaluate plants for reduced cell wall recalcitrance
Source: Biotechnol Biofuels. 2017 Dec 22;10:309. doi: 10.1186/s13068-017-0991-x (PMC5740764; doi:10.1186/s13068-017-0991-x)
Supplement: Supplementary file 3 — Additional file 3. Alignment of PvXTH-like genes selected for transformation and location of unique sequences for silencing individual family members. Diagram of PvXTH family member sequences and position of sequence fragments used to silence individual PvXTHs in switchgrass. Unique PvXTH-like1b for silencing XTH-1b expression is indicated in the red box and unique PvXTH-like2a sequence for silencing XTH-2a expression is indicated in blue box. Areas with identity between the sequences are highlighted. [file 13068_2017_991_MOESM3_ESM.pptx]

## Slide 1
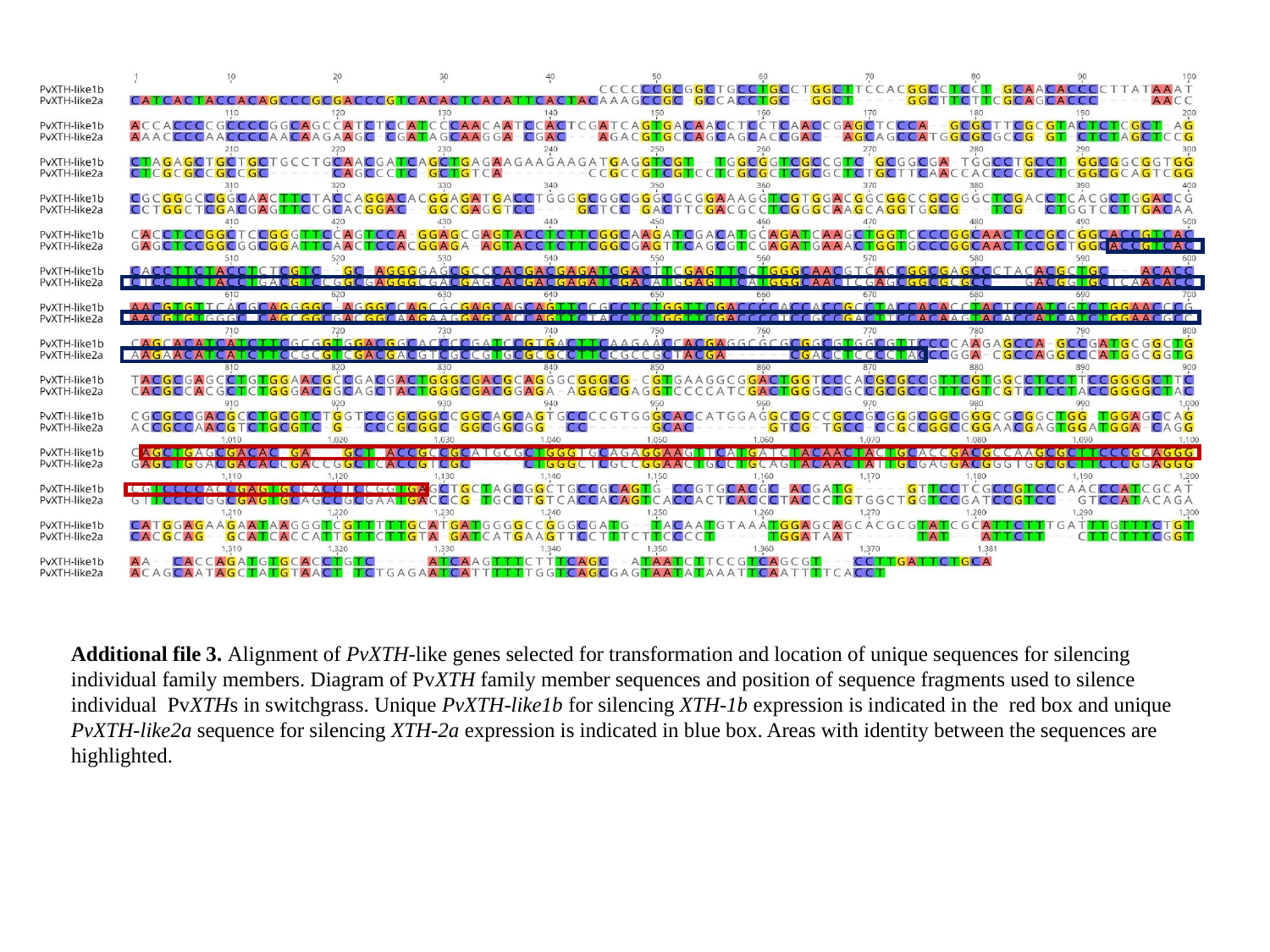

Additional file 3. Alignment of PvXTH-like genes selected for transformation and location of unique sequences for silencing individual family members. Diagram of PvXTH family member sequences and position of sequence fragments used to silence individual PvXTHs in switchgrass. Unique PvXTH-like1b for silencing XTH-1b expression is indicated in the red box and unique PvXTH-like2a sequence for silencing XTH-2a expression is indicated in blue box. Areas with identity between the sequences are highlighted.
