# Supplementary material for: Development and use of a switchgrass (Panicum virgatum L.) transformation pipeline by the BioEnergy Science Center to evaluate plants for reduced cell wall recalcitrance
Source: Biotechnol Biofuels. 2017 Dec 22;10:309. doi: 10.1186/s13068-017-0991-x (PMC5740764; doi:10.1186/s13068-017-0991-x)
Supplement: Supplementary file 4 — Additional file 4. Diagram illustrating position of three RNAi construct sequences used to silence HCTs in switchgrass. Sequences amplified and expressed in switchgrass to silence HCT1 (HCT1RNAi, purple box), HCT2 (HCT2RNAi, red box), and HCT 1 and 2 together (HCT1/2RNAi, blue box) are shown within the aligned PvHCT sequences. Areas with identity between the sequences are highlighted. [file 13068_2017_991_MOESM4_ESM.pptx]

## Slide 1
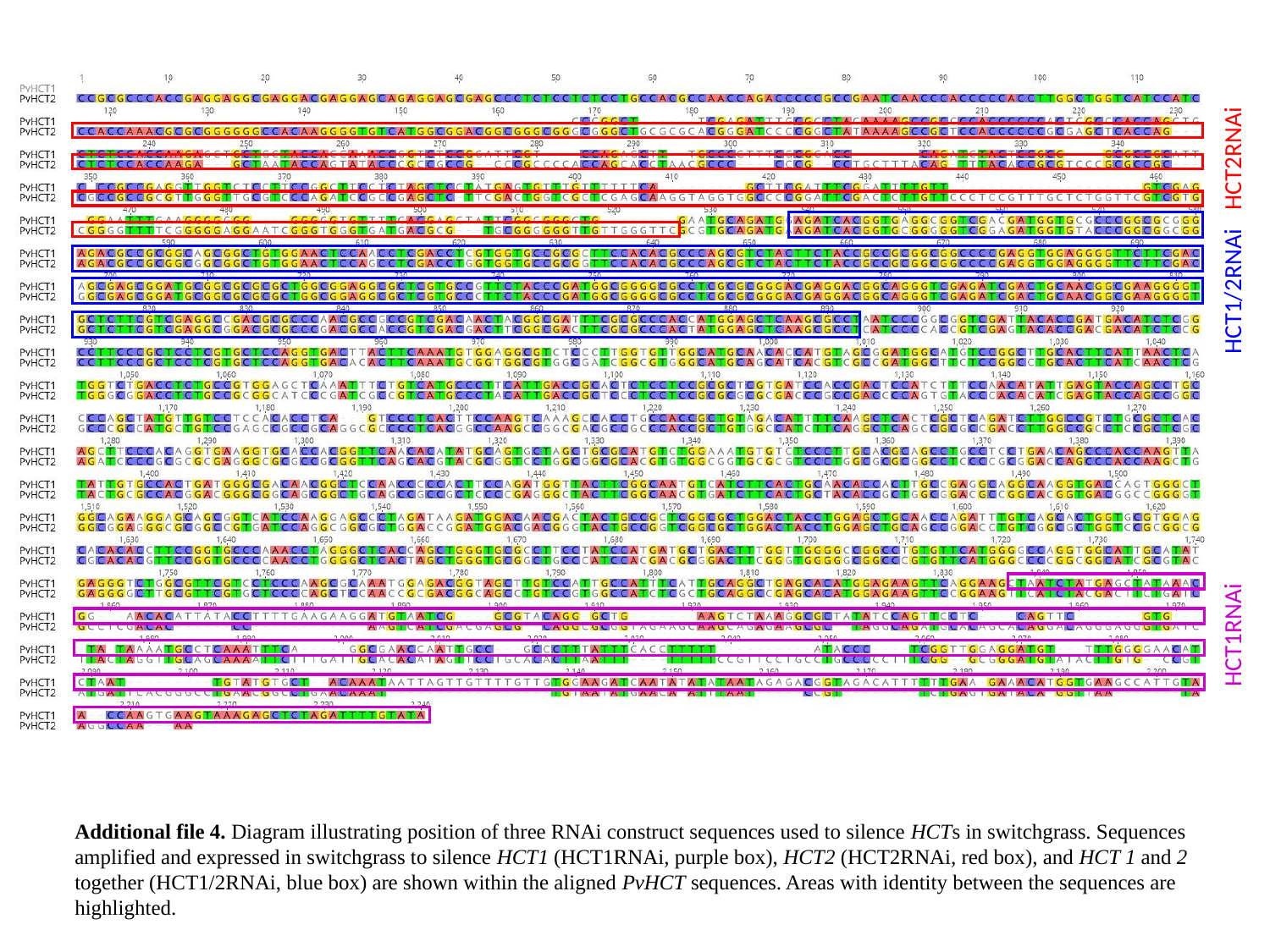

HCT2RNAi
HCT1/2RNAi
HCT1RNAi
Additional file 4. Diagram illustrating position of three RNAi construct sequences used to silence HCTs in switchgrass. Sequences amplified and expressed in switchgrass to silence HCT1 (HCT1RNAi, purple box), HCT2 (HCT2RNAi, red box), and HCT 1 and 2 together (HCT1/2RNAi, blue box) are shown within the aligned PvHCT sequences. Areas with identity between the sequences are highlighted.
